# The male sexual apparatus in the order Scorpiones (Arachnida): a comparative study of functional morphology as a tool to define hypotheses of homology

**DOI:** 10.1186/s12983-017-0231-z

**Published:** 2017-11-22

**Authors:** Lionel Monod, Lucie Cauwet, Edmundo González-Santillán, Siegfried Huber

**Affiliations:** 1 Département des arthropodes et d’entomologie I, Muséum d’histoire naturelle, Route de Malagnou 1, 1208 Genève, Switzerland; 2 Instituto de Biotecnología, UNAM, Avenida Universidad 2001, Colonia Chamilpa C.P., 62210 Cuernavaca, Morelos México

**Keywords:** Hemispermatophore, Spermatophore, Insemination, Bauplan, Natural selection, Sexual selection, Functional constraints, Phylogenetic value

## Abstract

**Background:**

Insemination in scorpions is carried out by means of a partly sclerotized structure, the spermatophore, which is composed of two separate halves, the hemispermatophores. In most genera these reproductive structures can be used to differentiate species. However, many taxa such as the genus *Euscorpius* and the family Diplocentridae lack the morphological diversity observed in the copulatory organs of many other arthropods, rendering them useless for species level taxonomy. Such structural stasis, however, suggests that hemispermatophores have evolved relatively slowly and may thus provide a stronger phylogenetic signal for recognizing supra-generic ranks than previously thought. Based on the postulate that the phenotypic stability observed in some groups is the consequence of functional constraint, the most comprehensive comparative study of the male sexual apparatus to date was conducted for a complete reassessment of the morphology, phylogenetic value and hypotheses of homology of these structures.

**Results:**

Hemispermatophores, pre- and post-insemination spermatophores, as well as the inherent mechanisms of insemination, were studied across the whole order, allowing the recognition and description of a series of five basic bauplans for the capsular region. For the most part, these patterns appear to be consistent within each major taxonomic group, but several cases of incongruence between spermatophore morphology and taxonomy raises questions about the monophyly of some clades. The Bothriuridae are traditionally regarded as a basal scorpionoid family. However, except for the genus *Lisposoma*, bothriurid hemispermatophores and spermatophores are morphologically more similar to those of the Chactoidea than to those of scorpionoids. On the other hand, the male copulatory structures of the hormurid clade (*Hormiops* (*Hormurus + Liocheles*)) are more akin to those of Diplocentridae and Heteroscorpionidae than to those of other hormurids.

**Conclusions:**

Spermatophore capsular patterns appears to be congruent with a recent phylogeny of the order Scorpiones based on phylogenomic data that placed Bothriuridae outside of Scorpionoidea and *Liocheles* outside of Hormuridae, in contradicton with earlier phylogenetic reconstructions based on morphology. This raises questions about the potential use of functionally constrained traits to assess the reliability of contradicting phylogenetic hypotheses and emphasizes the need for a thorough reassessment of the scorpion phylogenetic relationships.

**Electronic supplementary material:**

The online version of this article (10.1186/s12983-017-0231-z) contains supplementary material, which is available to authorized users.

## Background

### Morphology of the scorpion reproductive apparatus

In scorpions, sperm is not transferred directly by a sexual organ, but using a spermatophore, an external, partly sclerotized structure containing the spermatozoa, which is produced and deposited on the ground by the male [1]. This spermatophore is composed of two halves, the hemispermatophores, which are secreted by the paraxial organs [2] and joined together when they are expelled from the body during courtship.

**Fig. 1:**
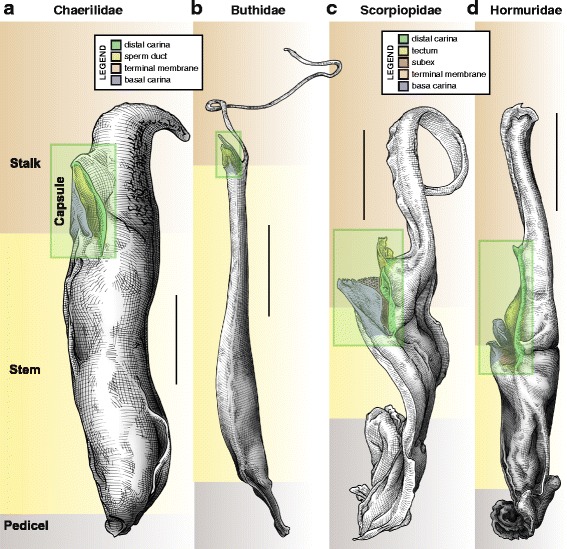
Hemispermatophore in toto, lateral aspect, groundplan. The four parts of the structure (stalk, stem, pedicel and capsule) are indicated for each of the four distantly related taxa, emphasizing the conserved pattern despite the variable proportions. **a**, Chaerilidae Pocock, 1893. **b**, Buthidae Koch, 1837. **c**, Chactidae Pocock, 1893. **d**, Hormuridae Laurie, 1896. Scales, 0.5 mm (**a**), 1 mm (**c**), 2 mm (**d**), 3 mm (**b**)

The spermatophore is composed of four distinct parts (Fig. 1). Although their shapes and proportions can be extremely variable, each part has the same function in all taxa: (1) the pedicel is used to glue the spermatophore to the substrate, (2) the stem is composed of soft membranes and contains the spermatozoa, (3) the stalk (= flagellum and distal lamina sensu Lamoral [3]) is usually sclerotized and acts as a lever to trigger the compression of the stem in most taxa, and (4) the capsule is located between the stem and the stalk and guides semen from the spermatophore cavity to the female genital tract.

This last part, the capsule, is responsible for the sperm transfer per se. It is composed of two sclerotized ridges/carinae (distal and basal) delimiting a soft membrane, the “sperm duct” (sensu Stockwell [4]). The capsule shows the greatest structural variation in the spermatophore, ranging from simple openings to complex eversible structures.

### Hemispermatophores as taxonomic characters

Vachon [5] was the first to recognize the taxonomic value of hemispermatophore and to use it as a diagnostic character in the families Buthidae Koch, 1837 and Scorpionidae Latreille, 1802. In the sixties and seventies, San Martin [6–9], Maury [10–17] and Cekalovich [18–22] emphasized its importance for the systematics of Bothriuridae Simon, 1880. It was only in the late seventies/eighties that hemispermatophores started to be treated more widely as taxonomically informative characters in others families: initially in Hormuridae Laurie, 1896 [3, 4, 23–28] and Urodacidae Pocock, 1893 [4, 23], then in Caraboctonidae Kraepelin, 1905 [4, 29], Chactidae Pocock, 1893 [4, 29, 30], Iuridae Thorell, 1876 [29], Vaejovidae Thorell, 1876 [4, 29, 31–33], Superstitioniidae Stahnke, 1940 [34], Typhlochactidae Mitchell, 1971 [34], Heteroscorpionidae Kraepelin, 1905 [26, 35, 36] and Diplocentridae Karsch, 1880 [4, 37, 38]. The morphology of hemispermatophores from several families became known only recently: in 1989 for Chaerilidae Pocock, 1893 [4], Euscorpiidae Laurie, 1896 [3, 4, 39] and Scorpiopidae Kraepelin, 1905 [4]; in 2001 for Troglotayosicidae [40]; in 2005 for Hemiscorpiidae Pocock, 1893 [41]; in 2006 for Pseudochatidae Gromov, 1998 [42]. Nevertheless, despite the generalized use of hemispermatophore as a diagnostic character in taxonomic descriptions since the beginning of the 21rst Century, our knowledge for a few groups, i.e. Chactidae, Pseudochactidae, Typhlochactidae, Troglotayosicidae and Scorpiopidae, still remains fragmentary.

Although hemispermatophores are usually distinct for closely related species, their morphology can also be extremely uniform in some genera ([4, 43, 44]; Monod, unpublished data) or even in some families [4], conveying little to no taxonomic information at the species level. Such structural stasis suggests that hemispermatophores have evolved relatively slowly at least in some groups and may thus potentially provide a strong phylogenetic signal at higher taxonomic levels.

### Hypothesis of homology in hemispermatophores

Hemispermatophores have very rarely been considered in a more global evolutionary perspective because their study is usually purely descriptive. Lamoral [3] was the first to propose structural homologies among the hemispermatophores of Buthidae and Scorpionidae. He considered that the flagellum of buthids was an extension of the capsule and was therefore not homologous to the distal lamina of scorpionids. Stockwell [4] disagreed with this hypothesis, pointing out the very limited sampling of intermediate taxa examined by his predecessor and presented a more congruent hypothesis of homology based on comparison of a wider range of taxonomic groups. His work is thus far the only available study giving a comparative assessment of the morphology of hemispermatophores across the whole order. Several phylogenetic analyses [45–50] subsequently used Stockwell’s work to define characters and character states of hemispermatophores for their respective matrices, but each of these studies focused on infraordinal taxonomic groups rather than the order as a whole.

### Phylogenetic values and functional morphology of Hemispermatophores

Due to the high phenotypic dissimilarity seen in many organisms, the male sexual apparatus is widely used to diagnose species, but is also considered to have evolved too rapidly to be phylogenetically informative [51, 52]. However, numerous phylogenetic studies of various arthropod lineages have successfully included male genitalic characters, demonstrating that reproductive structures may also be useful in determining supra-specific phylogenetic relationships [53].

Scorpion hemispermatophores can be divided into different ‘subunits’ that are arguably subject to different selective pressures and thus show different patterns of evolution and therefore various degrees of phylogenetic value [54–56]. While some characters follow the general trend of rapid and divergent evolution driven by strong sexual selection observed in most male genitalia, others show only limited variation among closely related taxa, making them more suitable for the reconstruction of the deeper phylogenetic relationships [56].

Mattoni et al. [56] pointed out that the traits of spermatophores, which are mechanically constrained to perform a particular task, would be less likely to evolve rapidly and randomly than, for instance, features subject to sexual selection. Functional constraint limits the morphological variability, and when a structure is involved in performing a precise mechanistic task, its shape cannot be quickly and radically modified without causing severe functional disruption [57–60].

Based on this reasoning, slowly evolving mechanically constrained characters are potentially the most informative for reconstructing deeper phylogenetic nodes. It is therefore necessary to consider not only the morphology of hemispermatophores but also their functional aspect in order to correctly assess homology. In this respect, the study of the mechanisms of insemination by comparison of pre- and post-mating spermatophores over a wide range of taxa is of paramount importance in understanding how the different features that compose the male reproductive apparatus relate to each other in distant taxa.

### Post-Copulatory Spermatophores in Systematics

Despite their putative usefulness for systematics [54], pre- and post-copulatory spermatophores have been treated relatively marginally in the scorpion literature [1, 2, 4, 61–94], and only few studies present an analysis of functional morphology and the mechanisms involved. To our knowledge, spermatophore morphology has never been compared in detail across distantly related taxa and used in a phylogenetic study in the way that it has been for the order Amblypygi Thorell, 1883 [95, 96]. The limited data available on spermatophores is probably a consequence of the difficulty in obtaining these structures, necessitating as it does the availability of living adult animals of both sexes and the observation of successful matings. This gap in our knowledge of the reproductive structures in male scorpions has probably contributed to the difficulty of establishing reliable hypotheses of homology.

### Achievements of the present study

We present here the most comprehensive comparative study of the scorpion male reproductive apparatus, not limited to morphology of hemispermatophores but also including comparison of spermatophore morphology, an analysis of the mechanisms of insemination and of the function of the various structures. The largest database to date was gathered by the examination of museum material but also by searching the literature for all published illustrations of hemi- and spermatophores. Moreover, we doubled the data available for pre- and post-copulatory spermatophores and present the first comparative study of these structures over a range of distantly related taxa. This allow us (1) to elaborate a more consensual terminology applicable to the whole order, (2) to identify characters most suitable to provide strong phylogenetic signal at supra-specific phylogenetic levels, (3) to reassess homology hypotheses for hemispermatophores, with an emphasis on the capsule. Based on these new paradigms, we identify and describe five general patterns (bauplans) for the capsular region and map them onto available phylogenetic reconstructions in order to uncover inherent evolutionary trends. The roles of natural and sexual selection in the evolution of scorpion copulatory structures are then evaluated. Finally, conflicts between morphological and molecular phylogenies and the reliabilty of each hypothesis are also discussed based on congruence between these hypotheses and the reproductive morphology.

## Methods

### Acquisition of spermatophores

Live specimens were purchased from the pet trade, donated by colleagues or collected in the field in compliance with the legal requirements of each country. Spermatophores were obtained from breeding specimens during field expeditions or subsequently in the laboratory. Specimens were kept in captivity at temperatures between 25 and 30 °C, without seasonal changes. Each specimen was housed individually in a plastic container provided with a mixture of bark mulch and peat as substrate and pieces of cork bark for hiding. Each specimen was fed 2–4 crickets (*Acheta domestica* (Linnaeus, 1758), *Gryllomorpha dalmatina* (Ocskay, 1832), *Gryllus assimilis* (Fabricius, 1775) or *Gryllus bimaculatus* De Geer, 1773) or 1–2 cockroaches (*Blaptica dubia* Serville, 1838) every two weeks. Each enclosure was watered once a week or every two weeks depending on the moisture need of each taxon. For mating, a female and a male were placed in a larger plastic container with a thin sheet of cork bark on the bottom. In order to create a humidity gradient the cork bark was moistened on one side with a plant mister. When mating occurred, spermatophores were retrieved from the enclosure immediately after the pair separated and placed into 75% ethanol. In order to obtain pre-insemination spermatophores, the pairs were separated after the male had deposited the spermatophore on the substrate and the unused spermatophore was then placed in 75% ethanol.

### Dissection and examination

Spermatophores and hemispermatophores were examined with a ZEISS Stemi SV8 stereomicroscope. Mature male specimens were dissected using microsurgical scissors and forceps for extracting both of their hemispermatophores. Paraxial organ tissue was removed either manually with forceps or chemically with a solution of Proteinase K (concentration: 10 mg/mL; Qiagen, Venlo, The Netherlands). Chemical extraction was performed by immersing hemispermatophores in the solution and then placing them in an oven at 45*–*50 °C for 15 min to an hour depending on the size and sclerotization of the structure. Once the soft tissue of the paraxial organ was sufficiently digested, the hemispermatophores were retrieved from the solution and thoroughly rinsed with water.

Depending on the taxa, some parts of the hemispermatophore capsular region are composed of soft membranes that can be easily damaged during dissection whether chemical or manual. The thinner membranes can be degraded by long exposure to Proteinase K, or torn apart by strong traction of the forceps, especially after prolonged storage in alcohol which can stiffen them. In fresh specimens, these membranes remain flexible and are less likely to be damaged during dissection. Therefore, whenever possible the dissection was carried out immediately after the specimen was euthanized.

However, most of the time fresh specimens were not available. In those cases, the size and state of preservation of the specimen were evaluated in order to determine the best methods of extraction to minimize the risk of damaging the structure. Manual extraction was performed with relatively good success on large taxa, even those preserved for a long time, while chemical extraction was preferred for smaller taxa. On several occasions, several specimens of the same species were dissected in order to obtain a hemispermatophore showing the complete set of informative characters.

### Evaluation of insemination mechanism

The position of a spermatophore during mating was inferred by placing the spermatophore on a preserved female of the corresponding species, following the method used by Jacob et al. [90]. For taxa in which the spermatophore remains attached to the female gonopore for some time after copulation, i.e. the genus *Chiromachus*, and the sub-genera *Monodopisthacanthus* Lourenço, 2001 and *Nepabellus* Francke, 1974 (see corresponding paragraph for details), the position of the structure could be ascertained in situ directly after the mating occurred.

Assessment of the insemination processes also involved a precise localization of the capsular foramen, the opening through which the semen is expelled from the spermatophore. It is clearly visible under a microscope and always associated with the presence of sperm.

### Photographs and illustrations

Line drawings were produced using a drawing tube mounted on the SV8 stereomicroscope. Pencil sketches were subsequently inked and scanned for further processing and editing. High-resolution images were taken with a custom-built stacking system and with a DSLR camera equiped with a VariMag II DSLR microscope adapter system (CNC Supply, cape Coral, FL, U.S.A.) and mounted on a stereomicrocope. Zerene Stacker (Zerene Systems, Richland, WA, U.S.A.) was used to assemble images taken at different focal planes into a single image with greater depth of field. Illustrations and photographs were edited (background removal and contrast adjustment) in Adobe Photoshop CS5 and plates prepared with Adobe illustrator CS5 (both from Adobe systems, San Jose, CA, U.S.A.).

Line drawings are preferred over photographs and SEM to illustrate hemispermatophores for the following reasons: (1) the small size and transparency of the structure sometimes results in photographs uninterpretable in three dimensions, whereas drawing with a drawing tube enables a precise interpretation of the complex shape of the capsule; (2) drying a hemispermatophore for SEM often causes shrivelling or deformation, even when using critical point drying. For spermatophores, photographs were deemed appropriate because the transparency is less problematic than for hemispermatophores (spermatophores are filled with semen), and they are preferred over SEM because of the paucity of material available.

Rather than illustrating all hemispermatophores examined, major patterns were identified and representative taxa were selected. As explained above, it is difficult and time-consuming to obtain spermatophores, and each specimen provides crucial information that can potentially facilitate future work. Illustrations of spermatophores from most of the genera for which the structure was previously unknown are therefore presented, even when they belong to closely related taxa. When a hemispermatophore and a spermatophore were available for a given taxon, a plate representing both structures was prepared in order to allow a direct comparison of the two structures, which we considered necessary to understand the process of capsular eversion.

### Scanning electron microscopy

Spermatophores of *Hormiops davidovi* Fage, 1933 preserved in 75% ethanol were dehydrated in a graded alcohol series, critical point dried in a SPI-DRY critical point dryer (SPI supplies, West Chester, PA, U.S.A.), mounted on standard aluminium stubs (diameter 12.5 mm, height 6 mm; Agar Scientific, Essex, U.K.), and sputter-coated with gold in a Cressington Sputtercoater 108 Auto. The sample was examined with a Zeiss DSM940A SEM.

### Terminology of positional aspect of hemispermatophores

**Fig. 2:**
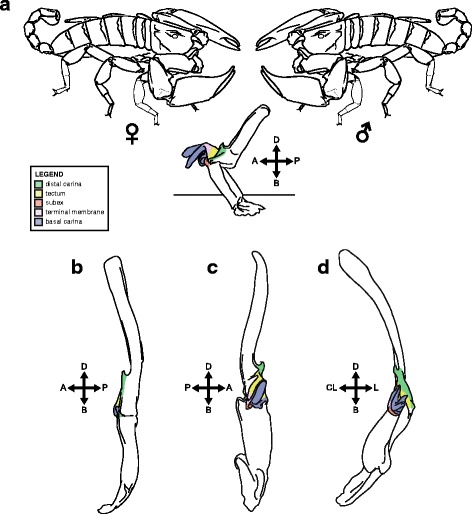
Positional nomenclature of the male reproductive structure. **a**, diagrammatic representation of the post-insemination spermatophore of *Liocheles* cf. *australasiae* Fabricius, 1775 (Thailand, Roi Et Province, MHNG), lateral aspect, indicating its position relative to male and female during copulation. **b**, diagrammatic representation of the pre-insemination spermatophore *Heterometrus mysorensis* Kovařík, 2004 (India, MHNG), lateral aspect. **c**-**d**, diagrammatic representation of the left hemispermatophore of *Heterometrus indus* (De Geer, 1778) (Sri Lanka, MHNG), contra-lateral (**c**) and anterior (**d**) aspects. Abbreviations: A (anterior), B (basal), D (distal), L (lateral), CL (contra-lateral) and P (posterior)

Lamoral [3] proposed a terminology to identify the different orientational aspects of the hemispermatophore based on the anatomical position of the structure inside the body prior to dissection. Stockwell [4], on the other hand, used the position of the functional spermatophore in relation to the position of the male during copulation as the reference system. Although most researchers subsequently used Lamoral’s terminology to describe hemispermatophores, Stockwell’s alternative is more appropriate, as pointed out by Cauwet [97]. Depending on the taxonomic group, hemispermatophore capsules may not have the same orientation inside the male body, and its orientation is, moreover, difficult to assess with accuracy without completely removing the tergites. On the other hand, the orientation of deposited spermatophores is always the same across the order, with the capsule and sperm duct always facing away from the male towards the female gonopore. Furthermore, contrary to Lamoral’s hemispermatophore terminology, Stockwell’s can be applied to both hemispermatophores and spermatophores. A second terminology for spermatophores is therefore not needed and direct comparison between the two structures is straightforward. The positional terminology of Stockwell [4] adapted by Cauwet [97] (Fig. 2) is thus used in the present contribution.

### Nomenclature of hemispermatophores

The tendency among scorpion taxonomists to use different nomenclatural systems, adapted to the particular group studied but not necessarily to other taxa, has arguably hampered the establishment of a consensual terminology based on reliable interpretations of homology [97]. The terminology used in the present paper is thus adapted from several nomenclatural systems, i.e. Vachon [5], Alexander [62, 63], San Martin [7], Lamoral [3], Francke [83], Stockwell [4], Peretti [85], Monod & Volschenk [98], Monod & Lourenço [41] and Monod [49]. Whenever possible, we have adopted the first term proposed. In some cases, however, older terms were considered less appropriate than more recent ones, either because they define the character concerned less accurately, or because a more recent name has gained common acceptance among researchers. Furthermore, several new names are introduced, some to designate newly recognized structures or parts, and others to designate features already described but for which all names available were considered inappropriate. For instance, numerous parts of hemispermatophore capsule have been referred to as lobes. This vague wording does not properly describe either morphology or function and was thus systematically replaced.

All the terms used in this publication and their synonymies with earlier nomenclatural systems are listed below. The synonyms are listed in chronological order with their references and a definition for each new name is provided. The list is divided into three sections corresponding to the main parts of the hemispermatophore (Fig. 1), i.e. the stalk, the capsule and the stem.

**Stalk**: The term proposed by Alexander [62] is retained here because it can be applied to both flagelliform and lamelliform hemispermatophores. Distal lamina is, however, still used in the present publication to designate the part of the stalk, distal to the transverse ridge, which usually bears a laminar hook, in lamelliform hemispermatophore. *Lame distale* [5];* Flagelle (pars recta + pars reflexa) (Buthidae)* [5];* Posterior process + blade (Buthidae)* [99];* Stalk* [62, 63]; *Flagellum (Buthidae)* [4, 68, 83]; *Blade* [69];* Flagella* [64];* Lámina distal* [6, 7, 9, 18, 76, 77, 100];* Distal lamina* [3, 49, 90];* Flagellum (Pars recta + pars reflecta + pars bireflecta)* [3];* Lamina (Lamella in text)* [83];* Distal part* [101];* Distal lamella* [4];* Flagelo (Buthidae)* [84];* Lámina* [85];* Lamina* [30, 86, 102];* Flagellum (Pseudochactidae)* [42].

- **Latero-distal crest** (Bothriuridae): *Cresta* [7, 9, 76, 77, 85]; *Crest* [86]; *Distal crest of lamina* [102].
- **Antero-distal crest of distal lamina**: *Lateral crest* [3]; *distal crest of distal lamina* [49].
- **Laminar hook**: *Lobe interne* [5];* Chitinous hook* [63]*; Lóbulo interno* [18]; *Protuberancia espiniforme* [76]; *Lóbulo distal* [9, 77]; *Lobulación dorsales esclerificadas del lobulió interno* [100]; *Hook* [3, 41, 49, 98]; *Distal lobe* [101]; *Dorsal fold* [86];* Basal lobe + Outer distal lobe + Inner distal lobe* [90]; *Dorsal apophysis* [30].
- **Transverse ridge**: *Distal crest of median lobe + Ectal crest of median lobe* [3]; *Sutura tronco-laminar* [85];* Laminar-trunk suture* [86];* Transverse ridge* [41, 49, 98].

**Capsule**: Part of the spermatophore between stalk and stem responsible for sperm transfer and composed of two sclerotized ridges/carinae (distal and basal) delimiting a soft membrane, the “sperm duct”. *Capsule* [62, 64, 83];* Capsule portion* [63];* Cápsula* [77];* Sperm tube (Buthidae)* [83];* Cápsula* [85];* Capsule* [42, 86, 90].

- **Sperm duct**: Membranous part of the capsule, eversible in many taxa. *Protuberance* [69]; *Median transverse trough (part)* [3]; *Outer lobe (Buthidae)* [3]; *Sperm duct* [4, 83]; *External lobe (Buthidae/Chaerilidae)* [4]; *Sperm duct (part)* [49].
- **Capsular distal carina**: *Lobe interne (Buthidae)* [5]; *Inner process (Buthidae)* [99];* Internal lobe (Buthidae)* [68, 69];* Lateral process (Buthidae)* [64];* Lobulación dorso-externa posterior del lobulió interno* [100];* Inner lobe (Buthidae)* [3];* Median lobe (Buthidae/Chaeriidae)* [4];* Lóbulo interno (Buthidae)* [84].
- **Capsular basal carinae**: *Lobe externe (Buthidae)* [5]; *Superior outer process (Buthidae)* [99]; *External lobe (Buthidae)* [68, 69]; *Median process (Buthidae)* [64]; *Median lobe (Buthidae)* [3]; *Basal lobe (Buthidae) *[3: Fig. 99]; *lateral lobe* [90].
- **Basal hook** (Buthidae): *Lobe basal* [5]; *Oblique vertical process* [99];* Basal lobe* [4, 68, 69]; *Oblique process* [64]; *Basal lobe (Buthidae)* [3];* Median lobe (Buthidae)* [3: Fig. 99]; *Lóbulo basal* [84]; *Process (Pseudochatidae)* [42].
- **Capsular foramen** (spermatophore): Opening of the sperm duct through which the semen is expelled. *Paired sperm exits (Buthidae)* [64]; *Foseta* [77]; *Foramen o ducto espermático (Buthidae)* [84]; *Foramen* [85, 86].
- **Subex** (Latin word meaning basal layer, support): Basal surface of the sperm duct. *Escodatura del lóbulo interno* [9]; *floor of the sperm duct* [4]; *Basal lobe* [98]; *Basal lobe* [41 (Figs. 7, 34, 35), 49].
- **Tectum** (Latin word meaning roof): Distal surface of the sperm duct. *Lobe médian* [5]; *Lóbulo interno* [6, 7, 9, 76, 77, 100];* Median lobe* [3]; *Lobe interne* [103]; *accessory distal lobe* [101]; *Hoja capsular externa* [85]; *External capsular sheet* [86]; *Posterior lobe* [41, 49, 98]; *Internal lobe* [102].
**Subex + tectum**: *Lobe médian (Buthidae)* [5];* Inferior outer process (Buthidae)* [99];* Medial lobe (Buthidae)* [68];* Lateral lobe* [69];* Membrane* [90]; *Trough* [30].
- **Terminal membrane of sperm duct**: Membrane that surrounds the foramen, probably always eversible and intromittent when present, prevents sperm backflow during insemination. *Pórcion basal* [9]; *Membrane* [103]; *Hoja capsular interna* [85]; *Internal capsular sheet* [86]; *Sperm duct* [90]; *Median lobe* [30].
- **Physema** (Greek word meaning something inflated, puffed up, bubble): Externally inflated membranous pouch observed on post-insemination spermatophores of bothriurids and of several chactoid taxa, and formed by the eversion of the capsular terminal membranes.
- **Capsular concavity**: *Lóbulo externo* [6];* Lóbulo externo* [9];* Concavidad capsular* [85];* Capsular concavity* [86, 102].
- **Hemisolenos** (from the Greek words hemi and solen which respectively mean half and pipe/channel): *Lobe basal* [5]; *Valve* [62, 63]; *Inner lobe* [3]; *Internal lobe* [101]; *internobasal reflection of sperm duct* [4]; *Lamella* [41, 49, 98].
- **Holosolenos** (from the Greek words holos and solen which respectively mean whole/entire and pipe/channel): Pipe-like structure on the spermatophore capsule composed of the two hemisolenos and through which the semen is transferred into the female genital tract.
- **Accessory apophysis (of hemisolenos)**: *Accessory lobe* [41];* Lamellar accessory lobe* [49].
- **Accessory hook (of hemisolenos)**: *Lamellar accessory hook* [49].
- **Clasper**: Sclerotized intromittent apophysis that widens the female genital tract and provides a secure anchoring for the spermatophore. *Lobe externe* [5]; *Sacculus* [63]; *Lóbulo basal* [6, 7, 9, 18, 76, 77]; *Lobulación basal* [100]; *Basal lobe* [3, 101]; *Lobe basal* [103]; *Lóbulo capsular* [85];* Capsular lobe* [86]; *Crown-like structure* [90]; *Distal lobe* [41, 49, 98]; *Basal lobe of capsule* [102]; *Ental lobe* [30].
- **Mating plug** [4].
**Distal barb of the mating plug** [4].
- **Basal plate of the mating plug** [4].

**Stem**: Basal part composed of soft membranes and containing the spermatozoa. *Stem* [63, 64]; *Basal tube* [68]; *Porción basal* [6, 7, 9, 18, 76, 77, 100]; *Basal portion* [3, 102];* Trunk* [4, 30, 42, 83, 86, 90]; *Basal part* [101];* Tronco* [84, 85]; *Basal trunk* [49].

- **Truncal flexure**: *Sillon articulaire* [5]; *Repliegue basal* [7, 18, 76]; *Escotadura basal* [9, 77, 100]; *Median transverse cleavage* [3]; *Truncal flexure* [4, 49, 83, 86, 90]; *Articular suture* [101]; *Flexión capsular* [85]; *Basal fold* [102].
- **Pedicel**: Enlarged sticky base that fixes the spermatophore to the substrate. *Anterior process* [99];* Wings* [62]; *Pedal wings* [63];* Anchor piece* [68, 69];* Basal plate* [64];* Lengüeta/ pie ovoidal largo* [7];* Pie* [9, 18, 76, 77, 100];* Foot + Stalk* [3];* Pedicel* [49, 83, 86];* Cylindrical gland* [101];* Pedicelo* [84, 85];* Foot* [42, 90].

### Ancestral state reconstructions

The five bauplans identified during the course of this study were optimized onto the currently available phylogenetic trees of the order Scorpiones proposed by Stockwell [4], Coddington et al. [104], Soleglad & Fet [46],/ Soleglad et al. [105], and Sharma et al. [106] under the parsimony criterion with Mesquite version 2.75 [107]. The presence/absence of invagination on the basal edge of capsule was also optimized onto the phylogenies of Stockwell [4], Prendini [45], and Sharma et al. [106] using the same method.

Character matrices were produced for each of the cladograms (Additional files 1, 2, 3, 4, 5). The different bauplans seems to reflect a gradual evolutionary transition from a simple plain sperm duct towards more complex evertible capsules by successive foldings. The invagination of the capsular basal edge represents the ultimate step of this process eventally resulting in the establishment of the more complex pattern. For these reasons, both characters were assigned linear transformation series and treated as additive (ordered) [108]. The consistency index and retention index of each character were calculated on each of the cladograms in order to evaluate their levels of homoplasy on the different trees.

## Results

### Sampling

**Fig. 3:**
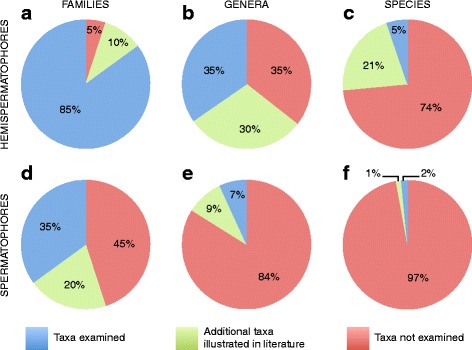
Pie charts showing data coverage of the present study for hemispermatophores (**a**–**c**) and spermatophores (**d**–**f**). **a**, **d** Pie charts showing the proportion of families for which data were obtained from examination of specimens or from the literature during this study, and the proportion of taxa for which data was missing. **b**, **e** Pie charts showing the proportion of genera for which data were obtained from examination of specimens or from the literature during this study, and the proportion of taxa for which data was missing. **c**, **f** Pie charts showing the proportion of species for which data were obtained from examination of specimens or from the literature during this study, and the proportion of taxa for which data was missing

Hemispermatophores of 122 species (5% of known species) belonging to 71 genera (35% of described genera) and 17 families (85% of known families) were studied (Fig. 3a–c), incorporating material from various museum collections [see Additional file 6 for a complete list of specimens]. The relevant literature was thoroughly checked to inventory all published illustrations of hemispermatophores and spermatophores. Illustrations from published taxonomic descriptions and morphological studies were used to complement specimen examination, allowing us to confirm the constancy of defined patterns within taxonomic groups for which only a limited number of taxa had previously been examined. Although we tried to be as exhaustive as possible, some illustrations may have been missed. Photographs or drawings of hemispermatophores and/or spermatophores of 578 species (24% of known species), belonging to 119 genera (58%) and 19 families (100% of known families when omitting the family Akravidae Levy, 2007 which is only known from hollowed-out exoskeletons and probably extinct [109, 110]) were found in the literature [see Additional file 7 for the complete list and Additional file 8 for references].

The combined dataset (data from examined material and from the literature) represent 619 species (26% of known species), 132 genera (65%) and 19 families (100%) (Fig. 3a–c). The hemispermatophore bauplan for each of these taxa was coded [see Additional file 9 for the complete matrix]. In the case of hemispermatophores for which no specimen was available, the vast majority of published illustrations are good enough to assess similarity with the material examined, and, in most cases, to unambiguously assign hemispermatophores to one of the recognized patterns, even if the capsular region was not accurately depicted.

For some taxa no material or only incomplete hemispermatophores were examined, and in a few case the bauplan remains difficult to determine with certainty based on published illustrations alone, i.e. the genus *Lisposoma* Lawrence, 1928, and the families Pseudochactidae and Superstitioniidae. Hemispermatphores of these taxa were nonetheless assigned to a recognised pattern, and the reasons for each decision are given (see relevant paragraphs for detail), pending the study of specimens to confirm the proposed hypotheses.

In addition to the hemispermatophores, 119 spermatophores from 36 species (1% of known species) belonging to 14 genera (7%) and 7 families (35%) (Fig. 3d–f) [See Additional file 10 for the complete material list] were obtained and studied in the course of this study. This structure was previously unknown for 30 of these species, for 9 of these genera, for two of the families and for one of the sub-families [see Additional file 10]. Our dataset does not generally overlap with material from earlier publications and thus represents major progress in our knowledge of scorpion spermatophores, presenting information about more taxa than in all previous publications combined (spermatophores of 34 species belonging to 13 genera and 9 families). The combined data represent 66 species (3% of known species) belonging to 33 genera (16%) and 11 families (55%) (Fig. 3d–f). Moreover, 22 of the spermatophores obtained are from Hormuridae and Scorpionidae, two families for which very little data was previously available in the literature. These spermatophores are among the most complex and they provide essential data for a thorough comparison with the better-known spermatophores of the families Bothriuridae, Buthidae and Euscorpiidae.

### Architecture of the capsule

The primary function of spermatophores is to ensure insemination. The mechanisms by which this is achieved involve two sets of characters: (1) characters pertaining to the overall architecture, which are responsible for the ejection of semen from the spermatophore, and (2) characters of the capsule, which ensure the transfer of semen into the female genital tract. In all scorpion taxa, semen expulsion is carried out by the same mechanism, i.e. an increase of pressure in the internal cavity of the spermatophore induced by a bending of the whole structure [2, 63, 64, 84, 90]. As a result of this conserved mechanism, the general groundplan of the spermatophore with four distinct parts remains the same across the order despite a considerable morphological diversity (Fig. 1).

**Fig. 4:**
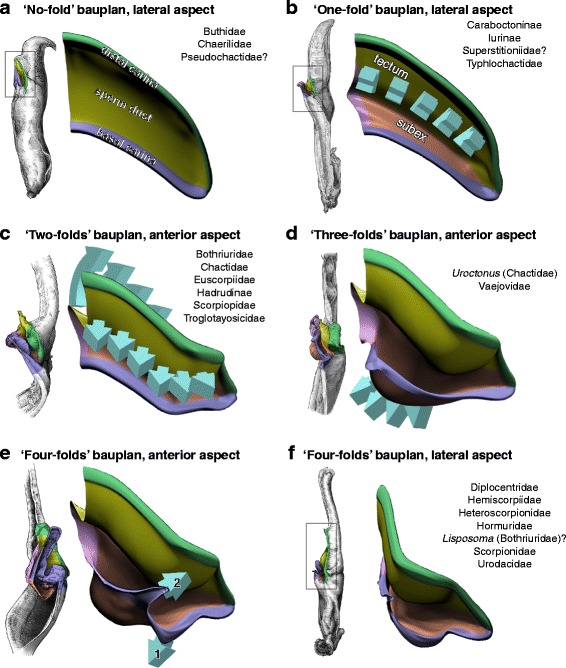
Bauplans and folding processes of the hemispermatophore capsule, diagrammatic representations complemented by camera-lucida drawings with indication of the corresponding area. **a**, ‘No-fold’ pattern, lateral aspect. **b**, ‘One-fold’ pattern, arrows indicate the direction of the folding delimiting the subex and tectum, lateral aspect. **c**, ‘Two-folds’ pattern, anterior aspect. **d**, ‘Three-folds’ pattern observed in *Uroctonus mordax* Thorell, 1876, anterior aspect, arrows show the invagination of the tectum. **e**–**f**, ‘Four-folds’ pattern, anterior (**e**) and lateral (**f**) aspects, arrow 1 shows the invagination of the basal capsular edge and arrow 2 indicates the extension of the hemisolenos

The capsule, that ensures the insemination per se, shows an even greater structural variation, but its architecture follows a linear evolutionary pathway (see discussion) probably due to underlying mechanical constraints. As for the overall groundplan, while proportions and sizes of the various capsular features may differ significantly, the variation of the structural pattern is actually quite limited, mainly consisting of a gradual complexification of the invaginations and foldings of the sperm duct. Five basic bauplans (Fig. 4), accounting for the structural changes of the capsule observed in the examined material, were identified. Each of these bauplans roughly corresponds to an additional fold of the sperm duct. These are described below from the simplest to the most complicated architecture. The term ‘bauplan’ is used here to designate an overall architectural pattern or organization invariant among a wide range of taxa [60].

In most illustrations of the capsule presented, the different parts are color-coded as follows: (1) the distal carina is in green, (2) the basal carina in blue, (3) the sperm duct is given in yellow in the first bauplan, (4) the tectum is in yellow and the subex in orange in the following bauplans, and (5) the terminal membrane is in pink. Corresponding parts in hemispermatophores and spermatophores, and homologous parts in hemispermatophores/spermatophores of different taxa are thus easily identifiable.

#### I. ‘No-fold’ bauplan

**Fig. 5:**
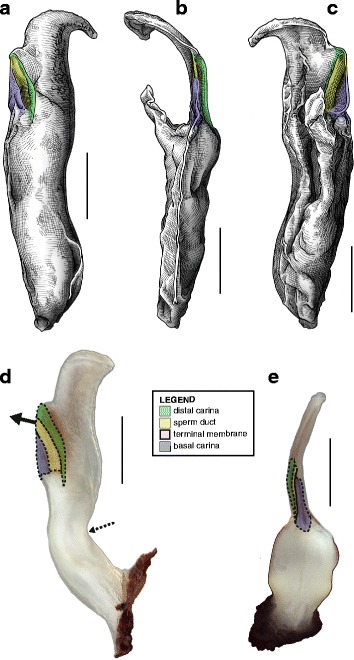
Male reproductive apparatus of Chaerilidae Pocock, 1893. *Chaerilus phami* Lourenço, 2011 (Vietnam, Conson Island, MHNG). Hemispermatophore (**a**–**c**), lateral (**a**), anterior (**b**) and contra-lateral (**c**) aspects. Post-insemination spermatophore (**d**–**e**), lateral (**d**) and anterior (**e**) aspects. The full arrow indicates the site and direction of semen expulsion and the dotted arrow indicates the bend of the stem responsible for the increase of pressure inside the sperm reservoir. Scales, 0.5 mm

**Fig. 6:**
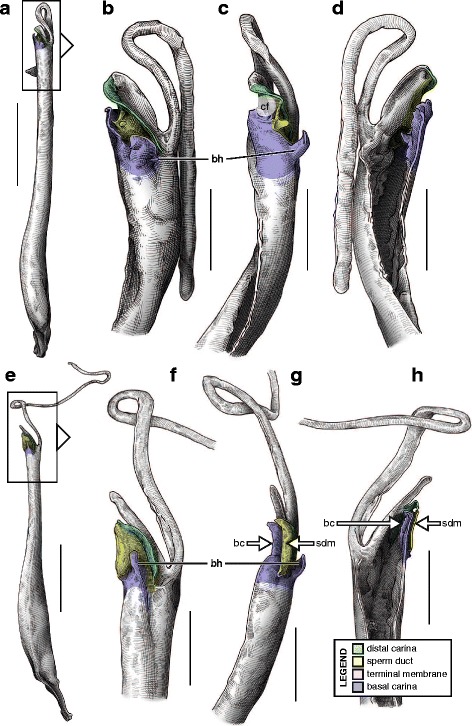
Hemispermatophores of Buthidae Koch, 1837. *Babycurus buettneri* Karsch, 1886 (Cameroon, Ebogo, MHNG) (**a**–**d**) and *Hottentotta jayakari* (Pocock, 1895) (United Arab Emirates, Wadi Wurrayah, MHNG) (**e**–**h**). Lateral (**a**, **b**, **e**, **f**), anterior (**c**, **g**) and contra-lateral (**d**, **h**) aspects. Abbreviations: bc (basal carina), bh (basal hook), cf (capsular foramen), sdm (sperm duct membrane). Arrows show the sperm duct membrane overlapping the basal carina. Scales, 2 mm (**a**), 0.5 mm (**b**–**d**), 3 mm (**e**), 1 mm (**f**–**h**)

This is the simplest pattern observed, with a non-folded sperm duct (Fig. 4a). This is present in the families Buthidae, Chaerilidae (Figs. 5–6), and putatively also in the Pseudochactidae.

The monogeneric family Chaerilidae possess hemispermatophores with the simplest capsule, consisting of two carinae surrounding an unfolded *sperm duct* ([4]; Fig. 5a–c yellow). Although there is no visible foramen, the semen can nevertheless be expelled because the membranes of the two hemispermatophore are not fixed together in the spermatophore between the capsular ridges (arrow in Fig. 5d). The same basic “no-fold” pattern is observed in the family Buthidae ([3, 4]; Fig. 6). However, in contrast to chaerilid hemispermatophores, a foramen (cf in Fig. 6c and g) is clearly visible in buthid hemispermatophores, because the duct membrane is shifted away from the longitudinal axis as a result of wider capsular carinae. In some other buthid taxa, like observed here in *Hottentotta jayakari* (Pocock, 1895), the duct membrane even extends above the basal carina (*sdm* in Fig. 6g–h yellow). The insemination process is similar in the Chaerilidae (this publication) and Buthidae [64, 65, 68–72, 75, 78, 80–84, 88], without alteration of the structure of the capsule. Furthermore, unlike other spermatophores, buthid and chaerilid spermatophores do not have a fixed truncal flexure; the stem is simply bent backward during mating, medially in chaerilids (dotted arrow in Fig. 5d) and more basally in the elongated buthid spermatophores.

According to illustrations in Prendini et al. [42], the hemispermatophore of Pseudochactidae appears to be morphologically intermediate between those of buthids and chaerilids. The stem is short as in chaerilids, and the stalk is flagelliform but much thicker than that observed in buthids. The capsule is not shown in detail in Prendini et al. [42], therefore the bauplan could not be determined and potential similarities with buthid and chaerilid capsules could not be assessed accurately. An apophysis at the base the stalk was interpreted by Prendini et al. [42] to be homologous with the basal hook of the basal carinae of the buthid hemispermatophores (*bh* in Fig. 6), but this cannot be unambiguously confirmed at the moment. Therefore, solely based on gross morphology, pseudochactid hemispermatophores are putatively considered here to possess the same bauplan as buthids and chaerilids, although this needs to be verified.

#### II. ‘One-fold’ bauplan

The sperm duct membrane folds itself longitudinally, forming two perpendicular surfaces (Fig. 4b) that are designated here according to their positions: the basal surface is named subex (Fig. 4b orange) and the more distal plane, tectum (Fig. 4b yellow).

**Fig. 7:**
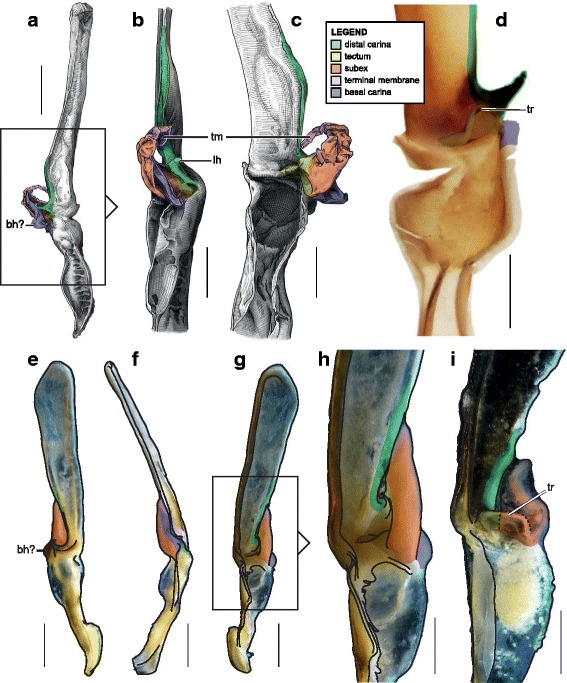
Hemispermatophores of Iuridae Thorell, 1876 (**a** *–* **d**) and Typhlochactidae Mitchell, 1971 (**e** *–* **i**). *Iurus kinzelbachi* Kovařík, Fet, Soleglad & Yağmur, 2010 (Greece, Samos, MHNG) (**a**–**d**) and *Alacran triquimera* Santibáñez-López, Francke & Prendini, 2014 (México, Cueva de las Tres Quimera*,* CNAN) (**e** *–* **i**). Lateral (**a**, **e**), anterior (**b**, **f**) and contra-lateral (**c**–**d**, **g**–**i**) aspects. The protruding part of the sperm duct has been teared off in **d**, showing the transverse ridge that is usually hidden behind it. The transverse ridge is also apparent in **i** behind the transparent subex. Abbreviations: bh (basal hook), lh (lateral hook), tm (terminal membrane), tr (transverse ridge). Scales, 2 mm (**a**), 1 mm (**b**–**g**), 0.5 mm (**d**, **h**–**i**)

The sperm duct of the hemispermatophore capsule in the family Iuridae (Fig. 7) show significant modifications of the capsule proportions compared to the pattern observed in buthids/chaerilids/pseudochactids. The capsule is extended anteriorly, protruding conspicuously. This membranous process is mostly composed of the subex (Fig. 7 orange) and is terminated by a thin membrane (*tm* in Fig. 7 pink) where the foramen is usually located. A broad apophysis is located at the base of the basal carina (*bh* in Fig. 7a) and may be homologous with the basal hook of Buthidae. Hemispermatophore morphology is extremely conservative in the Iuridae, with only slight differences between species and even genera [29, 111–116].

The capsular region of hemispermatophores in the Typhlochactidae (Fig. 7e–i; [34, 117]) is strikingly similar to that of iurids: the capsule is markedly elongated anteriorly into a membranous process. Moreover, the transverse ridge is curved towards and very close to the lateral hook (*tr* in Fig. 7i). For these reasons typhlochactid hemispermatophores are assigned to the ‘one-fold’ bauplan. Hemispermatophores of the family Superstitioniidae are also putatively assigned to the ‘one-fold’ bauplan on the basis of illustrations from Francke [34]. As first mentioned by Francke & Soleglad [29], superstitionid hemispermatophores possess an extended, weakly sclerotized sperm duct similar to that of iurids.

**Fig. 8:**
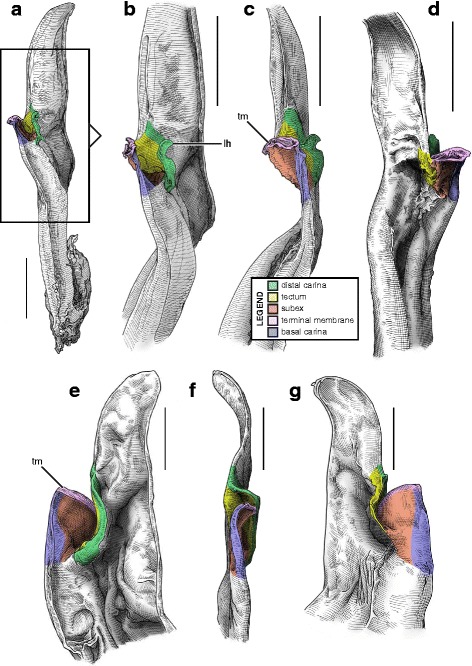
Hemispermatophores of Caraboctoninae Kraepelin, 1905 (Caraboctonidae Kraepelin, 1905). *Caraboctonus keyserlingi*, Pocock, 1893 (Chile, MHNG) (**a**–**d**) and *Hadruroides mauryi* Francke & Soleglad, 1980 (Peru, Huanta, FKPC) (**e**–**g**). Lateral (**a**, **b**, **e**), anterior (**c**, **f**) and contra-lateral (**d**, **g**) aspects. Abbreviations: cf (capsular foramen), lh (lateral hook), tm (terminal membrane). Scales, 1 mm (**a**–**d**), 0.5 mm (**e**–**g**)

A deep longitudinal folding clearly divides the sperm duct into two surfaces, subex and tectum (Fig. 8 orange and yellow respectively) in hemispermatophores of the caraboctonin genera *Caraboctonus* Pocock, 1893 ([4, 29]; Fig. 8a–d) and *Hadruroides* Pocock, 1893 ([4, 29]; Fig. 8e–g). The subex (Fig. 8e–g orange) remains in the same axis as the stalk in *Hadruroides*, whereas in *Caraboctonus* it is rotated outward, away from the axis of the stalk, by about 90° (Fig. 8a–d orange). The terminal membrane observed in *Iurus* is also present in these taxa but remains small (*tm* in Fig. 8 pink). The hemispermatophores of *Caraboctonus* and *Hadruroides* were first illustrated in Francke & Soleglad [29] and Stockwell [4]. Ochoa & Prendini [118] later presented a revision of the Peruvian *Hadruroides* species, with illustrations of spermatophores for six of these taxa showing that the structure is extremely conservative within the genus.

The ‘one-fold’ bauplan is the only one for which the insemination mechanisms are still unclear. Although the spermatophores of Iuridae and Caraboctoninae Kraepelin, 1905 remain unknown, the post-insemination spermatophore of *Superstitionia donensis* Stahnke, 1940 was illustrated in Francke [83]. It shows the absence of protruding features from the capsular region, suggesting that insemination probably occurs without the evertion of internal structures as in buthids and chaerilids. The morphology of the hemispermatophores also suggests that eversion of the capsule probably does not occur: there is no weak spot that could potentially serve as a rotation point (the subex and tectum are held in position by the sclerotized part of the structure). If eversion occurs, (1) it most certainly remains limited to the short terminal membrane, and (2) there is probably no intromission of this membrane into the female genital tract because it is too soft. The terminal membrane may nonetheless limit semen loss during mating by covering the sides of the gonopore. Obtaining spermatophores is necessary to test these hypotheses.

#### III. ‘Two-folds’ bauplan

**Fig. 9:**
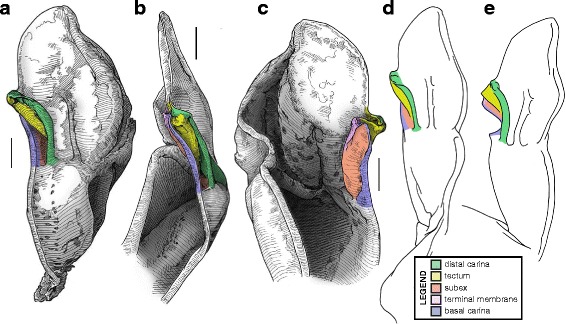
Male reproductive apparatus of Hadrurinae Stahnke, 1973 (Caraboctonidae Kraepelin, 1905). Hemispermatophore of *Hadrurus* sp. (USA, MHNG) (**a**–**c**). Spermatophore of *Hadrurus arizonensis* Ewing, 1928 (USA, Arizona, Maricopa County), pre- (**d**) and post-insemination (**e**), redrawn from Francke (1989). Lateral (**a**, **d**, **e**), anterior (**b**) and contra-lateral (**c**) aspects. Scale, 1 mm (**a**–**c**)

**Fig. 10:**
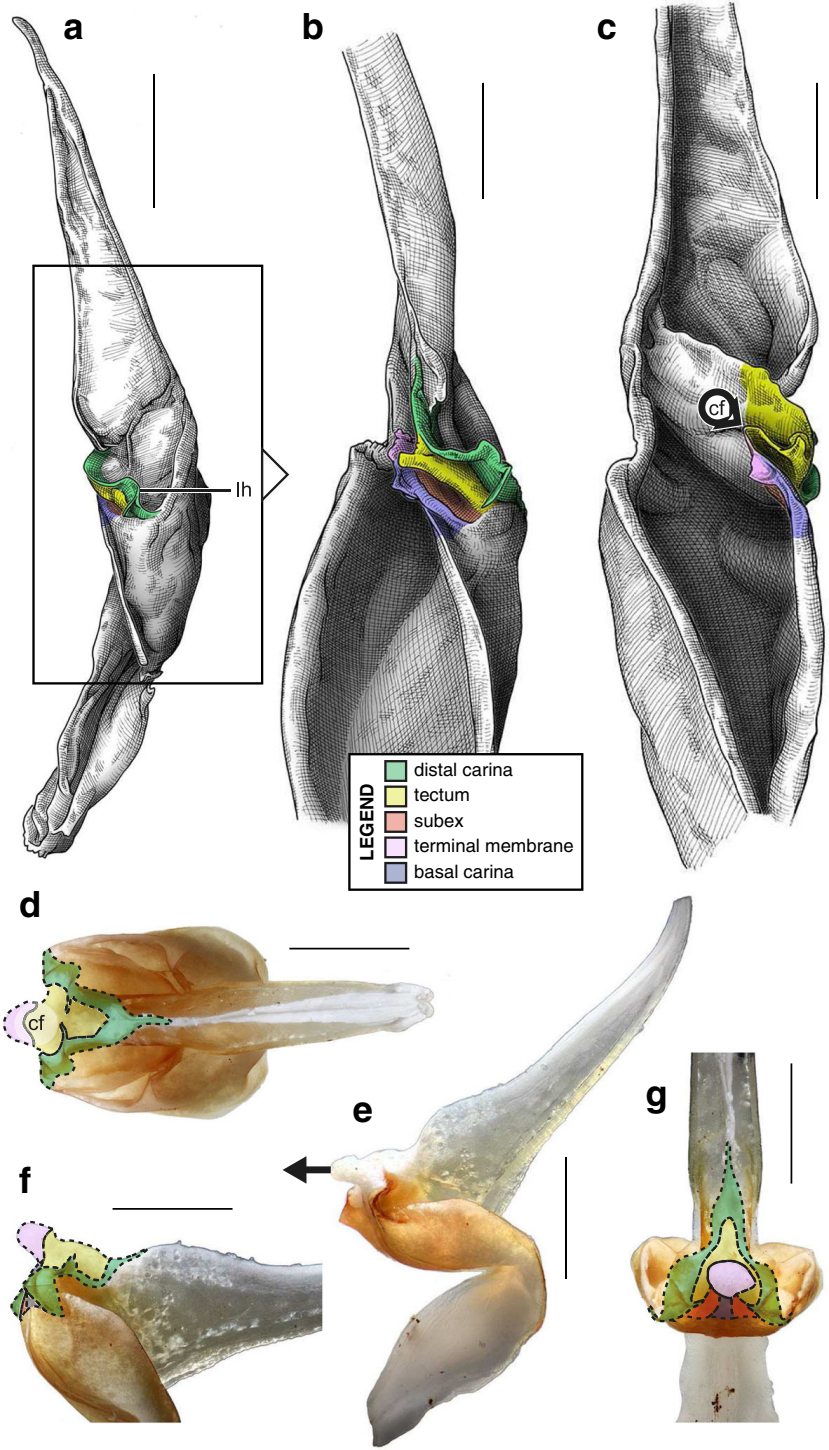
Male reproductive apparatus of Uroctoninae Mello-Leitão, 1934 (Chactidae Pocock, 1893). Hemispermatophore of *Anuroctonus* cf. *phaiodactylus* (Wood, 1863) (USA, FKPC) (**a**–**c**). Post-insemination spermatophore of *Anuroctonus* pococki Soleglad & Fet, 2004 (USA, Riverside County, MHNG) (**d**–**g**). Lateral (**a**, **e**, **f**), anterior (**b**, **g**), contra-lateral (**c**), and dorsal (**d**) aspects. The full arrow indicates the site and direction of semen expulsion. Abbreviations: cf (capsular foramen), lh (lateral hook). Scales, 2 mm (**a**, **d**–**g**), 1 mm (**b**–**c**)

**Fig. 11:**
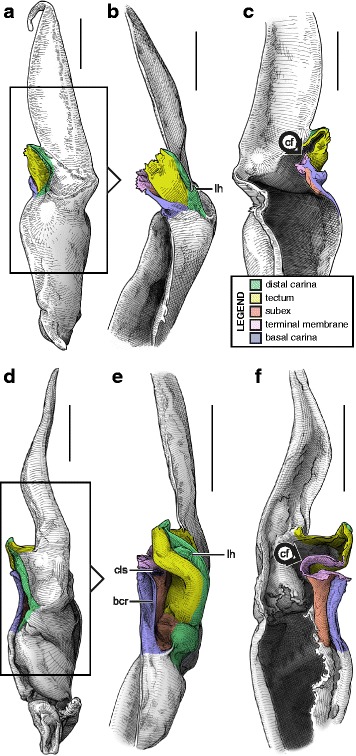
Hemispermatophores of Troglotayosicidae Lourenço, 1998 (**a**–**c**) and Chactinae Pocock, 1893 (Chactidae Pocock, 1893) (**d**–**f**). *Belisarius xambeui* Simon, 1879 (France, Pyrénées Orientales, MHNG) (**a**–**c**) and *Chactas* sp. (Colombia, MHNG) (**d**–**f**). Lateral (**a**, **d**), anterior (**b**, **e**) and contra-lateral (**c**, **f**) aspects. Abbreviations: bcr (basal crest), cf (capsular foramen), cls (crown-like structure), lh (lateral hook). Scales, 0.5 mm (**a–c**), 1 mm (**d–f**)

**Fig. 12:**
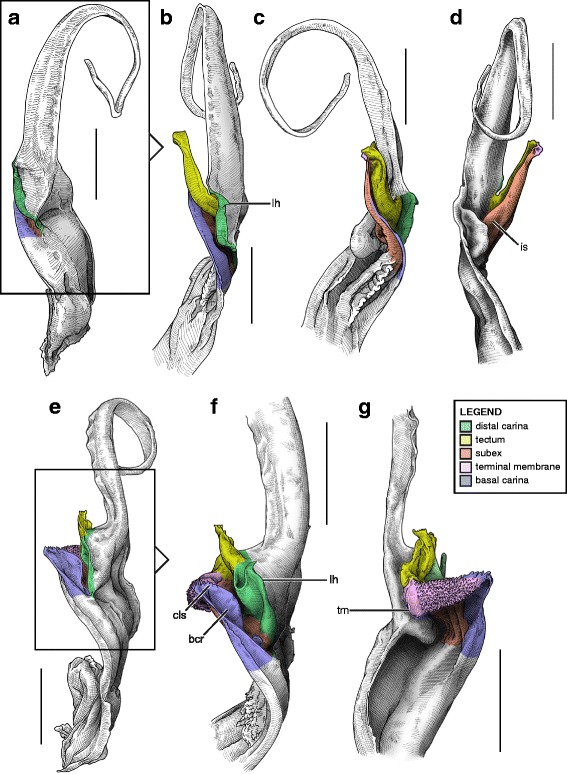
Hemispermatophores of Brotheinae Simon, 1879 (Chactidae Pocock, 1893) and Scorpiopidae Kraepelin, 1905. *Brotheas gervaisii* Pocock, 1893 (Brazil, Amapa, MHNG) and *Scorpiops* sp. (Myanmar, MHNG). Lateral (**a, e**), anterior (**b–c, f**) and contra-lateral (**d, g**) aspects. Abbreviations: bcr (basal crest), cf (capsular foramen), cls (crown-like structure), is (invagination of the subex), lh (lateral hook), tm (terminal membrane). Scales, 1 mm

**Fig. 13:**
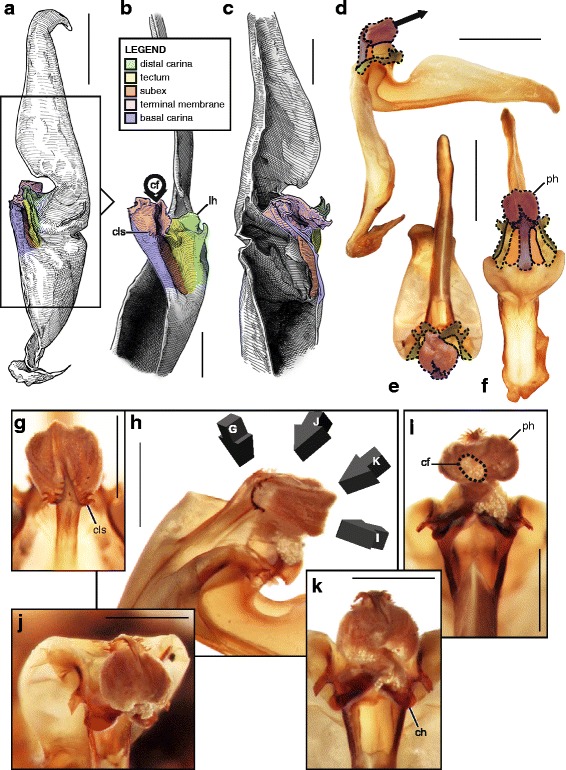
Male reproductive apparatus of the genus *Euscorpius* Thorell, 1876. Hemispermatophore of *Euscorpius* cf. *carpathicus* (Linnaeus, 1767) (Croatia, MHNG) (**a–c**). Post-insemination spermatophore of *Euscorpius italicus* (Herbst, 1800) (Switzerland, Wallis, MHNG), in toto habitus (**d–f**) and details of the capsular region showing the inflated physema (**g–k**). Lateral (**a, d, h**), anterior (**b, f**), contra-lateral (**c**), and dorsal (**e**) aspects. Abbreviations: cf (capsular foramen), cls (crown-like structure), lh (lateral hook), ph (physema). Scales, 1 mm (**a, g–k**), 2 mm (**d–f**), 0.5 mm (**b–c**)

Here, the tectum is folded longitudinally twice, in opposite directions (Fig. 4c), which results in the complete invagination of the capsule into the interior of the spermatophore. In hemispermatophores (i.e. non-everted state of spermatophore halves), the subex and tectum sit parallel to each other in accordion-pleats (Figs. 9b–c, 10b–c, 11b–c and e–f, 12b–d and f–g, 13b–c orange and yellow respectively). In this bauplan the architecture and the softness of the sperm duct result in reconfiguration during insemination. Semen expulsion from the spermatophore reservoir triggers the unfolding of the sperm duct outward, leading to the protrusion of the capsule from the spermatophore, i.e. the eversion of the capsule.

**Fig. 14:**
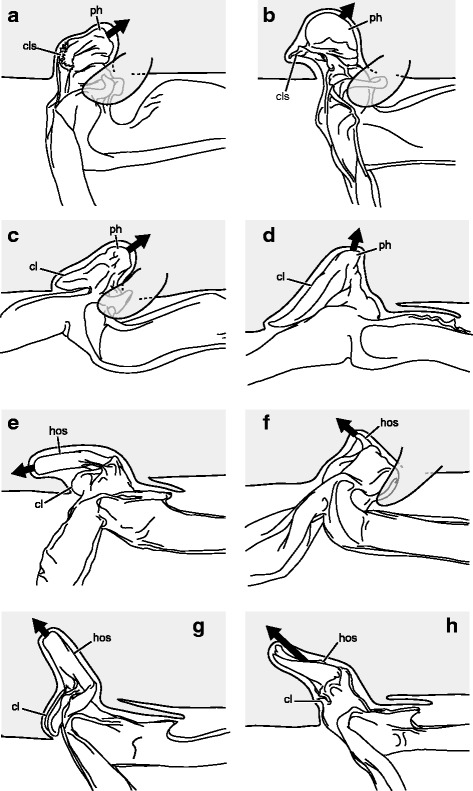
Details of the position of the everted capsule inside the female genital tract in *Euscorpius italicus* (Herbst, 1800) (**a**), *Uroctonus mordax* Thorell, 1876 (redrawn from Stockwell, 1989; **b**), *Bothriurus flavidus* Kraepelin, 1911 (redrawn from Pererri, 2010; **c**), *Bothriurus bonariensis* (C-.L.Koch, 1842) (redrawn from Pererri, 2010; **d**), *Hormiops davidovi* Fage, 1934 (**e**), *Nebo* cf. *whitei* Vachon, 1980 (**f**), *Heterometrus mysorensis* Kovařík, 2004 (**g**) and *Chiromachus ochropus* (Koch, 1937) (**h**). Abbreviations: cl (clasper), cls (crown-like structure), hos (holosolenos), ph (physema). Full arrows indicate the sites and directions of semen expulsion

Hemispermatophore capsules in the Chactoidea and Hadrurinae Stahnke, 1973 (Caraboctonidae) (Figs. 9, 10, 11, 12, 13, 14a) are characterized by a ‘two-folds’ bauplan, with the capsule invagination showing different degrees of development. As in other groups, the general pattern is conservative, but the proportions of the different parts of the capsule can be extremely variable. *Anuroctonus* Pocock, 1893 ([4, 29]; Fig. 10), *Belisarius* Simon, 1879 (Fig. 11a–c) and *Hadrurus* Thorell, 1876 ([4, 29]; Fig. 9) possess a capsule with relatively limited internal folding and thus with a relatively short protruding sperm duct after eversion (Figs. 9d–e, 10d–g). On the other hand, in Brotheinae Simon, 1879 ([4]; Fig. 12a–d), Chactinae Pocock, 1893 ([4]; Fig. 11d–f), Euscorpiidae Laurie, 1896 ([4, 43, 83, 119, 120]; Fig. 13) and Scorpiopidae ([4, 121]; Fig. 12e–g), the folding pattern is similar, but the invagination is significantly more pronounced, leading to a larger intromittent structure when everted (Fig. 13d–k).

Moreover, in the genera *Brotheas* Koch, 1837, *Broteochactas* Pocock, 1893, *Hadrurochactas* Pocock, 1893, *Neochactas* Soleglad & Fet, 2003 and *Teuthraustes* Simon, 1878, the capsule ([4]; Fig. 12a–d) is radically modified in comparison with capsules observed in other Chactoidea. The sperm duct appears to be elongated and twisted inward, with the subex (Fig. 12a–d orange) forming a rather deep invagination (*is* in Fig. 12d). A similar inward twist and elongation of the sperm duct is present in the genera *Chactopsis* Kraepelin, 1912, *Chactopsoides* Ochoa, Rojas-Runjaic, Pinto-da-Rocha & Prendini, 2013 and *Megachactops* Ochoa, Rojas-Runjaic, Pinto-da-Rocha & Prendini, 2013 [30].

Interestingly, scorpiopid hemispermatophores ([4, 121]; Fig. 12e–g) present a morphology intermediate between the chactid genera mentioned in the previous paragraph and other Chactoidea, i.e. the tectum is extended and twisted inward as in the brotheins, but the rest of the capsule remains similar to the basic chactoid morphology. Furthermore, hemispermatophores of *Chactopsis*, *Chactopsoides* and *Megachactops* share some morphological similarities with scorpiopids. Unlike *Brotheas*, *Broteochactas*, *Hadrurochactas, Neochactas* and *Teuthraustes*, they possess an extended terminal membrane covered with spicules, and their subex is not completely folded over itself [30].

A terminal membrane is present in all taxa examined. It is covered with minute spicules in *Chactopsis* [30], Euscorpiidae ([4, 39, 90, 120]; Fig. 13a–f pink and 13 g–k) and Scorpiopidae ([4, 121]; *tm* in Fig. 12g pink). When the eversion of the capsule is triggered, the terminal membrane forms a sort of externally inflated membranous pouch [4, 90], referred to here as the physema (*ph* in Fig. 13d–f pink and g–k), which helps to widen the female genital atrium and to maintain a tight fit between spermatophore and female genital tract, preventing sperm backflow [90].

A clump of spinules on the distal part of the basal carina, the crown-like structures (sensu Jacob et al. [90]), was first reported in *Euscorpius* Thorell, 1876 ([4, 90]; *cls* in Fig. 13) and *Megacormus* (Karsch, 1881) [4, 119, 120]. This feature is also present in *Chactas* Gervais, 1844 (*cls* in Fig. 11e) and Scorpiopidae (*cls* in Fig. 12f), but in these taxa, it is more reduced and basally extended by a membranous crest that runs along the basal carina (bcr in Figs. 11d–e and 12e–f).

**Fig. 15:**
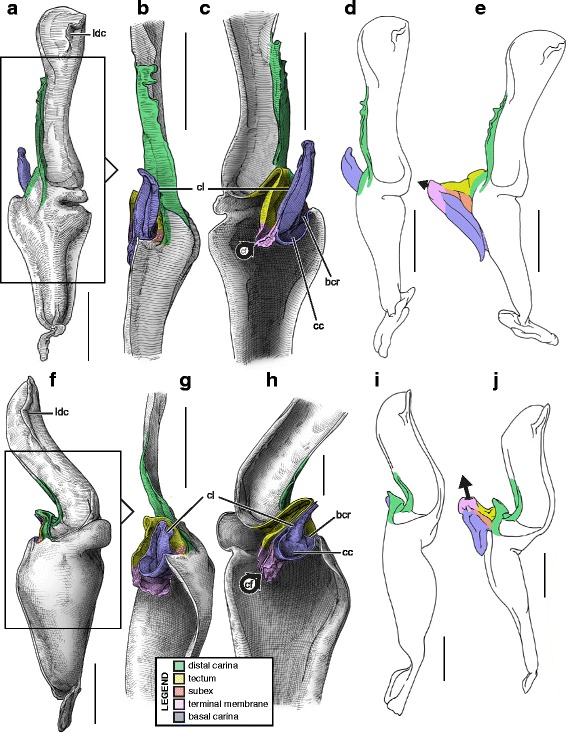
Male reproductive apparatus of Bothriudiae Simon, 1880**.** Hemispermatophore (**a** ***–*** **c**), pre-(**d**) and post-insemination (**e**) spermatophore of *Bothriurus bonariensis* (C-.L.Koch, 1842) (Paraguay, MHNG), spermatophore redrawn from Peretti (2010). Hemispermatophore of *Bothriurus burmeisteri* Kraepelin, 1894 (Argentina, Chubut Province, MHNG) (**f** ***–*** **h**). Pre- (**i**) and post-insemination (**j**) spermatophore of *Bothriurus flavidus* Kraepelin, 1911 (**i–j**), redrawn from Peretti (1995). Lateral (**a, d–f, i–j**), anterior (**b, g**) and contra-lateral (**c, h**) aspects. Abbreviations: bcr (basal crest), cc (capsular concavity), cf (capsular foramen), cl (clapser), ldc (latero-distal crest). Full arrows indicate the sites and directions of semen expulsion. Scales, 2 mm (**a** ***–*** **g**), 1 mm (**h–j**)

**Fig. 16:**
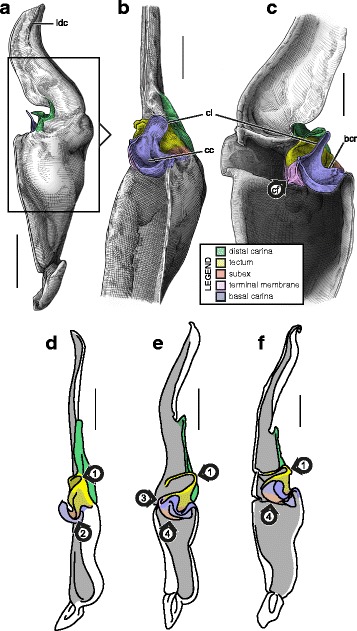
Hemispermatophore of Bothriuridae Simon, 1880. *Cercophonius squama* (Gervais, 1843) (Australia, MHNG) (**a–c**). *Lisposoma elegans* Lawrence, 1928 (**d** ***–*** **e**) and *Lisposoma josehermana* Lamoral, 1979 (**f**), redrawn and adapted from Lamoral [3]. Lateral (**a**), anterior (**b, d**) and contra-lateral (**c, e** ***–*** **f**) aspects. Arrow 1 indicates the anterior protrusion of the subex (= semi-lunar shelf sensu Stockwell [4]). Arrow 2 indicates the absence of capsular concavity and basal crest. Arrow 3 indicates the absence of terminal membrane and capsular foramen. Arrow 4 indicates the invaginated subex. Abbreviations: bcr (basal crest), cf (capsular foramen), cl (clapser), cc (capsular concavity), ldc (latero-distal crest). Scales, 1 mm (**a, f**), 0.5 mm (**b** ***–*** **e**)

Hemispermatophore capsules of Bothriuridae (Figs 14c and d, 15, 16) presumably also belong to the ‘two-folds’ type. Bothriuridae is probably the scorpion family for which hemispermatophores, spermatophores and insemination mechanisms are best known as a result of the extensive work of several generations of Latin American researchers [4, 8–22, 55, 56, 66, 74, 76, 77, 79, 85–87, 89, 92, 122–151]. In this family the capsule folding pattern is quite similar to that of the Chactoidea. The sperm duct is bent on two planes, i.e. subex and tectum (Figs. 15 and 16 orange and yellow respectively), and possess a rather large terminal membrane (Figs. 15 and 16 pink). In everted spermatophores this membrane forms an inflated vesicle, the physema, from which the semen is expelled ([55, 66, 74, 76, 77, 79, 85–87, 89]; Figs. 13c–d, 15e and j pink). A pair of long sclerotized processes, referred to as claspers (*cl* in Figs. 14c–d, 15b–c, g–h, 16b–c), is present on the basal part of the capsule. We consider these to be modifications of the basal carinae. Like the chactid crown-like structures, they are extended anteriorly by wide membranous crests that merge with the antero-basal edge of the sperm duct (*bcr* in Figs. 15c and h, 16b–c), forming a capsular concavity sensu Peretti [85] (*cc* in Figs. 15c and h, 16b–c) basally.

We requested a loan for the paralectotype of the African bothriurid *Lisposoma elegans* Lawrence, 1928 (SAMC B6077) dissected by Lamoral [152] and for which the right hemispermatophore was relatively well illustrated in Lamoral [3] and Prendini [152]. The left hemispermatophore appears to be the only remaining part of this material; the staff could not locate the right half. Its examination revealed that the capsule has been completely torn off, preventing analysis of the capsular structure. Although the published illustrations [3; 152] are not sufficient to determine the bauplan unambiguously, they nevertheless allow the identification of several important differences between the hemispermatophore of *Lisposoma* and those of non-African bothriurids and suggest that the former is morphologically closer to hemispermatophores of non-bothriurid scorpionoids.

Stockwell [4] and Prendini [45, 152, 153] recorded the presence of a “semi-lunar shelf” or “internal cresentic shelf” on the internal wall of the sperm duct invagination in *Lisposoma* (arrow 1 in Fig. 16d–f yellow,), a character they considered synapomorphic for the Bothriuridae. This structure was referred to by previous authors as ‘Lóbulo interno’ (= internal lobe) and corresponds to the tectum. Its occurrence however, is not restricted to the capsular pattern of Bothriuridae; it is present in all hemispermatophores with a two-folds, three-folds and four-folds capsule, but does not protrude anteriorly in all taxa, making it less conspicuous.

The distal lamina of the *Lisposoma* hemispermatophore does not bear the typical latero-distal bothriurid crest [3, 4, 152, 153] and the capsule also seems to be different from the bothriurid pattern. There is no basal capsular concavity and basal crest (arrow 2 in Fig. 16d). The capsular foramen at the base of the tectum is also absent (arrow 3 in Fig. 16e) and the subex is slightly more invaginated than in other bothriurids (arrow 4 in Fig. 16e–f orange). These characters suggest that the sperm duct fold in *Lisposoma* is homologuous to the hemisolenos of non-bothriurid scorpionoids rather than to the bothriurid claspers.

#### IV. ‘Three-folds’ bauplan

**Fig. 17:**
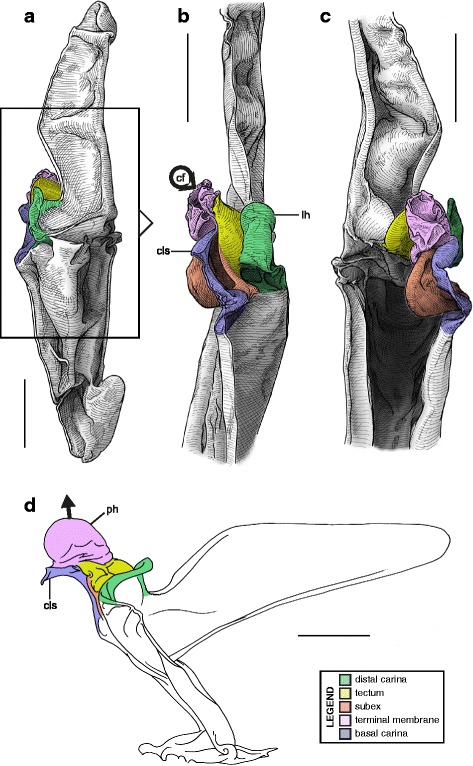
Male reproductive apparatus of Uroctoninae Mello-Leitão, 1934 (Chactidae Pocock, 1893). Hemispermatophore of *Uroctonus mordax* Thorell, 1876 (U.S.A., California, MCZH 15924) (**a** ***–*** **c**). Post-insemination spermatophore of *U. mordax* (**d**), redrawn from Stockwell (1989). Lateral (**a**, **d**), anterior (**b**), and contra-lateral (**c**) aspects. The black arrow indicates the site and direction of semen expulsion. Abbreviations: cf (capsular foramen), cls (crown-like structure), lh (lateral hook), ph (physema). Scales, 1 mm

**Fig. 18:**
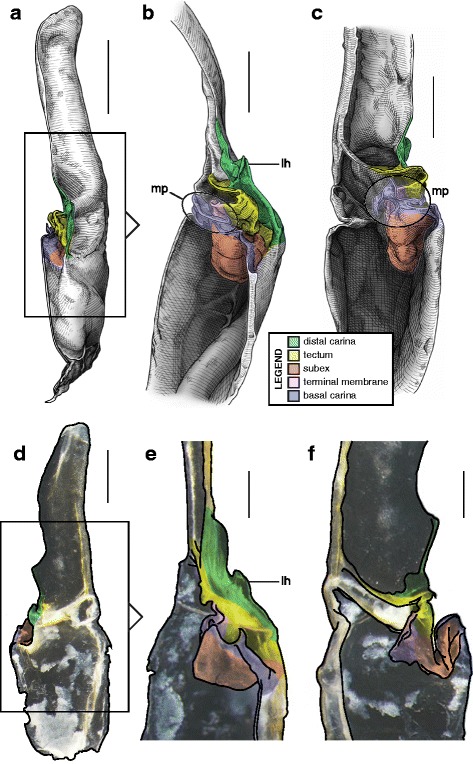
Hemispermatophores of Vaejovidae Thorell, 1876. *Paruroctonus utahensis* Williams, 1968 (U.S.A, New Mexico, MCZH 15845) (**a**–**c**). *Vaejovis dugesi* Pocock, 1902 (México, Sierra de Lobos, ITESI-S046) (**d**–**e**). Lateral (**a**, **d**), anterior (**b**, **e**) and contra-lateral (**c**, **f**) aspects. Abbreviations: lh (lateral hook), mp (mating plug). Scales, 1 mm (**a**), 0.5 (**b**–**d**), 0.25 mm (**e**–**f**)

**Fig. 19:**
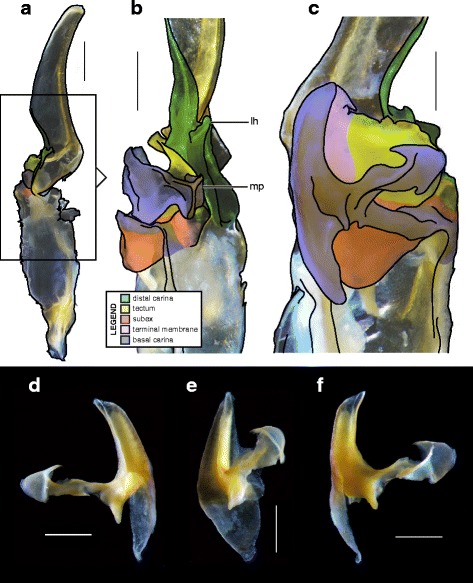
Hemispermatophores of Vaejovidae Thorell, 1876. *Franckeus nitidulus* (C.L.Koch, 1843) (México, Tasquillo, CAFC-S0032), hemispermatophore in toto (**a** ***–*** **b**) and mating plug (**d** ***–*** **e**). Lateral (**a, d**), anterior (**b, e**) and contra-lateral (**c, f**) aspects. Abbreviations: lh (lateral hook), mp (mating plug). Scales, 1 mm (**a**), 0.5 mm (**b–f**)

An additional folding of the sperm duct is observed in this structural type, translating morphologically into a pronounced invagination of the subex (Figs 4d, 17b–c, 18b–c and e–f, 19b–c orange). Three different groups are recognized within this bauplan depending on the presence/absence of accessory appendages, i.e. mating plug and terminal membrane.

The genus *Uroctonus* Thorell, 1876 has very peculiar hemispermatophores with characters specific to Chactoidea and Vaejovoidea. In addition to the deeply invaginated subex (Fig. 17b–c orange), the capsule possesses a rather large terminal membrane (Fig. 17b–c pink) that forms a physema in everted spermatophores ([4]; Figs. 14b, 17d pink), as in the euscorpiins (Fig. 13d–k). The basal carina only bears a small process, which is deemed homologous to the crown-like structure of the euscorpiids (*cls* in Fig. 17b–d).

In several taxa of the family Vaejovidae, e.g. the genus *Smeringurus* Haradon, 1983, *Uroctonites huachuca* (Gertch & Soleglad, 1972), and some *Vaejovis* species mostly belonging to the *mexicanus-*group [154–160] (see Additional file 9), the capsule only has the invaginated subex (Fig. 18d–f orange) and does not bear any accessory appendages, terminal membrane or mating plug (Fig. 18d–f).

Hemispermatophores in the rest of the family Vaejovidae do not have the terminal membrane that is present in *Uroctonus*, instead semen is transferred into the female genital tract through a sclerotized mating plug attached to the basal capsular carina (*mp* in Figs. 18b–c, 19b–f). The mating plug is everted into the female genital tract during mating. It is only tenuously attached to the carina, and once inserted into the female genital atrium, it is easily detached, thereby sealing the gonopore after insemination [4, 161–164], probably to prevent other males from mating with that female [161]. Although the mating plugs show significant variation in size and shape across the family [4, 31, 32, 48, 162–175], the general architecture is the same, i.e. an enlarged ‘basal piece’ (sensu Stockwell [4]) from which protrudes an elongated apophysis ending in a ‘distal barb’ that may bear distal hooks.

#### V. ‘Four-folds’ bauplan

This structural pattern (Fig. 4e–f), characterised by a sperm duct presenting four major folds is only observed in the hemispermatophores of non-bothriurid Scorpionoidea Latreille, 1802 (Figs. 14e–h, 20, 21, 22, 23, 24, 25, 26, 27, 28, 29, 30, 31, 32, 33). In addition to the three folds in the previous bauplan, a medial invagination of the basal part of the capsule opposite the deep subex (arrow 1 in Fig. 4e–f) leads to the narrowing of the basal edge into a pipe-like structure (arrow 2 in Fig. 4e–f).

**Fig. 20:**
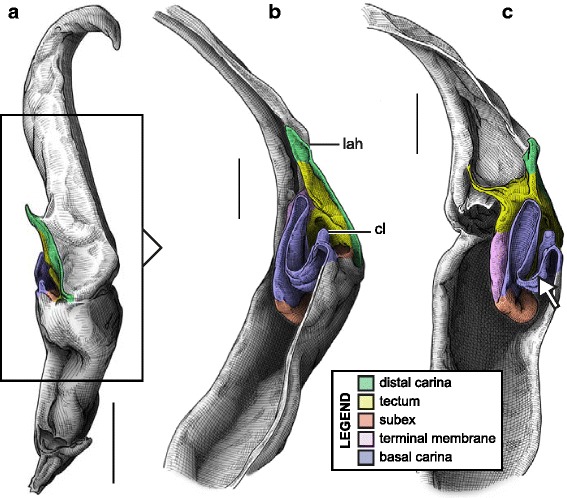
Hemispermatophores of Heteroscorpionidae Kraepelin, 1905. *Heteroscorpion opisthacanthoides* (Kraepelin, 1896) (Madagascar, Nosy Be, MHN). Lateral (**a**), anterior (**b**) and contra-lateral (**c**) aspects. The white arrow shows the invagination of the basal edge of the capsule. Abbreviations: cl (clasper), lah (laminar hook). Scales, 2 mm, (**a**), 1 mm (**b–c**)

**Fig. 21:**
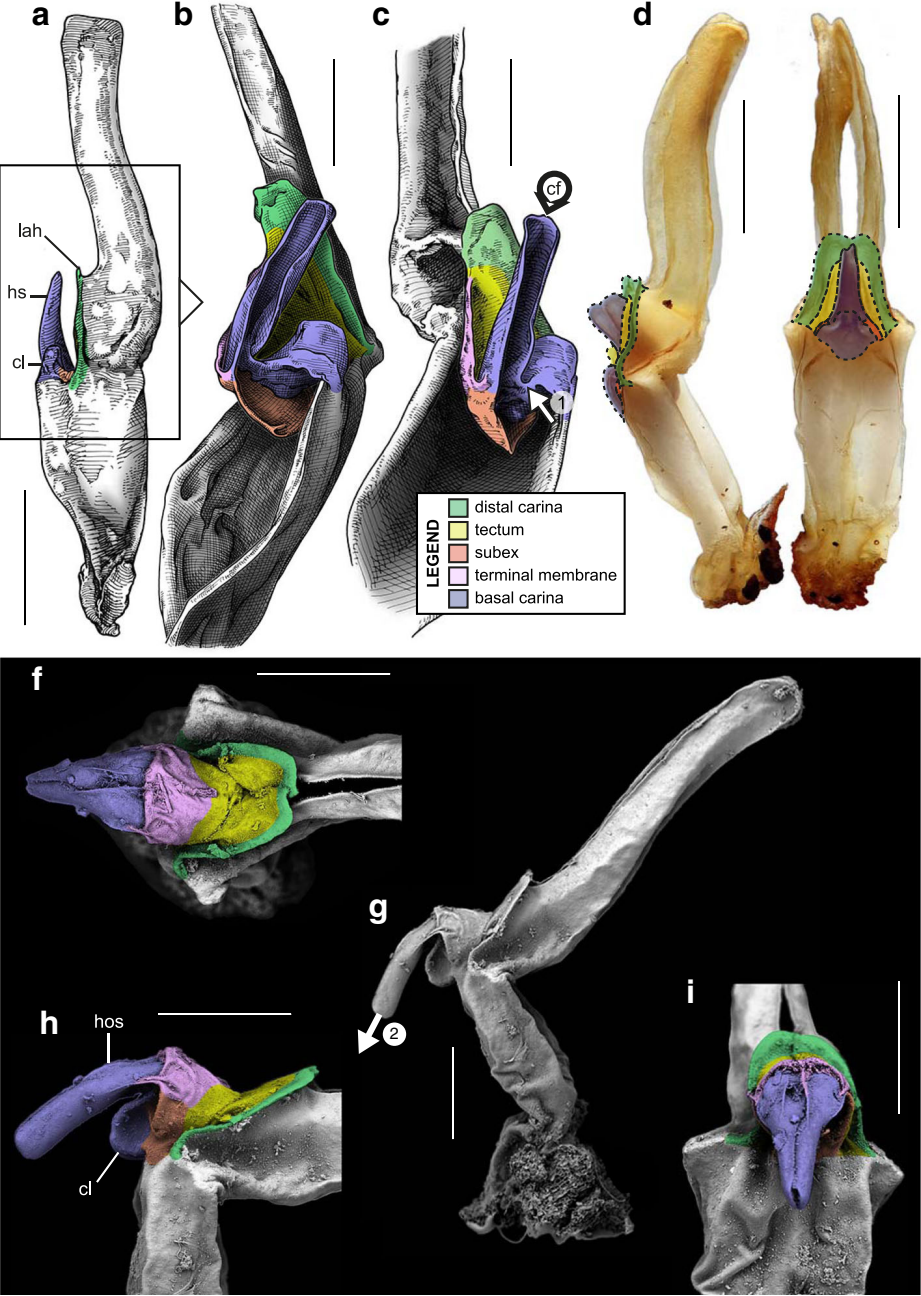
Male reproductive apparatus of *Hormiops davidovi* Fage, 1934 (Vietnam, Conson Island, MHNG). Hemispermatophore (**a** ***–*** **c**). Pre- (**d, e**) and post-insemination (**f–i**) spermatophores. Lateral (**a, d, g, h**), anterior (**b, e, i**), contra-lateral (**c**), and dorsal (**f**) aspects. Arrow 1 shows the invagination of the basal edge of the capsule and arrow 2 indicates the site and direction of semen expulsion. Abbreviations: cf (capsular foramen), cl (clasper), hos (holosolenos), hs (hemisolenos), lah (laminar hook). Scales, 1 mm (**a, d–e**), 0.5 mm (**b–c, f–i**)

**Fig. 22:**
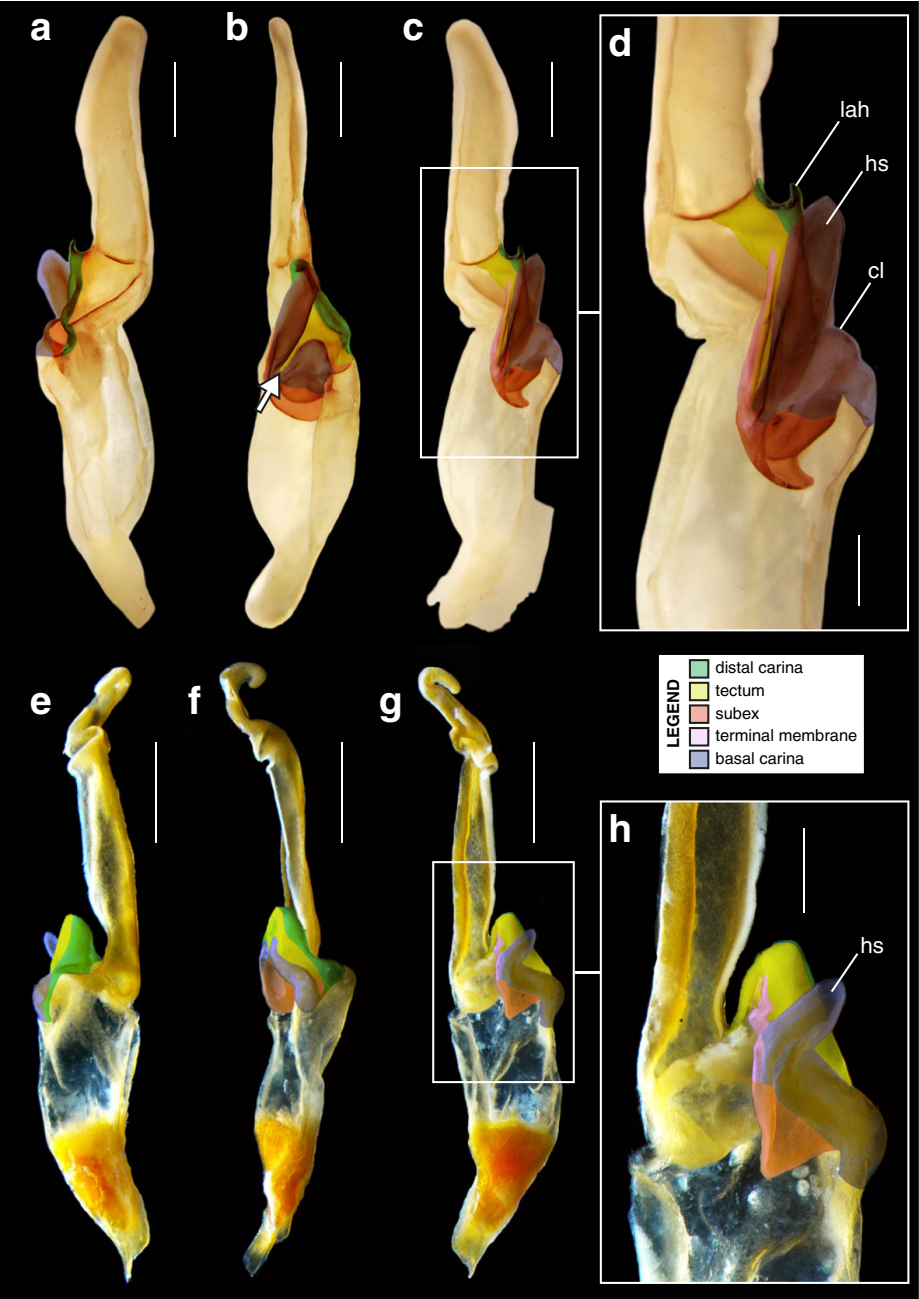
Male reproductive apparatus of Hormuridae Laurie, 1896 (**a**–**b**) and Diplocentridae Karsch, 1880 (**e**–**f**). Hemispermatophore of *Liocheles* cf. *australasiae* (Fabricius, 1775) (Thailand, Trat Province, MHNG) and *Diplocentrus zacatecanus* Hoffmann, 1931 (México, Tepezalá, CNAN-1733). Lateral (**a, e**), anterior (**b, f**), and contra-lateral (**c, d, g, h**) aspects. The white arrow shows the invagination of the basal edge of the capsule. Abbreviations: cl (clasper), hs (hemisolenos), lah (laminar hook). Scales, 1 mm (**a–c, e–f**), 0.5 mm (**d**), 0.25 (**h**)

**Fig. 23:**
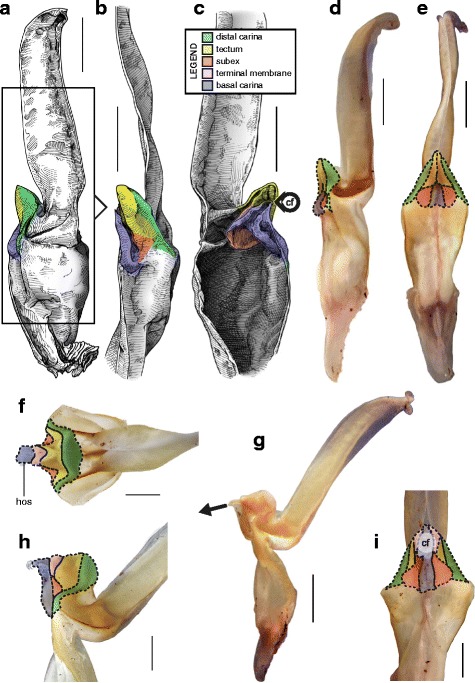
Male reproductive apparatus of Diplocentridae Karsch, 1880. Hemispermatophore of *Nebo yemensis* Francke, 1980 (Yemen, MHNG). Pre- (**d, e**) and post-insemination (**f** ***–*** **i**) spermatophores of *Nebo* cf. *whitei* Vachon, 1980 (Oman, MHNG). Lateral (**a, d, g, h**), anterior (**b, e, i**), contra-lateral (**c**), and dorsal (**f**) aspects. The arrow indicates the site and direction of semen expulsion. Abbreviations: cf (capsular foramen), hos (holosolenos). Scales, 2 mm (**a–e, g**), 1 mm (**f, h–i**)

**Fig. 24:**
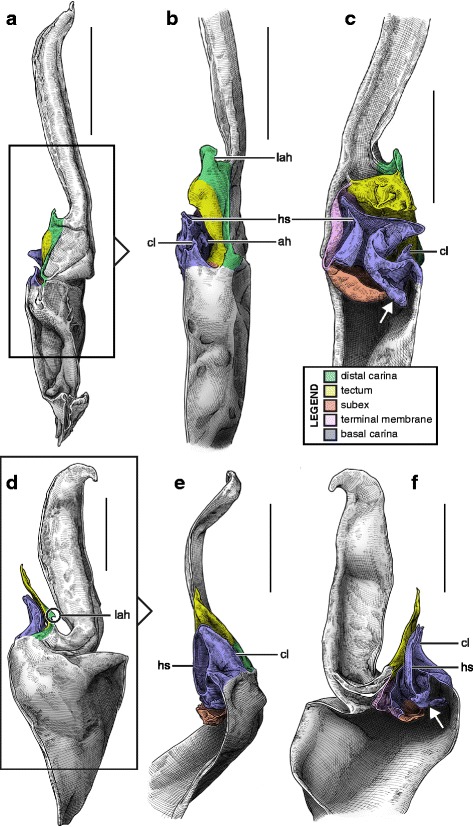
Hemispermatophores of Urodacidae Pocock, 1893. *Urodacus manicatus* (Thorell, 1876) (Australia, Victoria, FKPC) (**a** ***–*** **c**) and *Urodacus hoplurus* Pocock, 1898 (Australia, Western Australia, FKPC) (**d**–**f**). Lateral (**a, d**), anterior (**b, e**) and contra-lateral (**c, f**) aspects. Abbreviations: ah (accessory hook), cl (clasper), hs (hemisolenos), lah (laminar hook). The white arrows show the deep invagination of the basal edge of the capsule. Scales, 3 mm (**a**), 2 mm (**b–f**)

**Fig. 25:**
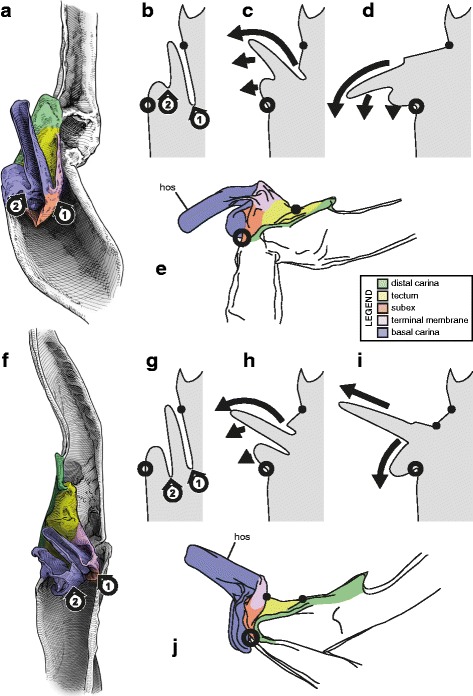
Spermatophores of non-bothriurid Scorpionoidea, *Hormiops davidovi* Fage, 1934 (**a, e**) and *Heterometrus mysorensis* Kovařík, 2004 (**f, j**). Hemispermatophore, contra-lateral aspect (**a, f**). Spermatophore, lateral aspect (**e, j**). Evertion of the sperm duct, detail of the mechanism (**b** *–* **d**, **g** *–* **i**). Abbreviation: hos (holosolenos). The numbered circles with arrow show the invagination of the subex (1) and the fold of the basal carina between clasper and hemi/holosolenos (2). The circles represent the axes of rotation and the dots are fixed points along which membranes are unfolding

**Fig. 26:**
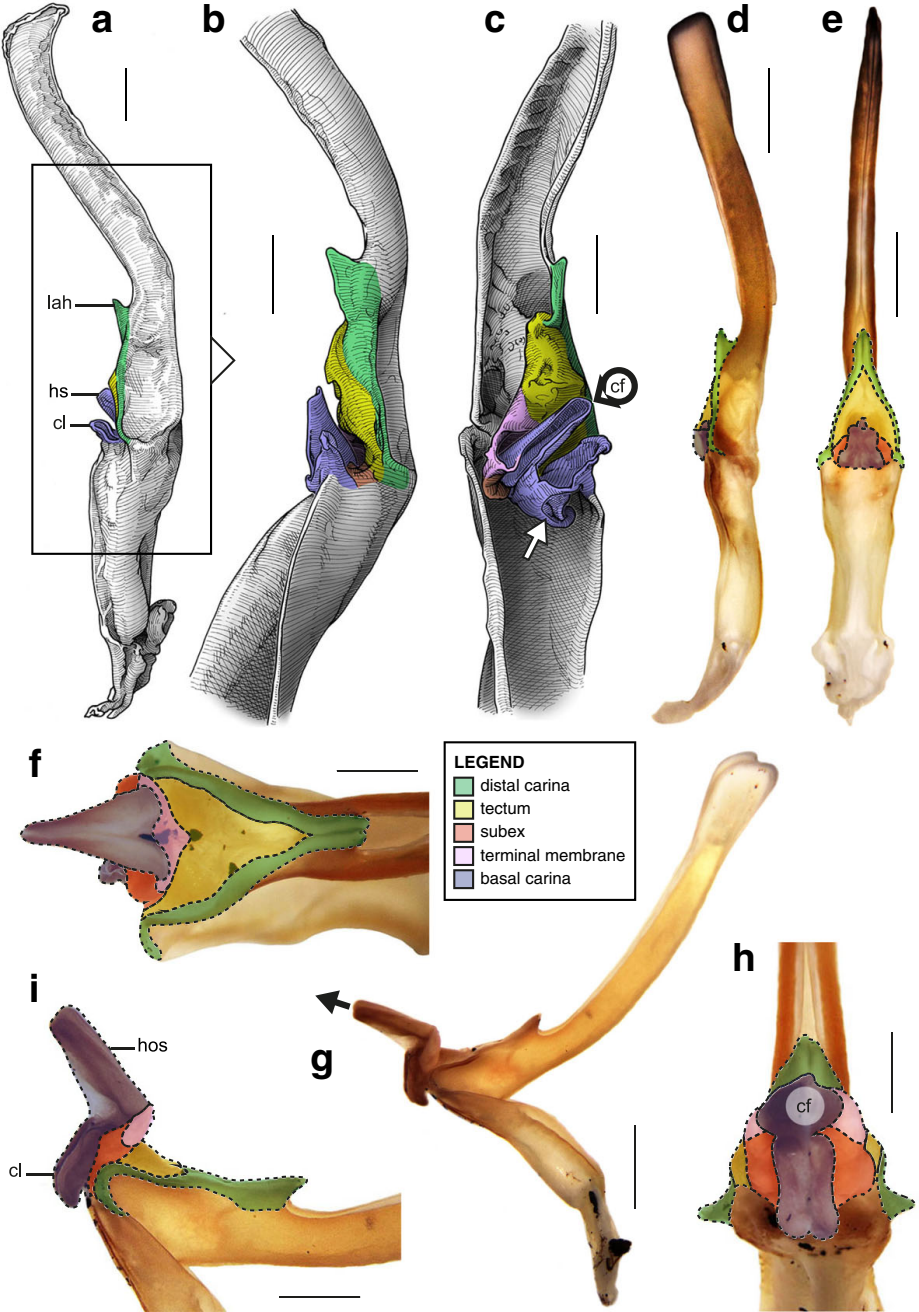
Male reproductive apparatus of Scorpionidae Latreille, 1802. *Heterometrus mysorensis* Kovařík, 2004 (India, MHNG). Hemispermatophore (**a** ***–*** **c**). Pre- (**d, e**) and post-insemination (**f** ***–*** **i**) spermatophores. Lateral (**a, d, g, i**), anterior (**b, e, h**), contra-lateral (**c**), and dorsal (**f**) aspects. The white arrow shows the deep invagination of the basal edge of the capsule. The black arrow indicates the site and direction of semen expulsion. Abbreviations: cf (capsular foramen), cl (clasper), hos (holosolenos), hs (hemisolenos), lah (laminar hook). Scales, 1 mm (**a**–**c**, **f**, **h**–**i**), 2 mm (**d**–**e**, **g**)

**Fig. 27:**
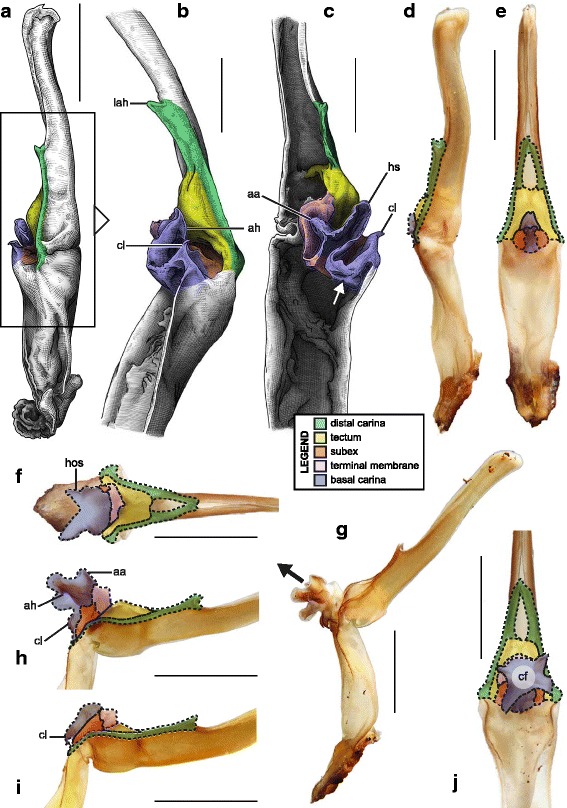
Male reproductive apparatus of the sub-genus *Opisthacanthus* Peters, 1861. *Opisthacanthus* (*Opisthacanthus*) *lecomtei* (Lucas, 1858) (Cameroon, MHNG). Hemispermatophore (**a** ***–*** **c**). Pre- (**d, e**) and post-insemination (**f** ***–*** **j**) spermatophores. Lateral (**a, d, g, h, i**), anterior (**b, e, j**), contra-lateral (**c**), and dorsal (**f**) aspects. The white arrow shows the deep invagination of the basal edge of the capsule. The black arrow indicates the site and direction of semen expulsion. Abbreviations: aa (accessory apophysis), ah (accessory hook), cf (capsular foramen), cl (clasper), hos (holosolenos), hs (hemisolenos), lah (laminar hook). Scales, 2 mm (**a, d–j**), 1 mm (**b, c**)

**Fig. 28:**
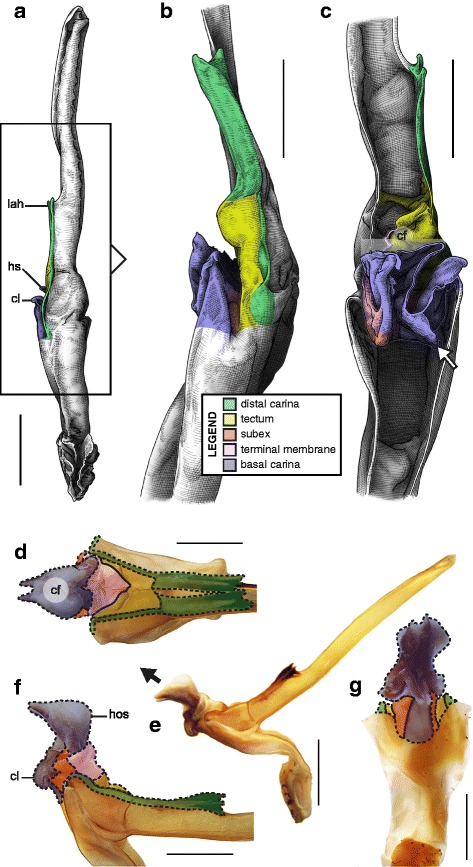
Male reproductive apparatus of *Hadogenes* cf. *paucidens* Pocock, 1896 (Tanzania, MHNG). Hemispermatophore (**a** ***–*** **c**). Post-insemination spermatophore (**d** ***–*** **g**). Lateral (**a, e** ***–*** **f**), anterior (**b, g**), contra-lateral (**c**), and dorsal (**d**) aspects. The white arrow shows the deep invagination of the basal edge of the capsule. The black arrow indicates the site and direction of semen expulsion. Abbreviations: cl (claspers), hos (holosolenos), hs (hemisolenos), lah (laminar hook). Scales, 3 mm (**a**, **e**), 2 mm (**b** *–* **d**, **f** *–* **g**)

**Fig. 29:**
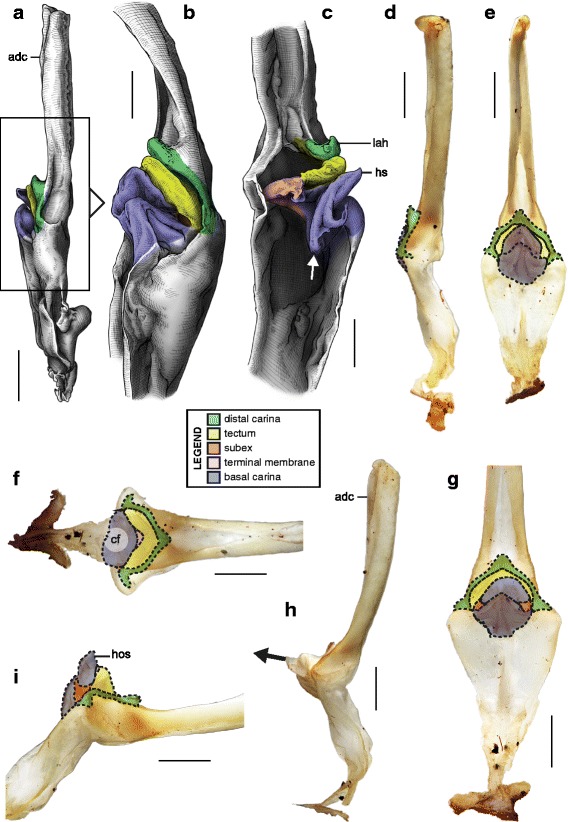
Male reproductive apparatus of *Opisthacanthus* (*Nepabellus*) cf. *validus* Thorell, 1876 (South Africa, Durban, MHNG). Hemispermatophore (**a** ***–*** **c**). Pre- (**d, e**) and post-insemination (**f** ***–*** **g**) spermatophores. Lateral (**a, h, i**), anterior (**b, g**), contra-lateral (**c**), and dorsal (**f**) aspects. The white arrow shows the deep invagination of the basal edge of the capsule. The black arrow indicates the site and direction of semen expulsion. Abbreviations: adc (antero-distal crest), cf (capsular foramen), hos (holosolenos), hs (hemisolenos), lah (laminar hook). Scales, 1 mm (**a, d–i**), 0.5 mm (**b, c**)

**Fig. 30:**
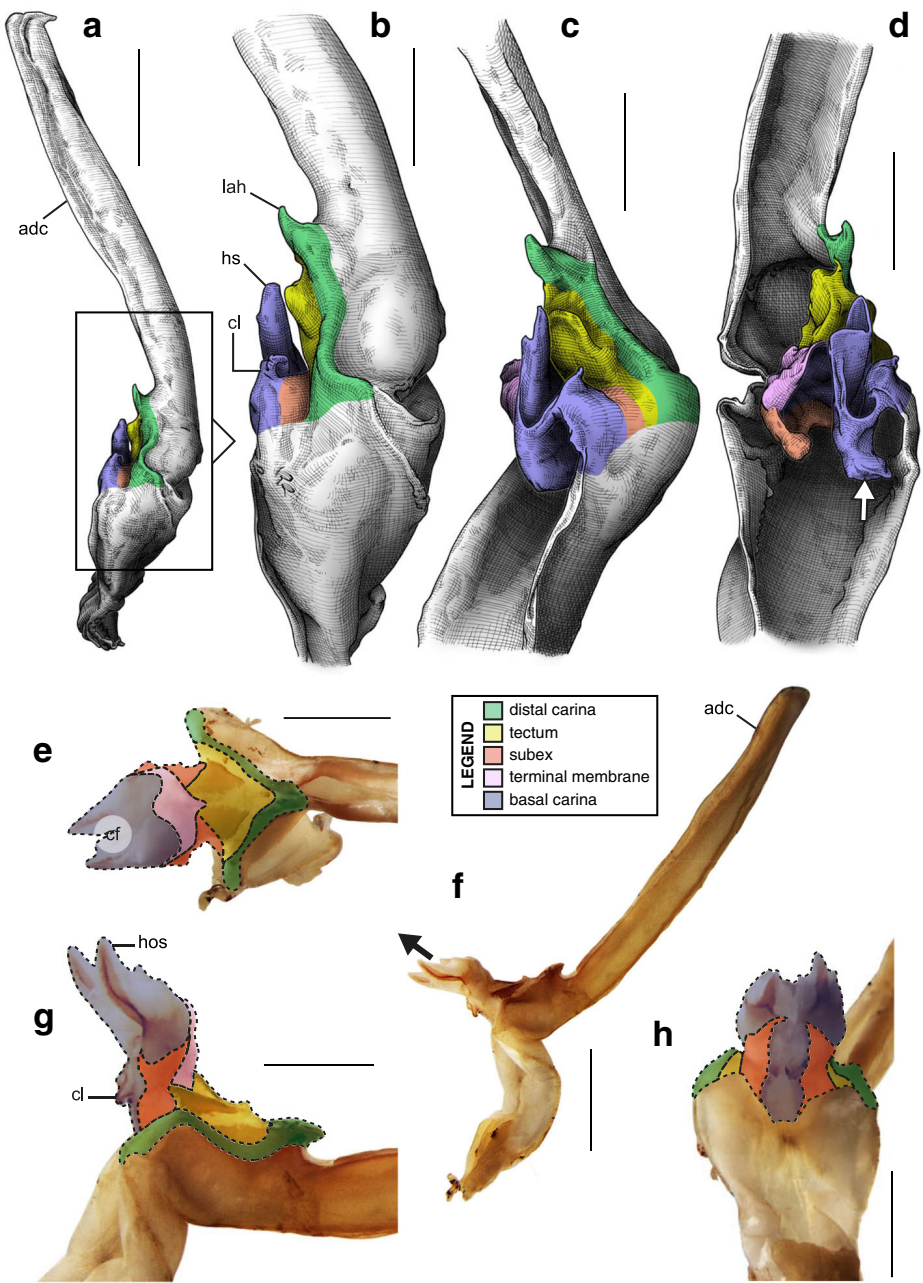
Male reproductive apparatus of *Opisthacanthus* (*Nepabellus*) cf. *asper* (Peters, 1861) (Tanzania, MHNG). Hemispermatophore (**a** ***–*** **d**). Post-insemination spermatophore (**e** ***–*** **h**). Lateral (**a, b, g, f**), anterior (**c**), contra-lateral (**d**), dorsal (**e**), and ventral (**h**) aspects. The white arrow shows the deep invagination of the basal edge of the capsule. The black arrow indicates the site and direction of semen expulsion. Abbreviations: adc (antero-distal crest), cf (capsular foramen), hos (holosolenos), hs (hemisolenos), lah (laminar hook). Scales, 2 mm (**a, f**), 1 mm (**b–e, g–h**)

**Fig. 31:**
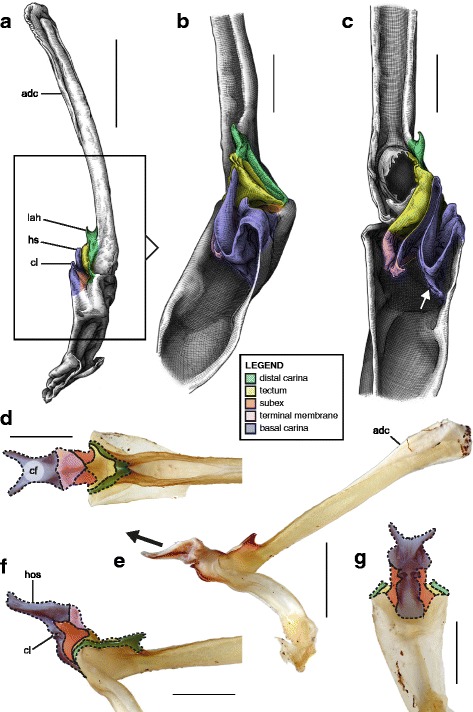
Male reproductive apparatus of *Chiromachus ochropus* (Koch, 1937) (Seychelles, MHNG). Hemispermatophore (**a–c**). Post-insemination spermatophore (**d** ***–*** **g**). Lateral (**a, e, f**), anterior (**b**), contra-lateral (**c**), dorsal (**d**), and ventral (**g**) aspects. The white arrow shows the deep invagination of the basal edge of the capsule. The black arrow indicates the site and direction of semen expulsion. Abbreviations: adc (antero-distal crest), cl (clapsers), hos (holosolenos), hs (hemisolenos), lah (laminar hook). . Scales, 3 mm (**a**), 2 mm (**d–g**), 1 mm (**b, c**)

**Fig. 32:**
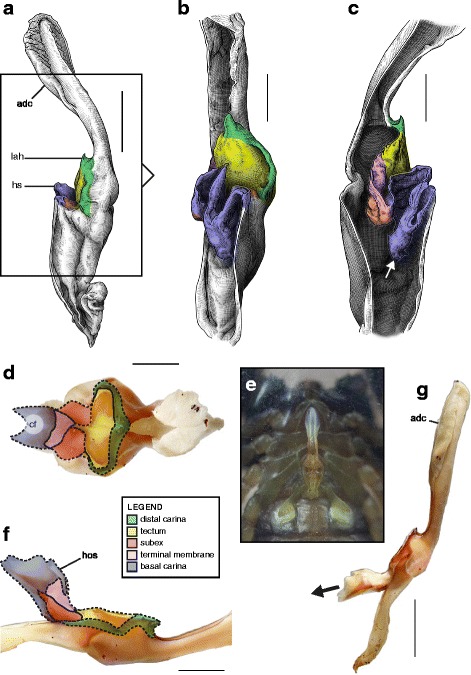
Male reproductive apparatus of the sub-genus *Monodopisthcanthus* Lourenço, 2001. *Opisthacanthus* (*Monodopisthacanthus*) cf. *madagascariensis* Kraepelin, 1894 (Madagascar, Toliara Province, MHNG). Hemispermatophore (**a** ***–*** **c**). Post-insemination spermatophore (**d, f, g**). Lateral (**a, f, g**), anterior (**b**), contra-lateral (**c**), and dorsal (**d**) aspects. Detail of the ventral coxo-sternal region of an inseminated female, showing the spermatophore attached to the coxapophysis II by the lateral crest of the lamina (**e**). The white arrow shows the deep invagination of the basal edge of the capsule. The black arrow indicates the site and direction of semen expulsion. Abbreviations: adc (antero-distal crest), hos (holosolenos), hs (hemisolenos), lah (laminar hook). Scales, 2 mm (**a, g**), 1 mm (**b**–**d, f**)

**Fig. 33:**
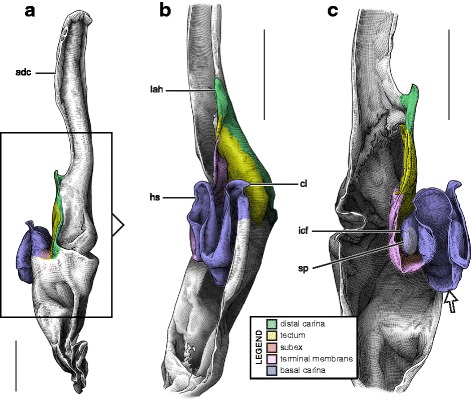
Hemispermatophore of *Iomachus malabarensis* Pocock, 1900 (India, Mangalore, NHML), lateral (**a**), anterior (**b**) and contra-lateral (**c**) aspects. Abbreviations: adc (antero-distal crest), cl (clasper), icf (internal carinal fold), hs (hemisolenos), lah (laminar hook), lah (laminar hook), sp (spicule). The white arrow shows the deep invagination of the basal edge of the capsule. Scales, 1 mm

Hemispermatophores of the families Diplocentridae ([4, 37, 38, 176–180]; Figs. 22e–h, 23a–c), Hemiscorpiidae [41], Heteroscorpionidae (Fig. 20) [35, 36], Hormuridae ([24–28, 49, 94, 181–183]; Figs. 21a–c, 22a–d, 25a–e, 27a–c, 28a–c, 29a–c, 30a–d, 31a–c, 32a–c, 33a–c), Scorpionidae Latreille, 1802 ([3, 5]; Fig. 26a–c) and Urodacidae ([23]; Fig. 24) possess the most complex capsules within the order. In all these taxa the posterior extremity of the basal capsular carina is elongated and invaginated to form a half-duct, i.e. the capsular lamella sensu Monod & Lourenço [41] which is renamed here hemisolenos. In the spermatophore, the hemisolenos of the hemispermatophores are fused together to form a pipe-like structure referred to as the holosolenos (*hos* in Figs. 14e–h, 21f–i, 23f–i, 25, 26f–i, 27f–j, 28d–g, 29f–i, 30e–h, 31d–g, 32d, and f–g). Like the chactoid physema and the vaejovid mating plug, the holosolenos is directly responsible for sperm transfer and insemination: it is an evertible intromittent appendage through which semen is expelled into the female genital tract (*hos* in Fig. 14e–h). Furthermore, in the hormurid *Iomachus politus* Pocock, 1896 and in the sub-genus *Opisthacanthus* Peters, 1861, the holosolenos bears accessory hooks and apophyses (*ah* and *aa* in Fig. 27) that prevent it from slipping out of the female genital tract once inserted. The holosolenos detaches from the spermatophore at the end of a succesfull mating (Fig. 27i) and remains in the female genital tract.

The spermatophore capsule probably also acts as a detachable mating plug in the families Hemiscorpiidae and Urodacidae, as suggested by the morphology of their respective hemispermatophores. In hemiscorpiids, the capsule pattern is very similar to that of *Iomachus politus* and of the subgenus *Opisthacanthus*, with hemisolenos bearing an accessory hook and apophysis [41], but a bifid laminar hook is also present as in *Hadogenes* Kraepelin, 1874 [41]. In urodacid hemispermatophores ([23]; Fig. 24) the propensity of the hemisolenos to detach if the dissection is not performed carefully, and the presence of an accessory hook in some species (*ah* in Fig. 24b) suggest that the holosolenos probably also acts as a mating plug in this family, as already mentioned by Stockwell [4].

In many scorpionoid taxa the anterior extremity of the basal carina is also modified into an apophysis (*cl* in Figs. 20, 21, 22a–d, 24, 26, 27, 28, 30, 31, 33) that can have a hook-like shape (*cl* in Figs. 24, 26, 27, 28, 30, 31, 33). This feature, previously referred to as the distal lobe [41], is considered here as homologous to the bothriurid claspers (see discussion) and is thus called the same.

In addition to the development of accessory processes (clasper and hemisolenos), the overall shape of the basal carina is also modified in non-bothriurid scorpionoids. In all these taxa, except for the Diplocentridae, the basal edge of the capsule is invaginated between clasper and hemisolenos. This fold (= anterior edge of basal lobe sensu Monod [49], arrow 1 in Fig. 4e) can be very shallow as in *Heteroscorpion* Birula, 1903 (white arrow in Fig. 20c), *Hormiops* Fage, 1933 ([94, 182]; white arrow in Fig. 21c, and arrow 2 in Fig. 25a–d), *Hormurus* Thorell, 1876 [183] and *Liocheles* Sundevall, 1833 [181], but in other scorpionoids the invagination is much deeper (white arrow in Figs. 24c, f, 26c, 27c, 28c, 29c, 30d, 31c, 32c, 33c and arrow 2 in 25f–g). The depth of this additional invagination has major repercussions on how the capsule is everted.

If the invagination is absent, as in Diplocentridae (Figs. 22g–h, 23c), or weak as in *Hormiops* (white arrow in Fig. 21c and arrow 2 in Fig. 25a–b), *Hormurus* and *Liocheles* (white arrow in Fig. 22b), the claspers and holosolenos form a solid block, and thus capsule eversion triggers their combined forward rotation (Fig. 25c–d). In post insemination spermatophores (Figs. 21f–i, 23f–i) the holosolenos is thus reoriented anteriorly and sits approximately parallel to the stalk. Given the shallowness of their basal capsular invagination (white arrow in Fig. 20c), *Heteroscorpion* spermatophores presumably follow the same unfolding process.

The deeper carinal invagination observed in other scorpionoid taxa (white arrow in Figs. 24c, f, 26c, 27c, 28c, 29c, 30d, 31c, 32c, 33c) translates into a more balanced architecture with deep folds on both sides of the holosolenos (Fig. 25g). In simple terms, the structure is comparable to a double origami sink fold. The carinal invagination creates a weak spot on the basal carina between clasper and hemisolenos. This point of least resistance acts as an additional hinge when the sperm duct membrane is unfolded during capsular eversion. As a result, the holosolenos does not rotate together with the claspers but is pulled out with only a slight rotation (Fig. 25h–i). The additional fold also translates into an increase of the outward extension of the sperm duct. Post-insemination spermatophores then show a holosolenos sitting more perpendicularly relative to the axis of the stalk (*hos* in Figs. 26f–h, 27f–j, 28d–g, 29f–i, 30e–h, 31d–g, 32d, f–g), and a sperm duct that is more elongated. This last point is especially obvious in *Chiromachus ochropus* (Koch, 1837) (Fig. 31d–g), *Opisthacanthus* (*Nepabellus*) cf. *asper* (Peters, 1861) (Fig. 30e–h) and *Monodopisthacanthus* spp. (Fig. 32d–g).

In *Chiromachus ochropus*, *Monodopisthacanthus* and *Nepabellus* the spermatophore remains attached to the female genital tract after insemination, sometimes for several hours. The distal lamina in all these taxa bears a pronounced antero-distal crest (‘lateral crest’ sensu Lamoral [3]) (*adc* in Figs. 29, 30, 31, 32). This crest appears to be adhesive and will stick to the female coxapophyses II (Fig. 32e), holding the spermatophore in place and the sperm duct inside the female genital tract, probably to ensure that insemination is carried out properly and that the female does not reject semen. Although the courtship of *Palaeocheloctonus* Lourenço, 1996 and Indian *Iomachus* Pocock, 1893 was not observed, a similar antero-distal crest is present on hemispermatophores of these taxa (*adc* in Fig. 33a) and, given their close relationship with *C. ochropus* and *Monodopisthacanthus* [49, 50], probably fulfils the same function. In *Opisthacanthus* (*Nepabellus*) *validus* Thorell, 1876 the male detaches the spermatophore from the female with its anterior legs after insemination (Monod, four unpublished observations).

Hemispermatophores of Indian *Iomachus* possess a capsule more complex than in other scorpionoids (Fig. 33b–c). In addition to the invagination between clasper and hemisolenos, the anterior part of the carina that bears the clasper also extends inward around the hemisolenos, thus creating an additional inner fold between the hemisolenos and the subex (*icf* in Fig. 33c). Although spermatophores remain unknown for these Indian taxa, the inner carinal fold (*icf*) is probably also unfurled during capsular eversion, as indicated by the presence of small spinules (*sp* in Fig. 33c) on its inner wall. Like other features present on the sperm duct, these spinules probably help to create a seal between the spermatophore and the female genital tract, and thus must be in contact with the walls of the latter during insemination. Because they are situated on the side of the fold concomitant with the hemisolenos, the usual unfolding observed in scorpionoids is not sufficient to expose the spicules and an unfolding of the inner carinal fold is thus necessary.

It is also important to point out that the *hoplurus*-group of the genus *Urodacus* [23] possesses hemispermaphores with a strongly modified morphology (Fig. 24d–f) in comparison to the rest of the non-bothriurid scorpionoids. In hemispermatophores of this species group the tectum is flattened against the distal carina and forms a very slender process. The hemisolenos is compressed in a similar fashion, and the claspers are much bigger than in other genera, sometimes having serrated edges. Despite these pronounced differences in the proportions of the capsular elements, the bauplan is the same.

### Ancestral state reconstructions of the Bauplans

**Fig. 34:**
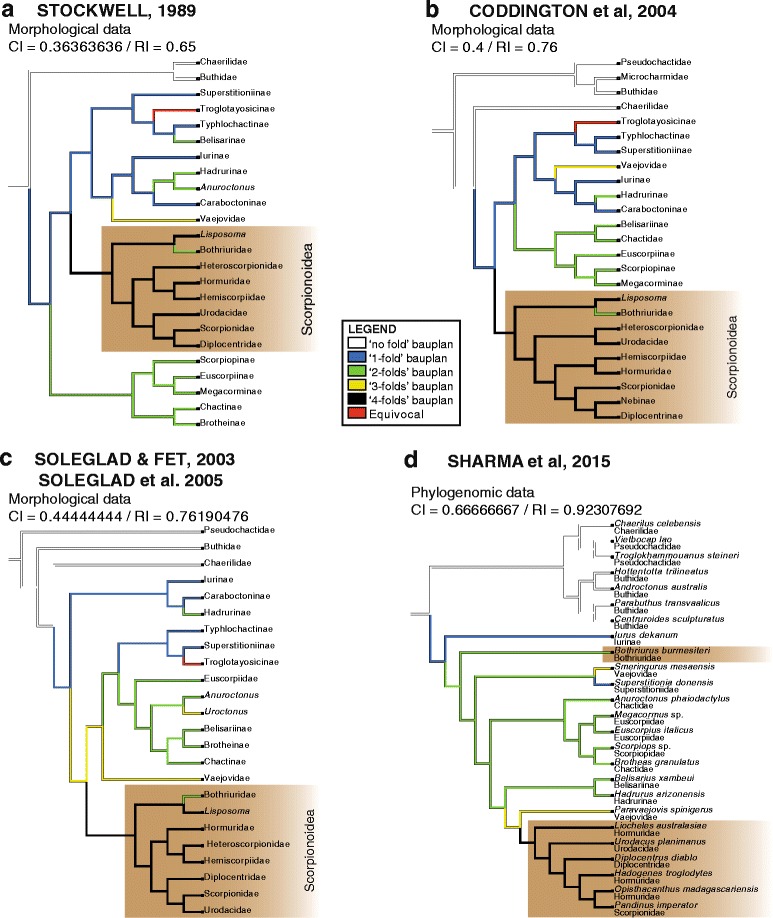
Ancestral state reconstruction of the capsular design (bauplans) on phylogenies of the order Scorpiones by Stockwell [4] (**a**), Coddington et al. [104] (**b**), Soleglad & Fet [46]/Soleglad et al. [105] (**c**), and Sharma et al. [106] (**d**). Taxa, traditionally recognized as scorpionoids, are indicated in brown. Consistency and retention index values are indicated for each cladogram

Four phylogenies of the order Scorpiones are currently available. Three are based on morphological data [4, 46, 104, 105] and one on phylogenomic data [106]. In order to assess the evolution sequence of the male reproductive apparatus, character optimization of the bauplans was conducted on these four cladograms. In all of these hypotheses, the ‘four-folds’ bauplan is evolved only once; the ‘no-fold’ bauplan is recovered as plesiomorphic for the order Scorpiones, and the ‘one-fold’ bauplan as plesiomorphic for the parvorder Iurida Solegad & Fet, 2003 (Fig. 34).

In phylogenies by Stockwell [4] (Fig. 34a) and Coddington et al. [104] (Fig. 34b), the ‘one-fold’ and ‘three-folds’ bauplans each evolve only once, respectively from a ‘no-fold’ and ‘one-fold’ ancestor. On the other hand, the ‘two-folds’ bauplan is evolved four times in Stockwell’s [4] (three times from a ‘one-fold’ ancestor and one reversal from a ‘four-folds’ ancestor) and three times in Coddington et al.’s [104] (twice from a ‘one–fold’ ancestor and one reversal from a ‘four-folds’ ancestor). Because of the lower number of independent emergences, the bauplan obtains better consistency and retention indexes on Coddington et al.’s cladogram [104] (CI = 0.4 / RI = 0.76) than on Stockwell’s [4] (CI = 0.36363636 / RI = 0.65).

In the phylogeny by Soleglad & Fet [46]/Soleglad et al. [105] (Fig. 34c), the ‘two-folds’ bauplan is evolved three times (once from the ‘one-fold’ ancestor, one reversal from a ‘three-folds’ ancestor and one reversal from a ‘four-folds’ ancestor), while the ‘three-folds’ bauplan is evolved twice (once from a ‘one-fold’ ancestor and one reversal from a ‘two-folds’ ancestor). Contrary to Stockwell’s [4] and Coddington et al.’s [104], the ‘four-folds’ bauplan evolves here from a ‘three-folds’ ancestor. The consistency and retention indexes of the bauplan on Soleglad & Fet [46]/Soleglad et al.’s phylogeny [105] are similar to those on Coddington et al.’s [104].

In the cladogram by Sharma et al. [106] (Fig. 34d), the evolution from the ‘no-fold’ to the ‘four-folds’ bauplan is incremental, each pattern evolving from the precedent in a linear fashion. The numbers of parallel evolution and reversal events is lower than in the preceding trees: the ‘three-folds’ bauplan is evolved twice independently from ‘two-folds’ ancestors, and there is one reversal from ‘two-folds’ to ‘one-fold’ pattern. The consistency and retention indexes of the bauplan (CI = 0.66666667 / RI = 0.92307692) are thus higher than in preceding trees.

### Ancestral state reconstructions of the invagination of the basal edge of the capsule

**Fig. 35:**
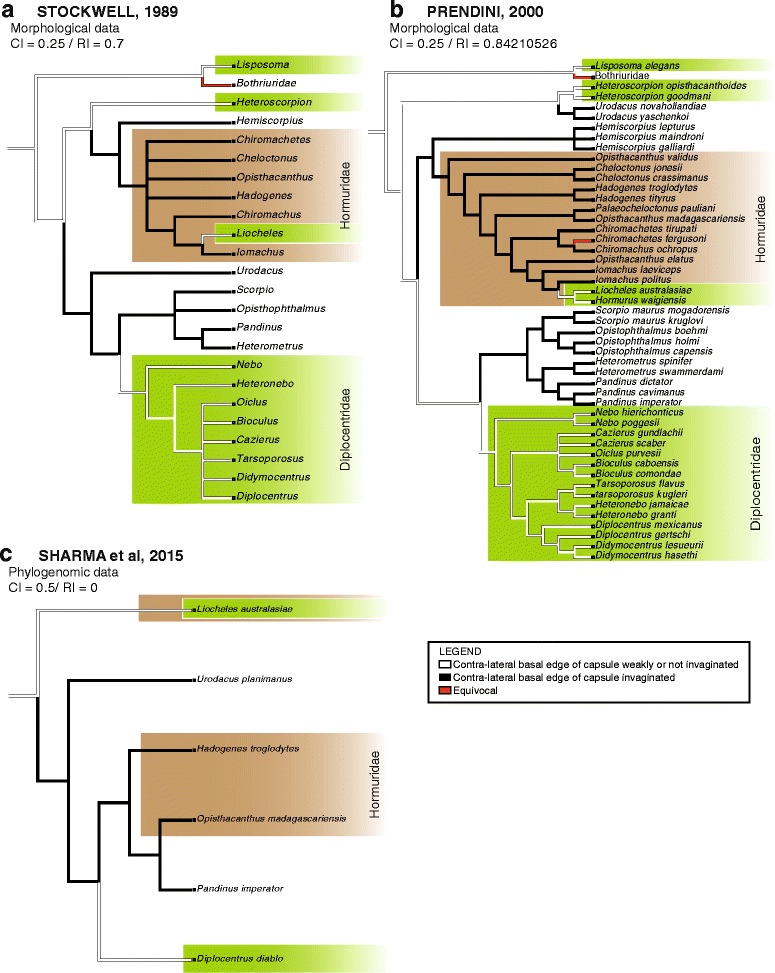
Ancestral state reconstruction of the absence/presence of a capsular basal invagination on phylogenies by Stockwell [4] (**a**), Prendini [45] (**b**), and Sharma et al. [106] (**c**). Taxa, traditionally recognized as hormurids, are indicated in brown, and taxa possessing a weak or absent invagination of the basal capsular edge in green. Consistency and retention index values are indicated for each cladogram

In the three phylogenies (Fig. 35), a weak or absent invagination of the basal capsular edge is retrieved as plesiomorphic.

In Stockwell [4] (Fig. 35a), the deep invagination evolves twice with two subsequent reversals to the weak/absent state (in *Liocheles* and Diplocentridae). In Prendini [45], (Fig. 35b), this derived pattern is evolved three times with only one reversal (*Hormurus*/*Liocheles*). The consistency index is the same for both phylogenies (CI = 0.25), but the retention index is slightly better in Prendini [45], (RI = 0.84210526) than in Stockwell [4] (RI = 0.7).

In the phylogeny by Sharma et al. [106] (Fig. 35c), the deep invagination evolves only once with a subsequent reversal to the plesiomorphic state in Diplocentridae.

## Discussion

### Reliability of data gathered from published illustrations

A large part of the present dataset is based on the interpretation of illustrations from published taxonomic descriptions and one can question the reliability of these data, especially given the difficulty of accurately depicting the tridimentional shape of the capsule region in photographs or drawings.

The strong conservatism of in-toto shapes generally observed within taxonomic groups enables easy classification of hemispermatophores according to their gross morphology. There is no reason to believe that hemispermatophores from closely related taxa with a similar overall shape have different capsules. Therefore, based on comparison with known general morphologies, published illustrations of hemispermatophores generally allow accurate determination of the bauplan even if the capsule is not properly depicted.

For only a few taxa, i.e. the genus *Lisposoma*, the families Pseudochactidae and Superstitioniidae, were the illustrations insufficient to assign a bauplan with certainty. These few ambiguous cases, however, do not weaken the overall results and conceptual framework of the present study; the five structural patterns proposed here were unambiguously identified in a sufficiently large range of taxa to confirm their validity. It nevertheless remains possible that the few hemispermatophores mentioned above represent further bauplans to be added to the ones characterised here.

### Intromittent structures used for insemination

The comparative study of pre- and post- insemination spermatophores allowed accurate determination of the structure performing the insemination per se for each taxon studied. It appears that distantly related taxonomic groups use different intromittent features of the capsule to guide the semen into the female gonopore. Based on their position on the capsule, it was then possible to reassess homology between these different traits.

Across the order, insemination is carried out by three different intromittent structures on the spermatophore: the chactoid physema, the vaejovid mating plug and the non-bothriurid scorpionoid holosolenos. Only the non-bothriurid scorpionoid holosolenos and the vaejovid mating plug are here considered to be homologous because they are sclerotized parts of the basal capsular carina. Moreover, the fact that the holosolenos acts as a detachable mating plug in several non-bothriurid scorpionoid taxa (*I. politus*, the sub-genus *Opisthacanthus* Peters, 1861 and probably also in the genera *Hemiscorpius* and *Urodacus*), gives more support to this hypothesis. On the other hand, the physema of chactoids is neither considered homologous to the holosolenos and nor to the mating plug, because it is not derived from the basal carina but is formed by an enlargement of the terminal membrane of the sperm duct.

### Claspers and holosolenos

A conspicuous eversible sperm duct that protrudes from the capsule and guides semen into the female genital tract is traditionally considered synapomorphic for the spermatophores of Scorpionoidea [4, 45]. However, analysis of the insemination mechanism suggests that the protruding capsular feature observed in Bothriuridae is not the same structure as in the other taxa of the superfamily.

Bothriurid claspers were previously considered to be homologous to the holosolenos of non-bothriurid Scorpionoidea [4]. This, however, is here refuted by the comparison of used spermatophores and the insemination processes in the two groups. Claspers and holosolenos appear to have different functions. The holosolenos forms a channel for semen expulsion in scorpionoids (*hos* in Fig. 14e–h), whereas the paired bothriudid claspers do not contribute directly to sperm transfer but rather act as oversized hooks that help widen the female genital atrium and provide secure anchoring for the spermatophore, thus preventing sperm backflow ([55, 85–87]; *cl* in Fig. 14c–d). In bothriurids insemination is carried out by the physema, as is the case in Chactoidea ([55, 85–87]; *ph* in Fig. 14a–b).

Jacob et al. [90] had previously suggested that the bothriurid claspers are homologous to the crown-like structures observed in some chactoid spermatophores, because they are in a similar position beneath the capsular foramen and have a similar function, i.e. widening of the female genital atrium and helping to anchor the spermatophore. The presence of membranous crests extending basally from the crown-like structures in chactoids (*bcr* in Figs 11d–e, 12e–f) or from the claspers in bothriurid (*bcr* in Figs. 15c and h, 16b–c) tends to corroborate this interpretation. In non-bothriurid scorpionoids a pair of capsular apophyses (= distal lobe sensu Monod & Lourenço [41], see next paragraph) assumes the same position (*cl* in Fig. 14e–h) and presumably also the same function as the bothriurid claspers during insemination (*cl* in Fig. 14c–d). For this reason these scorpionoid structures are deemed homologous to the claspers, and are also referred to as claspers in the present contribution.

### Capsular and laminar hooks

Many hemispermatophores possess strong hooks on either or both of the capsular carinae and/or on the basal half of the stalk. These prongs have many different shapes and proportions across the order, but they can easily be divided into two different groups depending on their position on the capsule, i.e. the basal hook (located below the capsular foramen) and the laminar hook (located above the capsular foramen), which are here considered as non-homologous.

The basal hook observed in buthids and iurids is located on the capsular basal carina (*bh* in Figs. 6, 7) and might be homologous to the bothriurid/scorpionid claspers. The laminar hook, on the other hand, is positioned on the distal carina, either laterally (*lh* in Figs 7, 8a–d, 10, 11, 12, 13, 17, 18, 19) or on the anterior edge (*lah* in Figs. 20, 21, 22a–d, 24, 25, 26, 27, 28, 29, 30, 31, 32, 33). The shape, size and number of laminar hooks are extremely variable among different taxonomic groups. The diversity of capsular hooks is arguably a consequence of the morphological variability of the female genital operculum that covers the gonopore.

The operculum of females is composed of two sclerites that can be completely independent of each other, or partly or completely fused to each other depending on the taxon [3, 4, 34, 184]. Basal hooks and lateral laminar hooks are known from taxa with a bipartite genital operculum, whereas anterior laminar hooks are only observed in taxa with operculum halves which are partly or completely fused. The structural difference of the female genital operculum exerts very different mechanical constraints on the spermatophores of the respective groups, i.e. the pivotal axis of a single sclerite is very different from that of a bipartite operculum, hence the two types of capsular hooks.

Although not homologous, basal and laminar hooks have the same function, i.e. to pry open the female genital operculum and atrium [29, 55, 63, 90]. Alexander [64] suggested that bending of the buthid spermatophore is triggered by the hooking of the basal prongs to the inner edges of the female pectines. However, the size of the spermatophore and the position of the basal hooks contradict this hypothesis; if the prongs were hooked to the pectines, the capsular foramen of the spermatophore would be situated between the pectines, far away from the female gonopore, just above the basal plate, where semen would be expelled, failing to inseminate the female. The basal hooks of buthids are more likely to be jammed against the bipartite operculum, ensuring that the spermatophore foramen is in contact with the female gonopore.

### “Safe sperm transfer”, “revealing obstacles”, “female choice by mechanical fit” and the coevolution of female genitalia and spermatophore

The respective importance of natural and sexual selection in the evolution and diversification of genitalia are still debated in most animals [185]. It is usually accepted that the morphological evolution of scorpion copulatory male structures is the result of complex synergetic interactions between a variety of selective pressures [54]. Among those hypotheses, the “safe sperm transfer” [54, 90, 186, 187] is particularly important to understand the evolution of the capsular general architecture. This model suggests that natural selection guides spermatophore evolution, favouring morphologies that would provide a tighter copulatory lock with the female genitalia in order to improve sperm transfer. Data collected during this study suggest that evolution of the spermatophore capsule tends towards structures that arguably provide a better anchor to the female gonopore in agreement with this hypothesis. However, it seems that sexual selection was also of paramount importance in the emergence of more efficient capsules. The female may actually exert passive selection by her copulatory behaviour and genital morphology, thus channeling the evolution of the male copulatory structures.

While the hemispermatophore capsule of more basal taxa such as chaerilids and iurids [46, 104, 106] forms a simple non-intromittent opening (Figs 5 and 6), successive foldings and invaginations of the sperm duct membrane has led to the establishment of increasingly long, eversible intromittent structures in the more derived groups such as scorpionoids [46, 104, 106] (Figs. 20, 21, 22, 23, 24, 25, 26, 27, 28, 29, 30, 31, 32, 33). A similar evolutionary sequence from a non-intromittent to an increasingly complex intromittent reproductive apparatus is observed in opilionids, where primitive groups employ non-intromittent spermatophores whereas more derived groups possess intromittent penises [188].

Natural and sexual selection are both regarded here as major driving forces in the evolution of scorpion hemispermatophores. The “safe sperm transfer” hypothesis [90, 186, 187] accounts in part for the gradual complexification of the sperm duct folds; natural selection might be expected to drive the evolutionary tendency toward longer eversible ducts likely to improve sperm transfer success by minimizing loss. However, sexual selection in the form of “revealing obstacles” [189] and “female choice by mechanical fit” [190] probably also played a prominent role in the evolution of the morphological diversity of the capsule.

“Revealing obstacles” are female adaptations, usually behavioural, that increase “resistance” toward mating males but are independent of male phenotypes. They constrain all males to perform a difficult task in order to be able to mate successfully regardless of female preferences, because the female behaviour is always the same towards all males. The fitness of each male is thus indirectly assessed by overcoming the female resistance and demonstrating its physical and behavioural qualities, or the lack thereof.

In order to mate successfully, the male scorpion, after having deposited the spermatophore on the substrate, needs to pull the female over it to trigger its flexion and thus the sperm transfer. The female does not usually move over the spermatophore on her own accord, even when receptive to the male, but rather sits still and lets herself be guided by the male. In the laboratory several mating attempts failed because of the small size of the males, which were unable to coerce their larger mate over the spermatophore (Monod & Cauwet, unpublished data). Moreover, females are also known to sometimes disrupt courtship by moving away from the axis of the deposited spermatophore either voluntarily or not [87]. Therefore selective pressures are likely to favour spermatophores with features that enable a steady fit onto the operculum and that prevent the female from interrupting insemination [54, 55].

In derived spermatophores eversion of the capsule precedes the expulsion of the spermatozoa [85], suggesting that interactions between spermatophore and female genitalia are primordial to ensure successful insemination. The morphological evolution of the male copulatory structures is probably tightly correlated to that of the female. In the basal taxa successful insemination only seems to be enabled by the various capsular hooks, whereas eversible sperm ducts, which became longer over evolutionary time, appear to be necessary in more derived taxa. It is postulated here that more complex spermatophore structures emerged at least in part in response to evolution of the female genital morphology. Eberhard [190] proposed that females could influence the evolution of male reproductive structures by passive mechanical discrimination. According to this model, the scorpion females may passively favour those males with spermatophores which provide the strongest copulatory grip to their genitalia and are less likely to be dislodged during copulation.

A tight mechanical connection between spermatophore and female genitalia is necessary to ensure sperm transfer and the maintenance of such a copulatory fit requires coevolution of the two structures [191]. The constant adaptation of the spermatophore capsular design to the morphological changes of the female genitalia (for instance the transition from a bipartite towards a fused operculum) is thus needed to maintain a functional interface between the two structures. Evolution acts here to preserve rather than improve safe sperm transfer. Each bauplan is arguably only suited to a certain type of female genitalia, like a lock-key system. The more derived spermatophore architectures, with long eversible holosolenos, are thus not necessarily adapted to every female genitalic pattern. This translates into the persistence of intermediate ‘suboptimal’ forms of capsules over time, as is the case in the evolution of other complex structures [192].

The most elongated ducts, i. e. the holosolenos, are only known from taxa where female opercula are partly or completely fused (non-bothriurid Scorpionoidea), whereas spermatophores with simpler or shorter ducts are prevalent in taxa where females possess a bipartite operculum. Furthermore, in taxa where the operculum is divided but joined by a membrane, as in the Chactinae, Bothriuridae, Vaejovidae, Scorpiopidae, *Uroctonus* and *Uroctonites*, the intromittent part of the spermatophore appears to be longer than in taxa with completely disjoint opercular sclerites. This suggests that interactions between the groove formed by a divided operculum and the capsular hooks in the basal groups are sufficient to prevent the spermatophore from moving sideways during insemination, whereas a mono-sclerite operculum does not permit an efficient lateral blockage of the structure, hence the need for the development of additional features (holosolenos, mating plugs and adhesive laminar crests) to secure the anchoring of the spermatophore to the female gonopore.

### Functional constraints and phylogenetic value

Traits directly involved in biological mechanisms are less likely to evolve quickly and randomly because functional constraints limit their morphological variability [57–60]. When a structure is integrated into a biological mechanism, its shape cannot be quickly and radically modified without causing severe functional disruption. Therefore the significant morphological variability of spermatophores is usually limited to proportions and shapes, whereas each bauplan described in the present contribution is conserved across a wide range of taxa. This suggests that hemispermatophores, and probably also other functionally constrained structures, can be more informative at higher taxonomic levels than external not functionally constrained morphology.

On the other hand, functionally constrained characters are also expected to show higher level of homoplasy than characters with an infinite morphological space [193–195]. However, as evolutionary distance increases, convergent or parallel evolution of similar complex structures and reversals towards lost structures tends to become highly unlikely. As a result, homoplasy between very distantly related taxa should in theory remain rare [196]. Ancestral states reconstructions of the bauplan presented here (Fig. 34) seem to confirm these postulates. Despite several occurences of convergence, parallelism, and reversal in each of the phylogenies, there is never a reversal towards the ‘no-fold’ bauplan. This suggests that initiation of the sperm duct folding prevents reversal towards the most plesiomorphic state. Moreover, the more complex pattern, the ‘four-folds’ bauplan, is evolved only once in all phylogenetic reconstructions, confirming the improbability of parallel or convergent evolution of complex features. Therefore, morphological dissimilarities of spermatophore bauplans within the major taxonomic groups are not expected or should at most remain rare.

However, two cases of spermatophore morphological incongruence within higher-level clades raise questions: (1) Except for the genus *Lisposoma*, the Bothriuridae, traditionally regarded as a basal scorpionoid family (Fig. 34a–c), possess spermatophores with the ‘two-folds’ bauplans rather than the ‘four-folds’ bauplan observed in the rest of the superfamily; (2) The clade (*Hormiops* Fage, 1933 (*Hormurus* Thorell, 1876 *+ Liocheles* Sundevall, 1833)) (HHL clade), usually retrieved as derived in Hormuridae (Fig. 35a–b), possess hemispermatophores and spermatophores more akin to those of Diplocentridae and Heteroscorpionidae than to those of other hormurids. Both these cases are discussed below in more detail.

Interestingly, the phylogenetic position of Bothriuridae and of the HHL clade inferred in a recent analysis based on transcriptomic data [106] (Figs. 34d, 35c), is more congruent with the grouping of taxa based on the morphological similarity of their hemispermatophores than with earlier phylogenetic reconstructions based on morphology [4, 45, 46, 104] (Fig. 34a–c and 35a–b). Although the cladogram from Sharma et al. [106] should be taken with some skepticism given the very limited number of taxa included in the study and the propensity for severe systematic bias in phylogenomic studies [197–199], it nonetheless emphasizes potential flaws in earliers morphological phylogenies.

### Hemispermatophore morphological incongruence in Bothriuridae

In all morphological phylogenies, Bothriuridae are retrieved as basal Scorpionoidea (Fig. 34a–c) [4, 45, 46, 104], whereas they are placed very basally in Iurida outside of Scorpionoidea in the phylogeny presented by Sharma et al. [106] (Fig. 34d). On the other hand, the capsule morphology of most of bothriurid genera shows a ‘two-folds’ pattern like that in the Chactoidea, whereas spermatophores of the remaining scorpionoids show the ‘four-folds’ bauplan. Among the bothriurids, only the African *Lisposoma* can be considered as a true scorpionoid if hemispermatophore morphology alone is considered.

This genus, considered as the most basal bothriurid, possesses hemispermatophores with a morphological pattern different from that of other bothriurids and more similar to that non-bothriurid scorpionoids. Moreover, whereas the female genital sclerites are always disjunct in bothriurids, they are fused in *Lisposoma* [3, 4, 45, 152, 153], a character only observed in non-bothriurid scorpionoids. The morphology of the female genital operculum therefore indicates that the insemination mechanism is different to that of bothriuridae and is probably more akin to that of the other scorpionoids (see paragraph above for details). Among scorpionoids, diplocentrid hemispermatophores with relatively simple hemisolenos (Fig. 22e–h, 23) appear to be morphologically closest to that of *Lisposoma*.

Three hypotheses can be considered here: (1) *Lisposoma* is not a bothriurid: it remains in Scorpionoidea while Bothriuridae are placed more basally in Iurida; (2) *Lisposoma* is a bothriurid and Bothriuridae are scorpionoids: *Lisposoma* hemispermatophores would then represent the plesiomorphic state for the family with subsequent reversal towards the chactoid morphology in the other more derived genera; (3) *Lisposoma* is a bothriurid but Bothriuridae are not scorpionoids, placing more basally in Iurida as in Sharma et al. [106] (Fig. 34d). When considering hemispermatophore capsular pattern, the first hypothesis appears to be the most parsimonious with no convergent or reversal event involved. The second hypothesis imply a reversal from the ‘four-folds’ towards the ‘two-folds’ bauplan, while an additional independent evolution of the ‘four-folds’ bauplan from a ‘two-folds’ ancestor is needed in the third. Unfortunately, *Lisposoma* has not been included in any molecular phylogeny. Thus the position of this basal bothriurid genus needs first to be empirically tested in a molecular phylogenetic framework before unambiguous conclusions can be drawn regarding the phylogenetic position of the Bothriuridae.

### Hemispermatophore morphological incongruence in Hormuridae

The HHL clade is placed as derived in Hormuridae according to several morphological phylogenies [4, 45, 46, 49, 50]. However, the hemispermatophore capsular pattern suggests that the genera *Hormiops*, *Hormurus* and *Liocheles* are actually not hormurids. The folding of the sperm duct in the HHL clade (Figs 21, 22a–d, 25a–e) is very similar to that of diplocentrids (Figs 22e–g, 23) and heteroscorpionids (Fig. 20), more so than to hemispermatophores of other hormurids (Figs. 27, 28, 29, 30, 31, 32, 33). In the phylogeny presented by Sharma et al. [106] *Liocheles* is not retrieved as the most derived hormurid, but is instead placed as a basal scorpionoid, outside of the remaining hormurids, in congruence with the conclusions drawn from morphology of the male copulatory structures. However, very few scorpionoid taxa were sampled in this study, which omitted important ones such as the family Heteroscorpionidae, and a more extensive phylogenetic analysis is needed to confirm the basal position of the HHL clade in Scorpionoidea as retrieved by Sharma et al. [106].

Interestingly, the phylogenetic placement of the HHL clade outside of Hormuridae is also supported by the morphology of the book lungs. As an internal organ, the book lungs can be considered as a functionally constrained complex structure. They are thus probably prone to more phenotypic stability than external non-functional morphology, because they form a complex system within which the response of individual characters to environmental selection is limited by functional interaction [57–60]. The book lungs present a pattern congruent with that derived from the hemispermatophore morphology. Whereas the book lung lamellae and the posterior spiracle edges of the HHL taxa are completely different from those of other hormurids, they share some similarity with those of diplocentrids and heteroscorpionids/urodacids. *Hormiops*, *Hormurus* and *Liocheles* possess lamellae with arcuate distal edges and posterior spiracle edges covered with hillock-like, flattened or chisel-like structures [94, 200], whereas all others hormurids, as well as hemiscorpiids and scorpionids, have lamellae with distal edges covered with bristles or spines, and the posterior edge of their spiracles is covered with hexagonal tiles [200]. On the other hand, diplocentrids have lamellae with arcuate distal edges and heteroscorpionids/urodacids have flattened chisel-like structures on the posterior edges of spiracle, like the HHL taxa [94; 200].

## Conclusion

The comparative study of hemispermatophores and spermatophores provides new insight into the respective and synergetic roles of natural and sexual selection in the evolution of scorpion copulatory structures. Moreover, morphological similarities between spermatophore capsular patterns of groups previously thought to be distant from each other raise questions about the phylogenetic value of functionally constrained traits and their potential use to assess the reliability of contradicting phylogenetic hypotheses. In the present study, the lack of a robust phylogenetic framework for the order Scorpiones has prevented the elaboration of a sound interpretation of the evolutionary sequence of the male reproductive apparatus. This emphasizes the urgent need for a thorough reassessment of the scorpion phylogenetic relationships based on molecular data that would provide the fundamental layout for an accurate understanding of the evolution of reproductive morphology and other complex character systems, and potentially contribute to uncover and define inherent macroevolutionary trends.

## Additional files

Additional file 1: Appendix 1: Hemispermatophores of extant scorpion families: Matrix of characters pertaining to the structural pattern of the hemispermatophore capsule (Nexus format) used for ancestral state reconstruction on the phylogeny by Stockwell [4]. (TXT 1 kb)

Additional file 2: Appendix 2: Hemispermatophores of extant scorpion families: Matrix of characters pertaining to the structural pattern of the hemispermatophore capsule (Nexus format) used for ancestral state reconstruction on the phylogeny by Coddington et al. [102]. (TXT 1 kb)

Additional file 3: Appendix 3: Hemispermatophores of extant scorpion families: Matrix of characters pertaining to the structural pattern of the hemispermatophore capsule (Nexus format) used for ancestral state reconstruction on the phylogeny by Soleglad & Fet [44] and Solegald et al. [103]. (TXT 1 kb)

Additional file 4: Appendix 4: Hemispermatophores of extant scorpion families: Matrix of characters pertaining to the structural pattern of the hemispermatophore capsule (Nexus format) used for ancestral state reconstruction on the phylogeny by Sharma et al. [104]. (TXT 1 kb)

Additional file 5: Appendix 5: Hemispermatophores of extant scorpion families: Matrix of characters pertaining to the structural pattern of the hemispermatophore capsule (Nexus format) used for ancestral state reconstruction on the phylogeny by Prendini [43]. (TXT 4 kb)

Additional file 6: Appendix 6: Hemispermatophores of extant scorpion families: Material examined. (DOCX 41 kb)

Additional file 7: Appendix 7: Hemispermatophores of extant scorpion families: Illustrations from literature. (XLSX 1310 kb)

Additional file 8 References for Appendix 7. (DOCX 225 kb)

Additional file 9: Appendix 8: Hemispermatophores of extant scorpion families: Matrix of characters pertaining to the structural pattern of the hemispermatophore capsule. (XLSX 1392 kb)

Additional file 10: Appendix 9: Spermatophores of extant scorpion families: Material examined. (XLSX 13 kb)

## Abbreviations

CAFC: Colección de Arácnidos de la Facultad de Ciencias, UNAM, México
CAS: California Academy of Sciences (San Fransisco, U.S.A.)
CBIB: UCLA Congo Basin Institute Bastos (Yaoundé, Cameroon)
CNAN: Colección Nacional de Arácnidos, Instituto de Biología, UNAM (Ciudad de México, México)
EWS-WWF: Emirate Wildlife Society-World Wildlife Fund For Nature
FKPC: František Kovařík’s personal collection (Prague, Czech Republic)
HHL clade: clade composed of the hormurid genera (*Hormiops*(*Hormurus + Liocheles))*
ICSS: Island Conservartion Society (Mahé, Seychelles)
ITESI: Instituto Tecnológico Superior de Irapuato (Gunajuato, México)
MCZH: Museum of Comparative Zoology (Harvard, U.S.A.)
MENR: Ministry of Environment, Natural Ressources & Transport, Division of Nature Conservation (Mont Fleuri, Seychelles)
MHNG: Muséum d’histoire naturelle (Geneva, Switzerland)
NHML: Natural History Museum (London, UK)
QM: Queensland Museum (Brisbane, Australia)
SAMC: Iziko South African Museum (Cape Town, South Africa)
SEMARNAT: Secretaria de Medio Ambiente y Recursos Naturales (Ciudad de México, México)
UNAM: Universidad Nacional Autónoma de México, Distrito Federal (Ciudad de México, México)
VAST: Vietnam Academy of Science and Technology (Hanoi, Vietnam)
WAM: Western Australian Museum (Perth, Western Australia, Australia)
