# Supplementary material for: The male sexual apparatus in the order Scorpiones (Arachnida): a comparative study of functional morphology as a tool to define hypotheses of homology
Source: Front Zool. 2017 Nov 22;14:51. doi: 10.1186/s12983-017-0231-z (PMC5699194; doi:10.1186/s12983-017-0231-z)
Supplement: Supplementary file 6 — Appendix 6: Hemispermatophores of extant scorpion families: Material examined. (DOCX 41 kb) [file 12983_2017_231_MOESM6_ESM.docx]

Appendix 1. Hemispermatophores of extant scorpion families: Material examined. Abbreviations for collections as follows: Colección de Arácnidos de la Facultad de Ciencias, Universidad Nacional Autónoma de México, México (CAFC); Colleción Nacional de Arácnidos, Instituto de Biología, Universidad Nacional Autónoma de México, México (CNAN); California Academy of Sciences, San Francisco, U.S.A. (CAS); František Kovařík Private Collection, Prague, [Czech Republic](https://en.wikipedia.org/wiki/Czech_Republic) (FKPC); Instituto Tecnológico Superior de Irapuato, Gunajuato, México (ITESI); Museum of Comparative of Zoology, Harvard University, Cambridge, MA, U.S.A. (MCZ); Muséum d’histoire naturelle, Genève, Switzerland (MHNG); Natural History Museum, London, U.K. (NHML); Iziko South African Museum, Cape Town, South Africa (SAMC); Queensland Museum, Brisbane, Australia (QM); WAM, Western Australian Museum, Perth, Australia (WAM).

**Family Bothriuridae Simon, 1880**

*Bothriurus bonariensis* (C.L.Koch, 1842). Paraguay (Central) (Par-79/6), Villa del Maestro/ San Lorenzo, Asuncion, 5.X.1979, Mission Zoologique (MHNG), 2♂ (MHNG).

*Bothriurus burmeisteri* Kraepelin, 1894. Argentina, Chubut, Puerto Madryn, 19.XII.1981, A.Kovacs, 1♂ (MHNG).

Brazilobothriurus pantanalensis Monod & Lourenço, 2000. Brazil, Mato Grosso, Corumba, 12.VI.1965, E.Kleber, 2♂ (paratype, MHNG).

*Brachistosternus* (Leptosternus) angustimanus Ojanguren & Roig alsina, 2001. Argentina, Chubut, Las Chapas, 21.XII.1982, A. Kovacs, 1♂ (MHNG); Patagonia, Neugen Province, Piedra de Aquila, 7.II.1981, W.Heinz , 1♂(MHNG).

*Cercophonius squama* (Gervais, 1843). Australia, Brighton, M. Martin, 3♂ (MHNG).

*Lisposoma elegans* Lawrence, 1928. South West Africa [Namibia], Kunune Region, Outjo District, Outjo, I-IV.1926, S.A.Museum Expedition, left hemispermatophore from paralectotype ♂ (SAMC-B6077), the staff was unable to locate the right half.

*Orobothriurus huscaran* Ochoa, Ojanguren Affilastro, Mattoni & Prendini, 2011. Perou, Llanganuco, Quebrada de Queroccocha-catac, 3900m, VI.1981, W.R.Lourenço, 1♂ (paratype, MHNG).

*Urophonius iheringii* Pocock, 1893. Brazil, Porto Alegre, 22.IX.1986, E.K.Bastos, 1♂ (MHNG).

**Family Buthidae C.L. Koch, 1837**

*Androctonus liouvillei* (Pallary, 1924). Maroc, El Faty, N of Taghit, IV.1975, P.M.Brignoli, 2♂ (MHNG).

*Androctonus crassicauda* (Olivier, 1087). Iran, Kashan, 250 km S of Teheran, 22.VII.1973, T.Habibi, 3♂ (MHNG).

*Babycurus buettneri* Karsch, 1886. CAMEROUN, Center Region, Ebogo, S of Mbalmayo, 645m, secondary evergreen forest, on tree trunk, 7.IX.2014, L.Monod & J.-J.Ondhoua, 1♂ (MHNG).

*Buthacus leptochelys* (Ehrenberg, 1829). Algeria, Saoura District, Beni-Abbès-Erg, X.1983, P.M.Brignoli, 3♂ (MHNG).

*Buthus atlantis* Pocock, 1889. Morroco, S Mogador, 23.IV.1979, P.M.Brignoli, 3♂ (MHNG).

*Caribetityus elii* (Armas & Marcano Frondeur, 1992). Dominican Republic, La Vega Province, Loma de Casabito, 1410-1440 m, tropical moist forest, 12.V.1998, D.Huber, 9♂ (MHNG).

*Centruroides gracilis* (Latreille). Colombia, Buga-Tulua, 18.II.1988, W.R.Lourenço, 19♂ (MHNG).

*Compsobuthus maindroni* (Kraepelin, 1900). United Arab Emirates, Wadi Wurrayah National Park, 90-225 m, 15-21.V.2015, night collecting, S.Huber & L.Monod, 1♂ (MHNG).

*Grosphus madagascariensis* (Gervais, 1843). Madagascar, H.de Saussure, 3♂ (MHNG).

*Hottentota Jayakari* (Pocock, 1895), United Arab Emirates, Wadi Wurrayah National Park, 140-224 m, 13-15.V.2015, UV night collecting, S.Huber & L.Monod, 2♂ (MHNG).

*Isometrus* (*reddyanus*) *loebli* Vachon, 1982. Sri Lanka, Anuradhapura District, Irrigation Bungalow, Padaviya, 180ft, 27.II-9.III.1970, Davis & Rowe, 1♂ (MHNG).

*Leiurus quinquestriatus* (Ehrenberg, 1828). Soudan, Kerma, East Desert, I.1981, L.Chaix, 1♂ (MHNG).

*Lychas* sp. Kenya, Tana River District, Sankuri, ca. 25 km NW of Garissa, under bark of trees, 160m, 18.X.1977, V.Mahnert & J.-L.Perret, 1♂ (MHNG).

*Microtityus consuelo* Armas & Marcano Frondeur, 1987. Dominican Republic, Borahona Province, 1.2 km of Ojeda, 7.V.1998, rocky trail, bush, D. Huber, 3♂ (MHNG).

*Odontobuthus doriae* (Thorell, 1876). Iran, Teheran, T.Habibi, 2♂ (MHNG).

*Orthochirus glabrifrons* (Kraepelin, 1903). United Arab Emirates, Wadi Wurrayah National Park, 90 m, 13-17.V.2015, coastal flood plain with *Acacia* trees (small gravel and loam), under rocks and at night at the base of tree, on tree trunk, S.Huber & L.Monod, 1♂ (MHNG).

*Parabuthus brevimanus* Thorell, 1877. Namibia, Erongo, ca. 50 km SW Uis, close to Henties Bay, 400-700m, 24-25.II.2012, W.Heinz, 1♂ (MHNG).

*Parabuthus kraepelini* Werner, 1902. Namibia, Karibib, Ombujombenge Farm, 30 km S of Karibib, 1316 m, P. Schmitz, 1♂ (MHNG).

*Rhoplaurus abudi* Armas & Marcano Fondeur, 1987. Dominican Republic, Perdernales Province, 3.9 km N of Manuel Goja, 8.V.1998, sand bush, D.Huber, 1♂ (MHNG).

*Tityus crassimanus* (Thorell, 1876). Dominican Republic, Pedernales Province, Sierra de Bahoruco, Pedernales, km 24 of road Cabo Rojo – El Aceitillar, 782 m , Locality No. 14DR, semicaducifolious forest on karstic soil, F.Kovařík, 2♂ (MHNG).

*Tityus fasciolatus* Pessôa, 1935. Brazil, Distrito Federal, Brasilia, 20.X.1975, W.R.Lourenço, 9♂ (MHNG).

*Tityobuthus griswoldi* Lourenço, 2000. Madagascar, Fianarantsoa Province, Ranomafana National Prac, Talatakely, 19-30.IV.1998, at night, C.E.Griswold, D.H.Kavanaugh, N.D.Penny & M.J.Raherilalao, J.S.Ranorianarisoa, J.S.Schweikert & D.Ubick, 1♂ (paratype, (MHNG).

*Uroplectes occidentalis monardi* Vachon, 1950. Angola, Lunda Norte Province, Nzagi-Andrada, VIII.XII.1952, M.Petchkowsky, 1♂ (MHNG).

**Caraboctonidae** [**Kraepelin**](https://fr.wikipedia.org/wiki/Karl_Matthias_Friedrich_Magnus_Kraepelin)**,** [**1905**](https://fr.wikipedia.org/wiki/1905)

*Caraboctonus keyserlingi* Pocock, 1893. Chili, pet trade import, 2♂ (MHNG).

*Hadruroides mauryi* Francke & Soleglad, 1980. Peru, Huanta, 2560 m, 2011 (locality No. 15), M. Černička, 4♂ (FKPC).

Hadruroides sp. Ecuador, SE, E of Pasaje, ruta 80, E of Machala, 1500 m, W Santa Isabel, 2.II.2011, M. Snížek, 23♂ (FKPC).

*Hadrurus* sp. USA, no precise locality, 1♂ (MHNG).

*Hadrurus* sp. USA, California, F. Nobile, 1♂ (MHNG).

**Family Chactidae Pocock, 1893**

*Anuroctonus phaiodactylus* (Wood, 1863). USA, Utah, Millard Co., ca. 60 mi SE Garrison, V.1994, Van Zee, 1♂ (FKPC).

*Brotheas gervaisii* Pocock, 1893. Brazil, Amapá, Serra de Navio, X.1973, J.Lacroix, 7♂ (MHNG).

*Brotheas granulatus* Simon, 1877. Brazil, Amapá, Serra de Navio, X.1973, J.Lacroix, 18♂ (MHNG).

*Broteochactas nitidus* Pocock, 1893. Trinidad W.I., Mt St. Benedict-Tunapuna, Mt Tabor Trail, 11.III.1997, D.Huber, 1♂ (MHNG).

*Chactas* sp. Columbia, Santa Marta, 1981, Werding, 1♂ (MHNG).

*Chactas vanbenedenii* (Gervais, 1843). Columbia, Dept. Valle del Cauca, Tulua, 973 m, 6.XI.1988, forest, W.R.Lourenço, 1♂ (MHNG).

*Hadrurochactas schaumii* (Karsch, 1880). Surinam, N of Benzdorp, 18-25.III.1987, J.-C.Lacroix, 2♂ (MHNG).

*Neochactas gaillardi* (Lourenço, 198). No precise locality, 1♂ (MHNG).

*Teuthraustes dubius* (Borelli, 1899). Ecuador, Morona Santiago, Macas, IV.1987, G.Onore, 3♂ (MHNG).

*Teuthraustes atramentarius* Simon, 1878. Ecuador, Imbabura, Chachimbiro, 18.XI.1984, S.Abedrabbo, 3♂ (MHNG)

*Uroctonus mordax* Thorell, 1876. USA, California, Trinity County, Rush Creek, ca. 800m. Near Weaverville, 10-11.VII.2002, W. Heinz, 1♂ (MHNG); California, Contra Costa County, Castle Rocks Park, 5 miles W pf mt Diablo, 20.VI.1969, oak woodland, F. Ennik coll., W.J.Gertsch leg., 3♂ (MCZ 15924).

**Family Chaerilidae Pocock, 1893**

*Chaerilus phami* Lourenço, 2011. VIETNAM, Con Dao N.P., Con Son Island, 67m, rainforest, in leaf litter, 11.I.2012, L.Monod, 1♂ (MHNG).

*Chaerilus variegatus* Simon, 1877. Indonesia, Jawa Tengah, Purworejo Regency, Kaligesing District, Somongari, 150m, 27.IV.2013, durian plantation, under stones close to stream, L.Monod & C.Rahmadi, 1♂ (MHNG).

**Family Diplocentridae Karsch, 1880**

*Diplocentrus coylei* Fritts & Sissom, 1996*.* México, Guerrero, Municipio Taxco, 12 km east Taxco, 1611m, 10.V.2014, 1611 m, tropical deciduous forest, under rock, E. González-Santillán & A. Bolzern, 1♂ (CAFC-SC001).

*Diplocentrus lindo* Stockwell & Baldwin, 2001. Mexico, Nuevo Leon, near Doctor Arroyo, ca. 1400 m, 19.VII.1990, W.Heinz, 1♂ (MHNG).

*Diplocentrus zacatecanus* Hoffmann, 1931. México, Aguascalientes, Municipio Tepezalá, 1 km N of Tepezalá, 2048 m, 4.VII.2005, O. Francke, J. Ponce-Saavedra, M. Córdova, A. Jaimes, G. Francke, A. Capovilla. 1♂ (CNAN-1733).

*Nebo poggesii* Sissom, 1994. Yemen S, Ghaye Bawazir, 17-18.IV.1994, B.Schätti, 1♂ (MHNG).

*Nebo yemenensis* Francke, 1980. Yemen S, Province Taizz, ad Dimnah- ar Rahidah, al-Jashaih, Wadi Warazan, 19.VI.1995, B.Schätti, 1♂ (MHNG).

**Family Euscorpiidae Laurie, 1896**

*Euscorpius avcii* Tropea, Yağmur, Koç, Yeşilyurt & Rossi, 2012. Greece, Samos, NE of Megalo Seitani, 327 m, patch of pine forest along dry creek, under stones, 5.VI.2013, L.Monod, 1♂ (MHNG).

*Euscorpius flavicaudis* (De Géer, 1778). France, Alpes-de-Haute-Provence (04), N Peyruis, 420 m, 10.IX.2016, roadside, open woodland on slope, in rock crevices, L.Monod, 1♂ (MHNG).

*Euscorpius italicus* (Herbst, 1800). Switzerland, Wallis, Sion, IX.2013, L.Monod, 1♂ (MHNG).

*Megacormus* sp. México, Veracruz, Calcahualco Minicipality, Pico de Orizaba National Park, 21-30.V.2012, 2388 m, F. Alvarez, V. Garcilazo, D.F.Piedra, A.F.Rivera, F.G.Salgutro, E.González-Santillán leg., 1♂ (MHNG).

**Family Hemiscorpiidae Pocock, 1893**

*Hemiscorpius acanthocercus* Monod & Lourenço, 2005. Iran, Austrian Iran Expedition 1949-1950, H.Löffler, 1♂ (paratypes, MHNG).

*Hemiscorpius lepturus* Peters, 1861. Iran, Khuzestan, Ahwaz region, 21.XI.1995, B.Masihpour, 2♂ (MHNG).

*Hemiscorpius maindroni* Kraepelin, 1900. United Arab Emirates, Wadi Wurrayah NP, headquarters, 90 m, 17.V.2015, coastal flood plain with Acacia trees (small gravel and loam), UV night collecting, S.Huber & L.Monod, 1♂ (MHNG).

**Family Heteroscorpionidae Kraepelin, 1905**

*Heteroscorpion goodmani* Lourenço, 1996. Madagascar, 19-28.X.1995, S.Goodman, 1♂ (paratype, MHNG).

*Heteroscorpion opisthacanthoides* (Kraepelin, 1896). Madagascar, Nosy Be, X.1994, S.Goodman & W.Lourenço, 1♂ (MHNG).

**Family Hormuridae Laurie, 1896**

*Cheloctonus* sp. South Africa, Eastern Cape Province, Sani Pass, near Himeville, 1800-2000 m, 1-2.XII.2009, W.Heinz, 2♂ (MHNG).

*Chiromachus ochropus* (Koch, 1837). Seychelles, Frigate Island, palm forest (seasonally dry) 93 m, 16.VII.2008, under logs and rocks partially buried on the ground, L.Monod, 1♂ (MHNG).

*Hadogenes hahni* (Peters, 1862). Angola, Huila region, Fazenda Bumbo, near Capangombe, 21-26.VI.1954, W.Kiskeret & H.Barmann, 1♂ (MHNG).

*Hadogenes* cf. *paucidens* Pocok, 1896. Tanzania, pet trade import, 5♂ (MHNG).

*Hadogenes troglodytes* (Peters, 1861). South Africa, Kruger National Park, 1073m, 5.IV.2001, rocky outcrop, under flakes of rock, L.Monod & B.Striffler, 1♂ (MHNG).

*Hormiops davidovi* Fage, 1933. Vietnam, Con Dao N.P., Con Son Island, 154m, rainforest, in rock crevices (granitic boulders), 10.I.2012, L.Monod, 1♂ (MHNG).

*Hormiops infulcra* Monod, 2014. W-Malaysia, Tioman Island, 67m, rainforest, in rock crevices (granitic boulders), 25.I.2012, L.Monod, 1♂ (types, MHNG).

*Hormurus longimanus* (Locket, 1995). Australia (NT), Kakadu National Park, Jim Jim Fall, 129 m, monsoon forest, in rock crevices, 21–22.VIII.2009, G.Brown & L.Monod, 1♂ (MHNG).

*Hormurus neocaledonicus* (Simon, 1877), New Caledonia, Monts Koghis, 440 m, 26.IX.2004, rainforest, under rocks, D.Gaillard & L.Monod, 3♂ (MHNG).

*Hormurus polisorum* ([Volschenk](https://fr.wikipedia.org/w/index.php?title=Erich_S._Volschenk&action=edit&redlink=1), [Locket](https://fr.wikipedia.org/w/index.php?title=Nicholas_A._Locket&action=edit&redlink=1) & [Harvey](https://fr.wikipedia.org/wiki/Mark_Stephen_Harvey), [2001](https://fr.wikipedia.org/wiki/2001)), Christmas Island, Bishops Cave, top of rock pile, first main chamber, 120m from light, 11.VIII.1987, N.Plumley, 1 ♂ (holotype, WAM 97/2938).

*Iomachus malabarensis* Pocock, 1900. India, Karnataka, Mangalore, R.C.Wroughton, Battie coll., 3♂ (types, NHML 1896.7.30.75-80).

*Iomachus nitidus* Pocock, 1900. India, Kerala (Travancore), Ponmudi, 1♂ (NHML 1899.7.11.2-3).

*Iomachus politus* Pocock, 1896. Tanzania, pet trade import, 1♂ (MHNG).

*Liocheles longimanus* (Werner, 1939). Indonesia, Sumatra, Sumatera Barat, Mangani, mine near Kota Tingge, 700 m, 21.VII.1983, E.S.Ross, 1♂ (CAS).

*Liocheles nigripes* (Pocock, 1897). India, West Bengal, Janida, Jhargram, Midnapore, 30.VIII.1965, C.L.Kau coll., H.L.Stahnke leg., 8♂ (CAS).

*Opisthacanthus* (*Nepabellus*) *asper* (Peters, 1861). Mozambic, VII.1913, L. Ralli coll., 2♂ (MHNG). South Africa, Natal Province, Mikuki Game Reserve, 1.I.1982, B.Lamoral, 1♂ (MHNG).

*Opisthacanthus* (*Nepabellus*) cf. *rugiceps* Pocock, 1897. Tanzania, pet trade import, 1♂ (MHNG).

*Opisthacanthus* (*Nepabellus*) cf. *validus* Thorell, 1876. South Africa, Eastern Cape Province, Drakensberge, Qacha's Nek, 1900-2000m, 24.XII.2011, W. Heinz, 1♂ (MHNG).

*Opisthacanthus* (*Nepabellus*) *validus* Thorell, 1876. South Africa, Kwazulu-Natal, pet trade import, 2♂ (MHNG).

*Opisthacanthus* (*Opisthacanthus*) *cayaporum* Vellard, 1932. Brazil, Para, Campos do Cayapos, II.1979, W.R.Lourenço, 2♂ (MHNG).

*Opisthacanthus* (*Opisthacanthus*) *elatus* (Gervais, 1844). Colombia, Otanche (Boyaca), 100 km W of Tunja, VI.1986, collected by Indians of the Muzo Mine, W.R.Lourenço, 1♂ (MHNG).

*Opisthacanthus* (*Opisthacanthus*) *lecomtei* (Lucas, 1858). Cameroun, South Region, Nkolandom, S of Ebolowa, 581m, 30.VIII.2014, primary evergreen forest, in cracks of large logs, L.Monod, G.Cuccodoro & J.E.Amougou, 2♂ (MHNG).

*Opisthacanthus* (*Monodopisthacanthus*) *maculatus* Lourenço & Goodman, 2006. Madagascar, Toliara Province, pet trade import, 3♂ (MHNG).

*Opisthacanthus* (*Monodopisthacanthus*) *madagascariensis* Kraepelin, 1894. Madagascar, Toliara Province, pet trade import, 4♂ (MHNG).

*Palaeocheloctonus pauliani* Lourenço, 1996. Madagascar, SW Plateau Mahafaly (near Bahahitso), I.1966, P.Griveaud, 1♂ (paratypes, MHNG).

**Family Iuridae Thorell, 1876**

*Iurus kinzelbachi* Kovařík, Fet, Soleglad & Yağmur, 2010. Greece, Samos, Ambelos, 330 m, oak forest along creek, under stones near cave entrance, 24.IX.2013, L.Monod, 1♂ (MHNG).

*Protoiurus kraepelini* (von Ubisch, 1922). Turkey, Phaselis, 3 km from Tekirova, 25.V.1993, under stones, P.J.Haymoz, 2♂ (MHNG).

**Family Scorpionidae Latreille, 1802**

*Scorpio fuscus* (Ehrenberg, 1828). Israel, Upper Galilea, near Zefat, 700-900m, 1.V.1985, W.Heinz, 1♂ (MHNG).

*Scorpio maurus maurus* Linnaeus, 1758, Algeria, Kabylia, Kebouche, 1100 m, E of Tizi-Ouzon (Ya Kouren), 8.III.1991, W.Heinz, 1♂ (MHNG).

*Pandinus* (*Pandinoides*) *cavimanus* (Pocock, 1888). Tanzania, pet trade import, 1♂ (MHNG).

*Pandinus* (*Pandinus*) *imperator* (C. L. Koch, 1841). Ivory Coast, Man, Centre Bethanie, 13.X.1980, 1♂ (MHNG).

*Heterometrus indus* (De Geer, 1778). Sri Lanka, M.Rugnier, 1♂ (MHNG).

*Heterometrus longimanus* (Herbst, 1800). Sumatra, 2♂ (MHNG).

*Heterometrus mysorensis* [Kovařík](https://fr.wikipedia.org/wiki/Franti%C5%A1ek_Kova%C5%99%C3%ADk_%28arachnologiste%29), [2004](https://fr.wikipedia.org/wiki/2004). No precise locality, pet trade import, 3♂ (MHNG).

*Opisthophthalmus capensis* (Herbst, 1800). South Africa, Western Cape Province, Cape Town, Signal Hill, 13.IV.2001, in burrows, L. Monod, L. Prendini & B. Striffler, 2♂ (MHNG).

*Opisthophthalmus macer* Thorell, 1876. South Africa, Western Cape Province, S of Sir Lowry's Pass, 13.IV.2001, burrows in open loamy area, L.Monod, L.Prendini & B.Striffler, 3♂ (MHNG).

**Family Scorpiopidae Kraepelin, 1905**

*Alloscorpiops* sp. Thailand, Erawan National Park, Bambou forest in rock crevices, 7-9.IX.2001, L.Monod, 3♂ (MHNG).

*Scorpiops* sp. Pakistan, Nathiagali, 2400 m, near Murree, 12.VII.2001, W. Heinz, 1♂ (MHNG).

*Scorpiops* sp. Myanmar, NE of Mandalay, evergreen gallery forest, 990m, 10.VII.2014, S.Huber & P.Schwendinger, 1♂ (MHNG).

*Scorpiops* sp. Thailand, Phitsanulok Province, 1290m, sifting in evergreen hill forest near stream, 23.XII.2013, P.Schwendinger, 1♂ (MHNG).

**Family Troglotayosicidae Lourenço, 1998**

*Belisarius xambeui* Simon, 1879. France, Pyrénées Orientales (66), Arles sur Tech, 367 m, dense forest in gorge, under stones in scree slopes with thick leaflitter, 14.IX.2013, L.Cauwet, T.Andriollo & L.Monod, 1♂ (MHNG).

**Family Typhlochatidae** [**Mitchell**](https://fr.wikipedia.org/w/index.php?title=Robert_W._Mitchell&action=edit&redlink=1)**,** [**1971**](https://fr.wikipedia.org/wiki/1971)

*Alacran triquimera* Santibañez-López, Francke & Prendini, 2014. México, Puebla, Municipio Tecolotec de Díaz, Cueva las Tres Quimeras, 1440 m, 6.IV.2009, B.Shade, ♂ (paratype CNAN-T0870).

**Family Urodacidae Pocock, 1893**

*Urodacus manicatus* (Thorell, 1876). Australia, Victoria, Arapils n. p., 26.III.1997, F.Štáhlavský, 2♂ (FKPC).

*Urodacus* cf. *manicatus* (Thorell, 1877). Australia (SEQ), Koy Property at Brigooda (Bottom site), 26.I-20.IV.1995, vine scrub, pitfall trap, G.B.Monteith, 1♂ (QM 46413).

*Urodacus hoplurus* Pocock, 1898. Australia (WA), Lake Berlee, 29.I.2003, L.Hovorka, 1♂ (FKPC).

**Family Vaejovidae Thorell, 1876**

*Franckeus nitidulus* (C. L. Koch, 1843). México, Hidalgo, Municipio Tasquillo, Puente de Fierro de Tasquillo, 4 km NW of Tasquillo, 1665 m, desert, under rocks, E.González-Santillán, 1♂ (CAFC-S0032).

*Paravaejovis spinigerus* (Wood, 1863). USA, New Mexico, Hidalgo County, pass through Peloncillo Mts, 11 miles S of Road Forks, 8.VIII.1991, T.G.Anton & D.S.Dunkerley coll., W.D.Sissom leg.,1♂ (MCZ 15790); Arizona, Pima County, Coronado National Forest, Madera Canyon, 12.VII.2016, open woodland, UV light hunting, E. González-Santillán & L.Monod, 2♂ (MHNG).

*Paruroctonus utahensis* (Wiliams, 1968). USA, New Mexico, Valencia, roadside, NM 47, 3.2 miles S of junction with NM 304 (vicinity of Belen), 12.VIII.1991, D.S.Dunkerley, T.G.Anton coll., W.D.Sissom leg., 2♂ (MCZ 15845).

*Paruroctonus silvestrii* (Borelli, 1909). USA, California, Marin County, Arroyo Seco, Los Padres National Forest, 1000 ft, rock rolling in grassy valley, 5.V.1968, Bill Butterworth, 1♂ (MCZ 15921).

*Pseudouroctonus apacheanus* (Gertsch & Soleglad, 1972). USA, Arizona, Cochise County, Coronado National Forest, Chiricahua Mountains, 1526m, 11.VII.2016, canyon with large granitic boulders, orientation N>S, forest predominently with oak, along dry creek, E. González-Santillán & L.Monod, 1♂ (MHNG).

*Pseudouroctonus santarita* Ayrey & Soleglad, 2015. USA, Arizona, Pima County, Coronado National Forest, Madera Canyon, 12.VII.2016, mixed forest, under stones, E. González-Santillán & L.Monod, 1♂ (MHNG).

*Smeringurus vachoni* (Stahnke, 1961). USA, California, Inyo County , Saline Valley, Racetrack Valley Rd., Station 64, 1950 ft, pit fall trap, 15.VIII.1959, B.H.Banta, 1♂ (MCZ 15920).

*Uroctonites huachuca* (Gertsch & Soleglad, 1972). USA, Arizona, Cochise County, Coronado National Forest, Huachuca Mountains, Carr Canyon road, 2057m, 13.VII.2016, on rock rock walls along the road, E. González-Santillán, L.Monod & W.Savary, 1♂ (MHNG).

*Vaejovis cashi* Graham, 2007. USA, Arizona, Cochise County, Coronado National Forest, Chiricahua Mountains, 1526m, 11.VII.2016, canyon with large granitic boulders, orientation N>S, forest predominently with oaks, along dry creek, E. González-Santillán & L.Monod, 1♂ (MHNG).

*Vaejovis dugesi* Pocock, 1902. México, Guanajuato, Municipio San Felipe, Sierra de Lobos, 2565 m, 4.vii.2015, oak forest, near waterfall, under rock, J.M.González-Ruíz, 1♂ (ITESI-S046).

*Vaejovis grahami* Ayrey & Soleglad, 2014. USA, Arizona, Pima County, Coronado National Forest, Madera Canyon, 1583 m, 12.VII.2016, mixed forest, under stones, E. González-Santillán & L.Monod, 1♂ (MHNG).

*Vaejovis vorhiesi* Stahnke, 1940. USA, Arizona, Cochise County, Coronado National Forest, Huachuca Mountains, Carr Canyon road, 2057m, 13.VII.2016, in pine forest, E.González-Santillán, L.Monod & W.Savary, 1♂ (MHNG).
