# Supplementary material for: The male sexual apparatus in the order Scorpiones (Arachnida): a comparative study of functional morphology as a tool to define hypotheses of homology
Source: Front Zool. 2017 Nov 22;14:51. doi: 10.1186/s12983-017-0231-z (PMC5699194; doi:10.1186/s12983-017-0231-z)
Supplement: Supplementary file 8 — References for Appendix 7. (DOCX 225 kb) [file 12983_2017_231_MOESM8_ESM.docx]

**REFERENCES TO APPENDIX 2**

**Abalos JW, Hominal CB (1974a)**. *Urophonius achalensis* nueva especie de Bothriuridae. *Acta Zoologica Lilloana*, 31: 19*–*26.

**Abd-el-Wahab A (1957)**. The male genital system of the scorpion, *Buthus quinquestriatus*. *Quarterly Journal of Microscopical Science*, s3*–*98: 111–122.

**Abushama FT (1968)**. Observations on the mating behavior and birth of *Leiurus quinquestriatus* (H. & E.), a common scorpion species in the Central Sudan. *Rev. Zool. Bot. Africaines*, 77: 37–43.

**Acosta LE (1985)**. Redescripción de *Urophonius achalensis* Abalos y Hominal, 1974 (Scorpiones, Bothriuridae). *Physis,* 43(104): 5–12.

**Acosta L E (1990)**. El generó *Cercophonius* Peters, 1861 (Scorpiones, Bothriuridae). *Boletin de la Sociedad de Biologia de Concepción, Chile,* 61:7–27.

**Acosta L E (1990)**. El generó *Cercophonius* Peters, 1861 (Scorpiones, Bothriuridae). *Boletin de la Sociedad de Biologia de Concepción, Chile,* 61:7–27.

**Acosta L E, Ochoa J A (2000)**. Nueva especie de *Orobothriurus* Maury del Peru. *Revue arachnologique,* 13(10): 135–144.

**Acosta LE, Peretti AV (1998)**. Complemento a la descripción de *Bothriurus cordubensis* (Scorpiones, Bothriuridae) con anotaciones sobre patrones evolutivos del género en Argentina. *Revue Arachnologique*, 12(10): 95–108.

**Acosta LE, Ochoa JA (2001)**. Two new species of *Orobothriurus* Maury, 1976 from Argentina and Peru, with comments on the systematics of the genus (Scorpiones: Bothriuridae). Pp. 203–214. In: Fet V, Selden PA (Eds.). In Memoriam Gary A.Polis. British Arachnological Society, Burnham Beeches, UK.

**Alexander AJ (1956)**. Mating in scorpions. *Nature*, 178: 867–868.

**Alexander AJ (1957)**. The courtship and mating of the scorpion *Opisthophthalmus latimanus*. *Proceedings of the Zoological Society of London,* 128: 529–544.

**Alexander A J (1959)**. Courtship and mating in the buthid scorpions. *Proceedings of the Zoological Society of London,* 133(1): 145–169.

**Althaus S, Jacob A, Graber W, Hofer D, Nentwig W, Kropf C (2010)**. A double role of sperm in scorpions: The mating plug of *Euscorpius italicus* (Scorpiones: Euscorpiidae) consists of sperm. *Journal of Morphology,* 271: 383–393.

**Angermann H (1955)**. Indirekte Spermatophorenübertragung bei *Euscorpius italicus* (Herbst). *Naturwissenschaften*, 42: 303.

**Angermann H (1957)**. Über Verhalten, Spermatophorenbuldung und Sinnesphysiologie von *Euscorpius italicus* Hbst. und verwandten arten. *Zeitschrift fur Tierpsychologie*, 14(3): 276–302.

**Auber M (1963)**. Reproduction et croissance de *Buthus occitanus* Amoreux. *Annales des Sciences Naturelles, Zoologie*, Paris, 12ème série, 5: 273–286.

**Ayrey RE (2012)**. A new *Vaejovis* from the Mogollon Highlands of Northern Arizona (Scorpiones: Vaejovidae). *Euscorpius*, 148: 1–13.

**Ayrey RF (2013)**. A new species of *Vaejovis* from the Mogollon Rim of Northern Arizona (Scorpiones: Vaejovidae). *Euscorpius*, 176: 1–13.

**Ayrey RF (2014)**. A new species of *Vaejovis* from chaparral habitat near Yarnell, Arizona (Scorpiones: Vaejovidae). *Euscorpius*, 188: 1–13.

**Ayrey RF, Soleglad ME (2011)**. A new species of Vaejovis from Prescott, Arizona (Scorpiones: Vaejovidae). *Euscorpius,* 114: 1–15.

**Ayrey RF, Soleglad ME (2014)**. New species of *Vaejovis* from the Santa Rita Mountains, Southern Arizona (Scorpiones: Vaejovidae). *Euscorpius*, 183: 1–13.

**Ayrey, R. F., Soleglad, M. E. (2015)**. A new analysis of the genus *Pseudouroctonus* with the description of two new species (Scorpiones: Vaejovidae). *Euscorpius*, 211: 1–53.

**Ayrey RF, Webber MM (2013)**. A new *Vaejovis* C.L.Koch, 1836, the second known *vorhiesi* group species from the Santa Catalina Mountains of Arizona (Scorpiones, Vaejovidae). *Zookeys*, 270: 21–35.

**Bastawade DB (1992)**. Morphological study of spermatophore of a common Indian scorpion *Mesobuthus tamulus tamulus* (Fabr.). *Records of the zoological Survey of India,* 91(2): 221–225.

**Bastawade DB (1994)**. A study of hemispermatophores in Indian scorpions of the families Chaerilidae, VAejovidae and Ischnuridae. *Records of the zoological Survey of India*, 94(2*–*4): 435*–*437.

**Beutelspacher B PCR, deArmas LF (1998)**. Dos especies nuevas de *Diplocentrus* (Scorpiones: Diplocentridae) del sureste de Mexico. *Revista Nicaraguense de Entomologia*, 45: 17–31.

**Botero-Trujillo R, Flórez DE (2011)**. A revisionary approach of Colombian *Ananteris* (Scorpiones, Buthidae): two new species, a new synonymy, and notes on the value of trichobothria and hemispermatophore for the taxonomy of the group. *Zootaxa,* 2904: 1*–*44.

**Bücherl W (1956)**. Escorpiões e escorpionismo no Brasil. V. Observações sobre o aparelho reproductor masculino e o acasalamento de *Tityus trivittattus* e *Tityus bahiensis*. *Memórias do Instituto de Butantan,* 27: 121*–*155.

**Capes EM (2001)**. Description of a new species in the *nitidulus* group of the genus *Vaejovis* (Scorpiones, Vaejovidae). *Journal of Arachnology*, 29(1): 42*–*46.

**Cauwet L (2014)**. Morphology, function and evolution of male genitalia (hemispermatophores and spermatophores) in the superfamily Scorpionoidea Latreille, 1802 (Chelicerata, Scorpiones). *Master thesis, University of Geneva, 214 pp*.

**Cekalovic T (1968)**. *Phoniocercus sanmartini*, nueva especie de Bothriuridae de Chile. (Scorpionida-Bothiruridae). *Boletin de la Sociedad de Biologia de Concepción, 40*: 63*–*79.

**Cekalovic T (1974)**. Dos nueva especies chilenas del genero *Brachistosternus* (Scorpiones, Bothriuridae). *Boletin de la Sociedad de Biologia de Concepción, 47*: 247*–*257.

**Cekalovic T (1975)**. *Brachistosternus (Leptosternus) negrei* n. sp. de escorpión de Chile (Scorpiones, Bothriuridae). *Brenesia*, 6: 69*–*75.

**Cekalovic T (1976)**. Escorpiofauna del parque botanico Hualpen (Concepción, Chile) con la descripción de *Bothriurus wilhelmi* n.sp. (Arachnida, Scorpiones, Bothriuridae). *Boletin de la Sociedad de Biologia de Concepci*ó*n,* 50: 173*–*181.

**Cekalovic T (1982)**. Los escorpiónes de la Isla Mocha, Chile con la descripción de una nueva especie (Scorpiones, Bothriuridae). *Boletin de la Sociedad de Biologia de Concepción,* 53: 41*–*46.

**Contreras-Félix GA, Francke OF (2014)**. Description of a new species of *Vaejovis* from Michoacán, Mexico (Arachnida: Scorpiones: Vaejovidae). *Revista Mexicana de Biodiversidad*, 85: 24–30.

**Contreras-Félix GA, Francke OF, Bryson Jr RW (2015)**. A new species of the "*mexicanus*" group of the genus *Vaejovis* C.L. Koch, 1836 from the Mexican state of Aguascalientes (Scorpiones: Vaejovidae). *Zootaxa*, 3936: 131–140.

**Contreraz-Félix GA, Santibáñez-López CE (2011)**. *Diplocentrus bicolor* sp.n. (Scorpiones: Diplocentridae) from Jalisco, Mexico. *Zootaxa*, 2992: 61*–*68.

**Couzijn HWC (1981)**. Revision of the genus *Heterometrus* Hemprich & Ehrenberg (Scorpionidae, Arachnidea). *Zoologische Verhandelingen (Leiden),* 184: 1*–*196.

**Fet V, Soleglad, ME, Kovařík F (2009)**. Etudes on Iurids, II. Revision of the genus *Calchas* Birula, 1899 with the description of two new species (Scorpiones: Iuridae). *Euscorpius*, 82: 1–72.

**Francke OF (1979a)**. Spermatophores of some North American scorpions (Arachnida, Scorpiones). *Journal of Arachnology*, 7: 19*–*32.

**Francke OF (1979b)**. Observations on the reproductive biology and life history of *Megacormus gertschi* Diaz (Scorpiones: Chactidae ; Megacorminae). *Journal Of Arachnology,* 7: 223*–*230.

**Francke OF (1982)**. Studies of the scorpion subfamilies Superstitioninae and Typhlochactinae, with description of a new genus (Scorpiones, Chactidae). *Association for Mexican Cave Study Bulletin,* 8: 51*–*61 */ Texas Memorial Museum Bulletin*, 28: 51*–*61.

**Francke OF (2007)**. Alacranes (Arachnida, Scorpiones) de Frontera Corozal, en la selva lacandona, Chiapas, México, con la descripción de una nueva especie de *Diplocentrus* (Diplocentridae). *Revista Mexicana de Biodiversidad*, 78: 68–77.

**Francke OF (2009)**. Description of a new species of troglophile *Pseudouroctonus* (Scorpiones: Vaejovidae) from Coahuila, Mexico. *Texas Memorial Museum Speleological Monographs*, 7*. Studies on the cave and endogean fauna of North America*, V: 11–18.

**Francke OF, Ponce-Saavedra J (2005)**. A new species of *Diplocentrus* (Arachnida: Scorpiones) from Michoacan, Mexico. *Revista Mexicana de* Biodiversidad, 76: 49–53.

**Francke OF, Ponce-Saavedra J (2010)**. A new genus and species of scorpion (Scorpiones: Vaejovidae) from Michoacan, Mexico. *Boletín de la Sociedad Entomológica Aragonesa*, 46: 51–57.

**Francke OF, Quijano-Ravell AF (2009)**. Una especie nueva de *Diplocentrus* (Scorpiones: Diplocentridae) from the state of Michoacán. *Revista Mexicana de Biodiversidad*, 80: 659–663.

**Francke OF, Savary WE (2006)**. A new troglobitic *Pseudouroctonus* Stahnke (Scorpiones: Vaejovidae) from northern México. *Zootaxa*, 1302: 21–30.

**Francke OF, Soleglad ME (1981)**. The family Iuridae Thorell (Arachnida, Scorpiones). *Journal of Arachnology*, 9: 233–258.

**Gantenbein B, Kropf C, Largiadèr CR, Scholl A (2000)**. Molecular and morphological evidence for the presence of a new Buthid taxon (Scorpiones: Buthidae) on the Island of Cyprus. *Revue Suisse de Zoologie*, 107(1): 213–232.

**González-Santillán, E, Alvarez-Padilla F (2015)**. The male of *Megacormus granosus* (Gervais, 1844) with comments on its hemispermatophore (Scorpiones, Euscorpiidae). *ZooKeys*, 504: 75–91.

**González-Santillán E, González-Ruíz JM, Escobedo-Morales LE (2017)**. A new species of *Megacormus* (Scorpiones, Euscorpiidae) from an oak-pine forest in Guanajuato, México with an identification key to the species in the genus. *Zootaxa*, 4299(2): 221–237.

**González-Santillán E, Prendini L (2013)**. Redefinition and revision of the north American vaejovid scorpion subfamily Syntropinae Kraepelin, 1905, wiht description of six new genera. *Bulletin of the American Museum of Natural History*, 382: 1–71.

**González-Santillán E, Prendini L (2015b)**. Systematic revision of the north American syntropine vaejovid scoprions with a subacular tubercle, *Konetontli* González-Santillán and Prendini, 2013. *Bulletin of the American Museum of Natural History*, 397: 1–78.

**González-Santillán E, Prendini L (2016)**. Systematic revision of the north American syntropine vaejovid scorpion genera *Maaykuyak*, *Syntropis*, and *Vizcaino*, with description of the adults of *Syntropis williamsi*. *Bulletin of the American Museum of Natural History*, 405: 1–68.

**Graham MR, Soleglad ME (2007)**. A new scorpion genus representing a primitive taxon of tribe Stahnkeini, with a description of a new species form Sonora, Mexico (Scorpiones: Vaejovidae). *Euscorpius*, 57: 1–13.

**Graham MR, Ayrey RF, Bryson Jr RW (2012)**. Multivariate methods support the distinction of a new highland *Vaejovis* (Scorpiones: Vaejovidae) from the Sierra de los Ajos, Mexico. *Journal of Arachnology*, 40: 281–290.

**Hendrixson BE (2001)**. A new *Vaejovis* (Scorpiones, Vaejovidae) from Sonora, Mexico. *Journal of Arachnology*, 29: 47–55.

**Jacob A, Gantenbein I, Braunwalder M E, Nentwig W, Kropf C (2004a)**. Morphology and function of male genitalia (spermatophores) in *Euscorpius italicus* (Euscorpiidae, Scorpiones): complex spermatophore structures enable sperm transfer. *Journal of Morphology*, 260: 72*–*84.

**Jacob A, Gantenbein I, Braunwalder M E, Nentwig W, Kropf C (2004b)**. Complex male genitalia (hemispermatophores) are not diagnostic for cryptic species in the genus *Euscorpius* (Scorpiones: Euscorpiidae). *Organism, Diversity, Evolution*, 4: 59*–*72.

**Jarvis LR, Sissom WD, Henson RN (2004)**. Description of the male of *Vaejovis chisos* Sissom (Scorpiones, Vaejovidae) from Texas, U.S.A., with comments on morphometric and meristic variation in the species. *Entomological News*, 115 (4): 207–211.

**Karataş A, Gharkheloo MM (2012)**. A new *Hemiscorpius* Peters, 1861 (Scorpiones: Hemiscorpiidae) from southwestern Iran. *Turkish Journal of Zoology*, 37: 1*–*9.

**Koch LE (1977)**. The taxonomy, geographic distribution and evolutionary radiation of Australo-Papuan scorpions. *Records of the Western Australian Museum*, 5 (2): 83–367.

**Kovařík F, Fet V, Soleglad ME, Yağmur EA (2010)**. Etudes on Iurids, III. Revision of the Genus Iurus Thorell, 1876 (Scorpiones: Iuridae), with a Description of Two New Species from Turkey. *Euscorpius*, 95: 1–212.

**Kovařík F, Lowe G (2012)**. Review of the genus *Neobuthus* Hirst, 1911 with description of a new species from Ethiopia (Scorpiones: Buthidae). *Euscorpius*, 138: 1–25.

**Kovařík F, Lowe G, Plíšková J, Šťáhlavský F (2013)**. A New Scorpion Genus, *Gint* gen. n., from the Horn of Africa (Scorpiones: Buthidae). *Euscorpius*, 173: 1–31.

**Kovařík F, Soleglad ME, Lowe G, Plíškova J, Sťáhlavský F (2015)**. Observation on growth and maturation of a male *Alloscorpiops wongpromi* (Scorpiones: Euscorpiidae). *Euscorpius*, 206: 1–19.

**Kovařík F, Lowe G, Hoferek D, Plíšková J, Šťáhlavský F (2016a)**. Scorpions of Ethiopia. Part IV. Genus *Uroplectes* Peters, 1861 (Scorpiones: Buthidae). *Euscorpius,* 217: 1–14.

**Kovařík F, Lowe G, Plíšková J, Šťáhlavský F (2016b)**. Scorpions of the Horn of Africa (Arachnida: Scorpiones). Part VI. *Compsobuthus* Vachon, 1949 (Buthidae), with a description of *C. eritreanus* sp. n. *Euscorpius*, 226: 1–21.

**Kovařík F, Lowe G, Šťáhlavský F (2016c)**. Scorpions of the Horn of Africa (Arachnida: Scorpiones). Part IX. *Lanzatus*, *Orthochirus*, and *Somalicharmus* (Buthidae), with description of *Lanzatus somalilandus* sp. n. and *Orthochirus afar* sp.n*. Euscorpius*, 232: 1–38.

**Kovařík F, Lowe G, Šťáhlavský F (2016d)**. Review of the Northwestern African *Buthacus*, with description of *Buthacus stockmanni* sp. n. from Morocco and Western Sahara (Scorpiones, Buthidae). *Euscorpius*, 236: 1–18.

**Kovařík F, Lowe G, Soleglad M.E., Plíškova J. (2017a)**. Scorpions of the Horn of Africa (Arachnida: Scorpiones). Part X. *Pandiborellius* stat. n. and *Pandinurus* (Scorpionidae) with Description of Four New Species from Eritrea and Ethiopia, and Review of *Pandinus* Sensu Lato Taxonomy. *Euscorpius*, 238: 1*–*103.

**Kovařík F, Lowe G, Mazuch T, Plíškova J, Šťáhlavský F (2017b)**. Scorpions of the Horn of Africa (Arachnida: Scorpiones). Part XI. *Pandinurus kmoniceki* sp. n. (Scorpionidae) from Somaliland. *Euscorpius*, 243: 1*–*14.

**Lamoral BH (1979)**. The scorpions of Namibia (Arachnida: Scorpionida). *Annals of the Natal Museum*, 23(3): 497*–*784.

**Levy G, Amitai P (1980)**. Fauna Palestina. *Arachnida Vol 1, Scorpiones,* Jerusalem: Israel Academy of Sciences and Humanities, 130 pp.

**Locket N A (1990)**. A new genus and species of scorpion from South Australia (Buthidae: Buthinae). *Transactions of the Royal Society of South Australia*, 114(2): 67*–*80.

**Locket N A (1995)**. A new ischnurid scorpion from the Northern Territory, Australia. *Record of the Western Australian Museum, supplement*, 52: 191*–*198.

**Lourenço WR (1981)**. A propos de l’indication d’un néotype pour *Opisthacanthus lepturus* (Palisot de Beauvois), 1805 (Scorpionidae). Revue Arachnologique, 3(2): 45*–*52.

**Lourenço WR (1983)**. Considérations sur les genres *Liocheles*, *Ischnurus*, *Opisthacanthus*, *Hormurus*, *Hadogenes* et *Chiromachus* appartenant à la sous-famille des Ischnurinae (Scorpiones: Scorpionidae). *Annals of the Natal Museum,* Pietermaritzburg, 25(2): 403*–*411.

**Lourenço WR (1985)**. Essai d’interprétation de la distribution du genre *Opisthacanthus* (Arachnida, Scorpiones, Ischnuridae) dans les régions néotropicale et Afrotropicale. Étude taxinomique, biogéographique, évolutive et écologique. Thèse de doctorat d’état en Sciences Naturelles, Université Pierre et Marie Curie, Paris. 287 Pp.

**Lourenço WR (1987)**. Révision systématique des scorpions du genre *Opisthacanthus* (Scorpiones: Ischnuridae). *Bulletin du Muséum national d’histoire naturelle (Paris)* (4), 4: 887*–*931.

**Lourenço WR (1989)**. Rétablissement de la famille des Ischnuridae, distincte des Scorpionidae Pocock, 1893, à partir de la sous-famille des Ischnurinae Pocock, 1893. *Revue Arachnologique,* 8(10): 159*–*177.

**Lourenço WR (1996)**. Faune de Madagascar. 87. Scorpiones (Chelicerata, Scorpiones). *Muséum National d’Histoire Naturelle, Paris*.

**Lourenço WR (1997)**. Considération taxonomiques sur le genre *Chiromachetes* Pocock, 1899 (Chelicerata, Scorpiones, Ischnuridae). *Zoosystema,* 19(1): 81*–*89.

**Lourenço WR (2002)**. Reproduction in scorpions, with special reference to parthenogenesis. Pp. 71*–*85. In: Toft S, Scharff (Eds.). European Arachnology 2000. Aarhus University Press, Aarhus, DK.

**Lourenço WR (2001)**. Nouvelles considérations sur la phylogénie et la biogéographie des scorpions Ischnuridae de Madagascar. *Biogeographica*, Paris, 77(2): 83*–*96.

**Lourenço WR (2003)**. A propos d’une nouvelle sous-espèce géographique pour *Bothriurus rochai* Mello-Leitão (Scorpiones, Bothriuridae). *Acta Biologica Paranaense*, Curitiba, 29 (1*–*4): 117*–*125.

**Lourenço WR, Duhem B (2010)**. The genus *Chaerilus* Simon, 1877 (Scorpiones, Chaerilidae) in the Himalayas and description of a new species. *Zookeys*, 37: 13*–*25.

**Lourenço WR, Fé NF (2003)**. Dscription of a new species of *Opisthcanthus* Peters (Scorpiones: Liochelidae) to Brazilian Amazona. *Revista Ibérica de Aracnología,* 8: 81*–*88.

**Lourenço WR, Goodman SM (2006)**. A reappraisal of the geographical distribution of the genus *Opisthcanthus* Peters, 1861 (Scorpiones: Liochelidae) in Madagascar, including the description of four new species. *Boletin Sociedad Entomolómologica Aragonesa,* 38: 11*–*23.

**Lourenço WR, Goodman SM (2006)**. Scorpions of the Réserve spéciale d’Ankarana, Madagascar, with particular rference to cave-dwelling animals and the description of two new species (Arachnida, Scorpiones). *Zoosystema,* 30(3): 665-679.

**Lourenço WR, Maury EA (1979)**. Quelques considérations sur la systématique du Scorpion brésilien *Bothriurus araguayae* Vellard, 1934 (Bothriuridae). *Bulletin du Muséum National d’Histoire Naturelle*, Paris, 1A(2): 421*–*433.

**Lourenço WR, Monod L (1999)**. Confirmation de la validité du genre *Hormiops* Fage, 1933 avec redescription d’*Hormiops davidovi* Fage, 1933 (Scorpiones, Ischnuridae). *Zoosystema*, 21(2).

**Lourenço WR, Monod L (2000)**. Description of a new genus and species of scorpion (Bothriuridae) from Brazil. *Ekológia* (Bratislava), 19(3):145*–*152.

**Lourenço WR, Andrzejewski V, Cloudsley-Thompson JL (2003a)**. The Life History of *Chactas reticulatus* Kraeplin, 1912 (Scorpiones, Chactidae), with a Comparative Analysis of the Reproductive Traits of Three Scorpion Lineages in Relation to Habitat. *Zoologischer Anzeiger,* 242: 63*–*74.

**Lourenço WR, Goodman SM, Raheriarisena M, Ramilijoana O (2003b)**. Description of the male of *Heteroscorpion magnus* Lourenço & Goodman, 2002 (Scorpiones, Heteroscorpionidae). *Revista Ibérica de Aracnología*, 8: 111*–*115.

**Lourenço WR, Motta PC, de Godoi FSP, De Souza Araùjo J (2004)**. Description of a new species of *Bothriurus* Peters (Scorpiones, Bothriuridae) from the State of Tocantins, Brazil. *Boletín Sociedad Entomológica Aragonesa*, 34: 69*–*72.

**Lowe G (2001)**. A new species of *Compsobuthus* Vachon, 1949 from central Oman (Scorpiones: Buthidae). Pp. 171–177. In: Fet V, Selden PA (Eds.). In Memoriam Gary A.Polis. British Arachnological Society, Burnham Beeches, UK.

**Lowe G (2009)**. A new lithophilic *Compsobuthus* Vachon, 1949 (Scorpiones: Buthidae) from Northern Oman. *Euscorpius*, 90: 1–13.

**Lowe G (2010a)**. Two new *Hemiscorpius* Peters, 1861 (Scorpiones: Hemiscorpiidae) from northern Oman. *Euscorpius*, 91: 1–24.

**Lowe G (2010b)**. New picobuthoid scorpions (Scorpiones: Buthidae) from Oman. *Euscorpius*, 93: 1–53.

**Lowe G (2010c)**. A new species of *Odontobuthus* (Scorpiones: Buthidae) from Northen Oman. *Euscorpius*, 96: 1–23.

**Lowe G (2010d)**. The genus *Vachoniolus* (Scorpiones: Buthidae) in Oman. *Euscorpius*, 100: 1–37.

**Lowe G (2010e)**. Two new species of *Hottentotta* Birula, 1908 (Scorpiones: Buthidae) from northern Oman. *Euscorpius*, 103: 1–22.

**Lowe G, Kovařík F (2016)**. Scorpions of the Horn of Africa (Arachnida, Scorpiones). Part V. Two new species of *Neobuthus* Hirst, 1911 (Buthidae), from Ethiopia and Eritrea. *Euscorpius*, 224: 1–46.

**Lowe G, Yağmur EA, Kovařík F (2014)**. A review of the genus *Leiurus* Ehrenberg, 1828 (Scorpiones: Buthidae) with description of four new species form the Arabian Peninsula. *Euscorpius*, 191: 1–129.

**Mathew AP (1957)**. Mating in scorpions. *Journal of the Bombay Natural History Society*, 54(3): 853–857.

**Mattoni CI (2002a)**. *Bothriurus pichicuy*, nuevo escorpión chileno del grupo *vittatus* (Scorpiones, Bothriuridae). *Iheringia,* 92(4): 81–87.

**Mattoni CI (2002b)**. *Bothriurus picunche* sp. nov., a new scorpion from Chile (Bothriuridae). *Studies on Neotropical Fauna and Environnement*, 37(2): 169–174.

**Mattoni CI (2002c)**. La verdadera identidad de *Bothriurus vittatus* (Guérin-Méneville, 1838) (Scorpiones, Bothriuridae). *Revue Arachnologique*, 14(5): 59–72.

**Mattoni CI (2007)**. The genus *Bothriurus* (Scorpiones, Bothriuridae) in Patagonia. *Insect Systematics & Evolution*, 38: 173–192.

**Mattoni CI, Acosta LE (2005)**. A new species of *Bothriurus* from Brazil (Scorpiones, Bothriuridae). *Journal of Arachnology,* 33: 735–744.

**Mattoni CI, Acosta LE (2006)**. Systematics and distribution of three *Bothriurus* species (Scorpiones, Bothriuridae) from central and northern Chile. *Studies on Neotropical Fauna and Environnement*, 41(3): 235–250.

**Mattoni CI, Peretti AV (2004)**. The giant and complex genital plug of the *asper* group of Bothriurus (Scorpiones, Bothriuridae): morphology and comparison with other genital plugs in scorpions. *Zoologischer Anzeiger*, 243: 75–84.

**Maury EA (1968)**. Aportes al conocimiento de los escorpiónes de la Republica Argentina. II. Algunas consideraciones sobre el género *Bothriurus* en la Patagonia y Terra del Fuego con la descripción de una nueva especie (Bothriuridae). *Physis*, 28(76): 149–164.

**Maury EA (1971)**. Essai d’une classification des sous-familles de scorpions Bothriuridae. *Proceeding of the 5th International Congress of Arachnology, Brno:* 29*–*36.

**Maury EA (1973)**. Los escorpiónes de los systemas serranos de la provincia de Buenos Aires. *Physis*, 32(83): 351–371.

**Maury EA (1974)**. Escorpiofauna chaqueña 1. La verdadera identidad de *Brachistosternus* (*Microsternus*) *ferrugineus* (Thorell 1876) (Bothriuridae). *Physis*, 3(86): 73–84.

**Maury EA (1975a)**. La estructura de espermatoforo en el género *Brachistosternus* (Scorpiones, Bothriuridae). *Physis,* 34(89): 179–182.

**Maury EA (1975b)**. Escorpiónes y escorpiónismo en el Peru. V. *Orobothriurus*, un nuevo género de escorpiónes altoandino (Bothriuridae). *Revista Peruana de Entomologia,* 18(1): 14*–*25.

**Maury EA (1975c)**. Escorpiofauna Patagonica. I. Sobre una nueva especie del género *Timogenes* Simon 1880 (Bothriuridae). *Physis*, 34(88): 65–74.

**Maury EA (1977a)**. Un nuevo *Brachistosternus* de los medanos costeros bonaerenses (Scorpiones, Bothriuridae). *Physis*, 37(93): 169*–*176.

**Maury EA (1977b)**. Comentarios sobre dos especies de escorpiónes del género *Urophonius* (Bothriuridae). *Revista del Museo Argentino de Ciencas Naturales, Entomologia,* 5(7): 143–169.

**Maury EA (1978)**. Escorpiónes y escorpiónismo en el Peru. VII: Nuevos hallzgos y redescripción de *Brachistosternus (Microsternus) andinus* Chamberlin, 1916 (Bothriuridae).

**Maury EA (1980)**. Usefulness of the hemispermatophore in the systematics of the scorpion family Bothriuridae. *Comptes rendus du 9ème Congrès International d’Arachnologie, Vienne*: 335–339.

**Maury EA (1981)**. A New *Bothriurus* from Bolivia (Scorpiones, Bothriuridae). *Bulletin of the American Museum of Natural History*, 170.

**Maury EA (1984)**. Redescripción de *Bothriurus bocki* Kraepelin 1911 (Scorpiones, Bothriuridae). *Journal Of Arachnology*, 12: 351–356.

**Maury EA, Roig Alsina A (1977)**. *Timogenes (T.) haplochirus*, nueva especie de escorpión Bothriuridae. *Physis*, 37(93): 275–280.

**Maury EA, San Martin PR (1973)**. Revalidacion del género *Timogenes* Simon, 1880 (Scorpiones, Bothriuridae). *Physis*, 32 (84): 73–84.

**Monod L (2000)**. Révision systématique du genre *Liocheles* (Ishcnuridae, Scorpiones). Travail de diplome de Master. Université de Genève.

**Monod L (2011a)**. The Liochelidae Fet & Bechly, 2001 (Scorpiones) of the Indo-Pacific Region. Systematics and biogeography. Doctoral thesis, The City University of New York.

**Monod L (2011b)**. Taxonomic emendations in the genus *Liocheles* Sundevall, 1833 (Scorpiones, Liochelidae). *Revue suisse de Zoologie*, 118(4): 723–758.

**Monod L (2014)**. The genus *Hormiops* Fage, 1833 (Hormuridae, Scorpiones), a palaeoendemic of the South China Sea: systematics and biogeography. *Comptes Rendus Biologies, 337: 596–608*.

**Monod L (2015)**. Systematics of the genus *Hormiops* Fage, 1933 (Hormuridae, Scorpiones). *Revue suisse de Zoologie, 122(2): 247–2882*.

**Monod L, Harvey MS, Prendini L (2013)**. Stenotopic *Hormurus* Thorell, 1876 scorpions from the monsoon ecosystems of northern Australia, with a discussion on the evolution of burrowing behaviour in Hormuridae Laurie, 1896. *Revue suisse de Zoologie*, 120(2): 281–346.

**Monod L, Lourenço WR (2005)**. Hemiscorpiidae (Scorpiones) from Iran, with descriptions of two new species and notes on biogeography and phylogenetic relationships. *Revue suisse de Zoologie*, 112(4): 869–941.

**Monod L, Volschenk ES (2004)**. *Liocheles litodactylus* (Scorpiones: Liochelidae): an unusual new *Liocheles* species from the Australian wet tropics (Queensland). *Memoirs of the Queensland Museum*, 49(2): 675–690.

**Newlands G, Prendini, L (1997)**. Redescription of *Hadogenes zumpti* Newlands & Cantrell, 1985: an unusual rock scorpion (Scorpiones, Ischnuridae) from the Richtersveld, South Africa. *South African Journal of Zoology*, 32: 76–81.

**Ochoa JA (2002)**. Nueva especie de *Brachistosternus* Pocock (Scorpiones: Bothriuridae) del sur del Perú. *Revista Peruana de Biología*, 9(2): 55–63.

**Ochoa JA (2004a)**. Filogenia del género *Orobothriurus* y descripción de un nuevo género de Bothriuridae (Scorpiones). *Revista Ibérica de Arachnología*, 9: 43–73.

**Ochoa JA (2004b)**. *Brachistosternus ninapo* una nueva especie (Scorpiones: Bothriuridae) de los Andes occidentales en el sur del Perú. *Revista Peruana de Biología,* 11(2): 139–148.

**Ochoa JA (2011)**. Sobre la identidad taxonómica de *Brachistosternus peruvianus* Piza, 1974 (Scorpiones: Bothriuridae). *Revista Peruana de Biología*, 18(1): 3–12.

**Ochoa JA, Acosta LE, (2002a)**. Two new Andean species of *Brachistosternus* Pocock (Scorpiones: Bothriuridae). *Euscorpius*, 2: 1–13.

**Ochoa JA, Acosta LE (2002b)**. *Orobothriurus atiquipa*, a new bothiurid species (Scorpiones) from Lomas in Southern Peru. *Journal of Arachnology,* 30: 98–103.

**Ochoa JA, Acosta LE (2003)**. Una nueva especie de *Orobothriurus* (Scorpiones: Bothriuridae) del Santuario Nacional Ampay, Apurímac, Perú. *Revista Peruana de Entomología*, 43: 1–6.

**Ochoa JA, Botero-Trujillo R, Prendini L (2010)**. On the troglomorphic scorpion *Troglotayosicus humilicum* (Scorpiones, Troglotayosicidae), with first description of the adults. *American Museum Novitates*, 3691: 1–19.

**Ochoa JA, Chaparro JC (2008)**. Nueva especie de escorpión del género *Hadruroides* (Scorpiones: Caraboctoninae) de los valles interandinos de Perú. *Revista Peruana de Biología*, 15(1): 5–10.

**Ochoa JA, Ojanguren-Affilastro AA (2006).** Systematics and distribution of *Brachistosternus (Brachistosternus) ehrenbergii* (Gervais, 1841), with the first record of stridulation in this genus *Brachistosternus* (Scorpiones: Bothriuridae). *Studies on Neotropical Fauna and Environnement*, 42(1): 61–69.

**Ochoa JA, Prendini L (2010)**. The genus *Hadruroides* Pocock 1893 (Scorpiones: Iuridae) in Peru: New records and descriptions of six new species. *American Museum Novitates*, 3687:1–56.

**Ochoa JA, Ojanguren Affilastro AA, Mattoni CI, Prendini L (2011)**. Systematic revision of the andean scorpion genus *Orobothriurus* Maury, 1976 (Bothriuridae), with discussion of the altitude record for scorpions. *Bulletin of the American Museum of Natural History*, 359: 1–90.

**Ochoa JA, Rosaj-Runjaic FJM, Pinto-Da-Rocha R, Prendini L (2013)**. Systematic revision of the Neotropical scorpion genus *Chactopsis* Kraepelin, 1912 (Chactoidea: Chactidae), with descriptions of two new genera and four new species. *Bulletin of the American Museum of Natural History*, 378.

**Ojanguren-Affilastro AA (2002a).** Descripción de *Bothriurus pampa* n. sp., con nuevas localidades para el grupo p*rospicuus* (Scorpiones, Bothriuridae). *Revista Ibérica de Aracnología*, 6: 95–102.

**Ojanguren-Affilastro AA (2002b).** Nuevos aportes al conocimiento del género *Urophonius* Pocock, 1893 (Scorpiones, Bothriuridae). *Revista Ibérica de Aracnología*, 6: 181–186.

**Ojanguren-Affilastro AA (2005).** Estudio monográfico de los escorpiones de la República Argentina. *Revista Ibérica de Aracnología*, 11: 75–246.

**Ojanguren-Affilastro AA (2007)**. A new endemic scorpion species from the Somuncura Plateau, in northern Patagonia (Scorpiones, Bothriuridae). *Zootaxa*, 1466: 47–56.

**Ojanguren-Affilastro AA (2010)**. A new *Bothirurus* (Scorpiones, Bothriuridae) from the Somuncura Plateau, with additions to the knowledge to the endemic scorpion fauna of the area. *Zootaxa*, 2488: 52–64.

**Ojanguren-Affilastro AA, Garcia-Mauro I (2010)**. A new *Bothriurus* (Scorpiones, Bothriuridae) from the Somuncura Plateau, with additions to the knowledge to the endemic scorpion fauna of the area. *Zootaxa*, 2488: 52–64.

**Ojanguren-Affilastro AA, Mattoni CI (2006).** A new species of *Brachistosternus* from Chilean central Andes (Scorpiones: Bothriuridae). *Studies on Neotropical Fauna and Environment,* 41(1): 79*–*85.

**Ojanguren-Affilastro AA, Mattoni CI (2017)**. *Mauryius* n.gen. (Scorpiones: Bothriuridae), a new neotropical scorpion genus. *Arthropod Systematics & Phylogeny*, 75(1): 125*–*139.

**Ojanguren-Affilastro AA, Agusto P, Pizarro-Araya J, Mattoni CI (2007a)**. Two new scorpion species of genus *Brachistosternus* (Scorpiones: Bothriuridae) from northern Chile. *Zootaxa*, 1623: 55*–*68.

**Ojanguren-Affilastro AA, Campòn FF, Silnik SL, Mattoni CI (2009)**. The genus *Orobothriurus* Maury in central Argentina with description of a new species from El Nevado mountain chain in Mendoza Province (Scorpiones: Bothriuridae). *Zootaxa*, 2209: 28*–*42.

**Ojanguren-Affilastro AA, Mattoni CI, Ochoa JA, Prendini L (2012)**. *Rumikiri*, n. gen. (Scorpiones: Bothriuridae), a new scorpion genus from the Atacama Desert. *American Museum Novitates*, 3731: 1–43.

**Ojanguren-Affilastro AA, Mattoni CI, Prendini L (2007b).** The genus *Brachistosternus* (Scorpiones: Bothriuridae) in Chile, with descriptions of two new species. *American Museum Novitates*, 3564: 1–44.

**Ojanguren-Affilastro AA, Ochoa JA, Mattoni CI, Prendini L (2010)**. Systematic revision of the *granulatus* group of *Urophonius* Pocock, 1893 (Scorpiones, Bothriuridae), with description of a new species from Central Chile. *American Museum Novitates*, 3695: 1–40.

**Peretti AV (1991)**. Comportamiento de apareamiento de *Zabius fuscus* (Thorell) (Buthidae, Scorpiones). *Boletin de la Sociedad de Biología de Concepción*, Concepción, 62:123–146.

**Peretti AV (1992)**. El espermatóforo de *Bothriurus bonariensis* (C.L. Koch, 1843) (Scorpiones, Bothriuridae): morfología y funcionamento. *Boletin de la Sociedad de Biología de Concepción*, Concepción, 63:157–167.

**Peretti AV (1995)**. Structure and function of the hemispermatophore and spermatophore of *Bothriurus flavidus* Kraepelin, 1910 (Scorpiones, Bothriuridae). Iheringia, *Série Zoologia*, Porto Alegre, 78:29–37.

**Peretti AV (1996)**. Compartamiento de transferencia espermática de *Bothriurus flavidus* (Scorpiones: Bothriuridae). *Revista de la Sociedad Entomológica Argentina*, 55: 7–20.

**Peretti AV (2003)**. Functional morphology of spermatophores and female genitalia in bothriurid scorpions: genital courtship, coercion and other possible mechanisms. *Journal of Zoology, London*, 261: 135–153.

**Peretti AV (2010)**. An ancient indirect sex model: single and mixed patterns in the evolution of scorpion genitalia. Pp. 218–248. In: Leonard JL, Córdoba-Aguilar A (Eds). The Evolution of Primary Sexual Characters in Animals. Oxford University Press, Oxford, UK.

**Peretti AV, Acosta LE, Martínez MA (2000)**. Compartamiento de apareamiento en tres especies de *Bothriurus* del grupo *prospicuus*: estudio comparado y su relación con *Bothriurus flavidus* (Scorpiones, Bothriuridae). *Revue arachnologique*, 13(5): 73–91.

**Prendini L (2001)**. Two new species of *Hadogenes* (Scorpiones, Ischnuridae) from South Africa, with a redescription of *Hadogenes bicolor* and a discussion on the phylogenetic position of *Hadogenes. Journal of Arachnology*, 29: 146–172.

**Prendini L (2003)**. A new genus and species of bothriurid scorpion from the Brandberg Massif, Namibia, with a reanalysis of bothriurid phylogeny and a discussion of the phylogenetic position of *Lisposoma* Lawrence. *Systematic Entomology*, 28, 149–172.

**Prendini L. (2004)**. Systematics of the genus *Pseudolychas* Kraepelin (Scorpiones: Buthidae). *Annals of the Entomological Society of America*, 97(1): 37–63.

**Prendini L (2005)**. On *Hadogenes angolensis* Lourenço, 1999 syn. n. (Scorpiones, Liochelidae), with a redescription of *H. taeniurus* (Thorell, 1876). *Revue Suisse de Zoologie,* 112(2): 1–28.

**Prendini L (2006)**. New South African flat rock scorpions (Liochelidae: *Hadogenes*). *American Museum Novitates*, 3502: 1–32.

**Prendini L, Esposito L (2010)**. A reanalysis of *Parabuthus* (Scorpiones: Buthidae) phylogeny with a description of two new *Parabuthus* species endemic to the Central Namib gravel plains, Namibia. *Zoological Journal of the Linnean Society*, 159: 673–710.

**Prendini L, Volschenk E S, Maaliki S, Gromov A V (2006)**. A ‘living fossil’ from Central Asia: the morphology of *Pseudochactas ovchinnikovi* Gromov, 1998 (Scorpiones: Pseudochactidae), with comments on its phylogenetic position. *Zoologischer Anzeiger*, 245: 211–248.

**Prendini L, Esposito LE, Huff JC, Volschenk ES (2009)**. Redescription of *Rhopalurus abudi* (Scorpiones, Buthidae), with first description of the male and first record from mainland Hispaniola. *Journal of Arachnology*, 37: 206–224.

**Probst PJ (1972)**. Zur Fortpflazungsbiologie und zur Entwicklung der Giftdrusen beim Skorpion *Isometrus maculatus* (De geer, 1778). *Acta Tropica*, 29: 1*–*87.

**Rosin R, Shulov A (1963)**. Studies on the scorpion Nebo hierichonticus. *Proceedings of the Zoological Society of London*, 140(4): 547*–*575.

**Sagastume-Espinoza KO, Longhorn SJ, Santibáñez-López CE (2015)**. A new scorpion species of the genus *Diplocentrus* Peters, 1861 (Scorpiones: Diplocentridae) endemic to Islas de la Bahia, Honduras. *Comptes Rendus Biologies*, 338: 502*–*510.

**SanMartin PR (1963)**. Una nueva especie de *Bothriurus* (Scorpiones, Bothriuridae) del Uruguay. *Bulletin du Muséum National d’Histoire Naturelle,* 35: 400*–*418.

**SanMartin PR (1965)**. Escorpiofauna Uruguaya II. *Bothriurus rochensis*, nueva especie de Bothriuridae del Uruguay. *Comunicaciones zoologicas del museo de Historia Natural de Montevideo*, 106(8): 1*–*22.

**SanMartin PR (1968)**. *Bothriurus vachoni*, n.sp. del Brasil (Scorpionida, Bothriuridae). *Acta Biologica Venezuelica,* 6(2): 38*–*51.

**SanMartin PR (1969)**. Estudio sobre la compleja estructura del esqueleto esclerificado del organo paraxial del género *Brachistosternus* (Bothriuridae, Scorpionida). *Boletin de la Sociedad de Biologia de Concepción*, 41: 13*–*30.

**SanMartin PR, Gambardella L A (1974)**. Redescripción de *Urophonius iheringi* Pocock, 1893 y consideraciones sobre morfologia, bioecologia y distribucion. *Boletin de la Sociedad de Biologia de Concepción,* 57: 93*–*119.

**Santibáñez-López CE (2014)**. A new species of the genus *Diplocentrus* Peters, 1861 (Scorpiones, Diplocentridae) from Oaxaca, Mexico. *Zookeys 412: 103–116*.

**Santibáñez-López CE, Francke OF (2008)**. A new species of *Diplocentrus* (Arachnida: Scorpiones) from Oaxaca, Mexico. *Zootaxa 1742: 53–60*.

**Santibáñez-López CE, Francke OF (2010)**. New and poorly known species of the *mexicanus* group of the genus *Vaejovis* (Scorpiones: Vaejovidae) from Oaxaca, Mexico. *Journal of Arachnology*, 38: 555–571.

**Santibáñez López CE, Francke OF (2013)**. Redescription of *Diplocentrus zacatecanus* (Scorpiones: Diplocentridae) and limitations of the hemispermatophore as a diagnostic trait for genus *Diplocentrus*. *Journal of Arachnology*, 41: 1–10.

**Santibáñez-López CE, Francke OF, Athanasiadis MC (2011)**. The genus *Diplocentrus* Peters (Scorpiones: Diplocentridae) in Morelos, Mexico. *Revista Ibérica de Aracnología*, 19: 3–13.

**Santibáñez-López CE, Francke OF, Prendini L (2013)**. Systematics of the *keyserlingii* group of *Diplocentrus* Peters, 1861 (Scorpiones: Diplocentridae), with descriptions of three new species from Oaxaca, Mexico. *American Museum Novitates*, 3777: 1–47.

**Santibáñez-López CE, Francke OF, Prendini L (2014)**. Shining a light into the world's deepest caves: phylogenetic systematics of the troglobiotic scorpion genus *Alacran* Francke, 1982 (Typhlochactidae: Alacraninae). *Invertebrate Systematics*, 28: 643–664.

**Santibáñez-López CE, González Santillán E (2017)**. A new species of *Diplocentrus* (Scorpiones: Diplocentridae) with punctate pedipalp surfacess, a diagnostic character within the «mexicanus» group. *Comptes Rendu Biologies* (https://doi.org/10.1016/j.crvi.2017.03.005).

**Santibáñez-López CE, Sissom WD (2010)**. A new species of the *Vaejovis eusthenura* group in Oaxaca, Mexico (Scorpiones: Vaejovidae). *Zootaxa*, 2493: 49–58.

**Shulov A (1958)**. Observations on the mating habits of two scorpions, *Leiurus quinquestriatus* H. et E. and *Buthotus judaicus* E.S. Pp. 877-880. In: Becker, E.C. (Ed.). Proceedings of the 10th International Congress of Entomology. Montreal, August 17-25, 1956. Volume 1. Mortimer, Montreal, Canada.

**Shulov A, Amitai P (1958)**. On the mating habits of three scorpions: *Leiurus quinquestriatus* H. & F*., Buthotus judaicus* E. Sim. and *Nebo hierichonticus* E. Sim. *Archives de l’Institut Pasteur d’Algérie*, 36: 351–369.

**Sissom WD (1986)**. *Diplocentrus colwelli*, a new species of scorpion from northern Mexico (Diplocentridae). *Insecta Mundi*, 1(4): 255–258.

**Sissom WD (1989)**. Systematic studies on *Vaejovis granulatus* Pocock and *Vaejovis pusillus* Pocock, with descriptions of six new related species (Scorpiones, Vaejovidae). *Revue Arachnologique*, 8(9): 131–157.

**Sissom WD (1990)**. Chap. 3: Systematics, Biogeography, and Paleontology. Pp. 64–160. In: Gary AP (Ed.). The Biology of Scorpions. Standford University Press, Stanford, USA.

**Sissom WD (1991)**. Systematic studies on the *nitidulus* group of the genus *Vaejovis*, with descriptions of seven new species (Scorpiones, Vaejovidae). *Journal of Arachnology,* 19: 4*–*28.

**Sissom WD (1994a)***.* Systematic studies on the genus *Megacormus* (Scorpiones, Chactidae, Megacorminae), with descriptions of a new species from Oaxaca, Mexico and the male of *Megacormus segmentatus* Pocock. *Insecta Mundi,* 8 (3*–*4): 265*–*272.

**Sissom WD (1994b)***.* Descriptions of new and poorly known scorpions of Yemen (Scorpiones: Buthidae, Diplocentridae, Scorpionidae). *Fauna of Saudi Arabia,* 14: 3*–*39.

**Sissom WD (1994c)**. Systematic studies on *Diplocentrus keyserlingii* and related species from Central Oaxaca, Mexico (Scorpiones, Diplocentridae). *Mitteilungen aus dem Zoologischen Museum in Berlin*, 70: 257–266.

**Sissom WD (1993)**. A new species of *Vaejovis* (Scorpiones, Vaejovidae) from western Arizona, with supplementary notes on the male of *Vaejovis spicatus* Haradon. *Journal of Arachnology*, 21: 64–68.

**Sissom WD, Hughes GB, Bryson Jr RW, Prendini L (2012)**. The *vorhiesi* group of *Vaejovis* C.L. Koch, 1836 (Scorpiones: Vaejovidae), in Arizona, with description of a new species from the Hualapai Mountains. *American Museum Novitates*, 372: 1–19.

**Sissom WD, Stockwell SA (1991)**. The genus *Serradigitus* in Sonora, Mexico, with descriptions of four new species (Scorpiones, Vaejovidae). *Insecta Mundi*, 5(3–4): 197–214.

**Sissom WD, Wheeler AL (1995)**. Scorpions of the genus *Diplocentrus* (Diplocentridae) from Sonora, Mexico, with descriptions of a new species. *Insecta Mundi*, 9(3–4): 309–316.

**Sissom WD, Graham MR, Donaldson TG, Bryson Jr RW (2016)**. Two new *Vaejovis* C.L.Koch 1836 from highlands of the Sierra Madre Occidental, Durango, Mexico (Scorpiones, Vaejovidae). *Insecta Mundi*, 477: 1–14.

**Soleglad MA, Fet V (2004)***.* The systematics of the scorpion subfamily Uroctoninae (Scorpiones: Chactidae)*. Revista Ibérica de Aracnología*, 10: 81–128

**Soleglad MA, Fet V (2008)***.* Contribution to scorpion systematics. III. Subfamilies Smeringurinae and Syntropinae (Scorpiones: Vaejovidae)*. Euscorpius*, 71: 1–115.

**Soleglad ME, Fet V, Kovařík F, Yağmur EA (2012)**. Etudes on Iurids, V. Further revision of *Iurus* Thorell, 1876 (Scorpiones: Iuridae), with a description of a new genus and two new species. *Euscorpius*, 142: 1–70.

**Soleglad MA, Fet V, Graham MR (2014)**. *Kovarikia*, a new scorpion genus from California, USA (Scorpiones: Vaejovidae). *Euscorpius*, 185: 1–22.

**Soleglad MA, Fet V, Graham MR, Ayrey RF (2016)**. *Graemeloweus*, a new scorpion genus from northern California, USA (Scorpiones: Vaejovidae). *Euscorpius*, 227: 1–38.

**Soleglad ME, Fet V, Lowe G (2011)**. Contributions to scorpion sysematics. IV. Observations of the *Hadrurus* "*spadix*" subgroup with a description of a new species (Scorpiones: Caraboctonidae). *Euscorpius*, 112: 1–36.

**Soleglad MA, Sissom WD (2001).** Phylogeny of the family Euscorpiidae Laurie, 1896: a major revision. Pp. 25–111. In: Fet V, Selden PA (Eds.). In Memoriam Gary A.Polis. British Arachnological Society, Burnham Beeches, UK.

**Stockwell SA (1988)**. Six new species of *Diplocentrus* Peters from Central America (Scorpiones, Diplocentridae). *Journal of Arachnology*, 16: 153*–*175.

**Stockwell, S A (1989)**. Revision of the phylogeny and higher classification of Scorpions (Chelicerata). Doctoral thesis, University of California, Berkeley.

**Stockwell SA, Baldwin AS (2001)**. A new species of *Diplocentrus* (Scorpiones, Diplocentridae) from Texas. *Journal of Arachnology*, 29: 304*–*311.

**Stockwell SA, Nilsson JA (1987)**. A new species of *Diplocentrus* Peters from Texas (Scorpiones, Diplocentridae). *Journal of Arachnology*, 15: 151–156.

**Tate AE, Riddle RR, Soleglad ME, Graham MR (2013)**. *Pseudouroctonus peccatum*, a new scorpion from the Spring Mountains near "Sin City", Nevada (Scorpiones, Vaejovidae)*. ZooKeys*, 346: 29–45.

**Tropea G, Yağmur EA, Koç H, Yeşilyurt F, Rossi A (2012)**. A new species of *Euscorpius* Thorell, 1876 (Scorpiones, Euscorpiidae) from Turkey. *ZooKeys*, 219: 63*–*80.

**Vachon M (1952)**. Étude sur les scorpions. *Institut Pasteur d’Algérie, Alger*. 482 pp.

**Volschenk ES, Smith GT, Harvey MS (2000)**. A new species of *Urodacus* from Western Australia, with additional descriptive notes for Urodacus megamastigus (Scorpiones). *Records of the Western Australian Museum*, 20: 57*–*67.

**Volschenk ES, Locket NA, Harvey MS (2001)**. First record of a troglobitic ischnurid scorpion from Autralia (Scopriones: Ischnuridae). Pp. 171–177. In: Fet V, Selden PA (Eds.). In Memoriam Gary A.Polis. British Arachnological Society, Burnham Beeches, UK.

**Webber MM, Graham MR, Jaeger JR (2012)**. *Wernerius inyoensis*, an elusive new scorpion from the Inyo Mountains of California (Scorpiones, Vaejovidae). *Zookeys*, 177: 1–13.

**Williams SC, Savary WE (1991)**. *Uroctonites*, a new genus of scorpion from Western North America (Scorpiones: Vaejovidae). *Pan-Pacific Entomologist*, 67: 272–287.

**Yağmur EA, Tropea G (2013)**. A new species of *Euscorpius* Thorell, 1876 (Scorpiones, Euscorpiidae) from Marmara Region of Turkey. *ZooKeys*, 281: 91*–*105.

**Yağmur EA, Tropea G, Yeşilyurt F (2013)**. A new species of *Euscorpius* Thorell, 1876 (Scorpiones, Euscorpiidae) from south western Turkey. *ZooKeys*, 348: 29*–*45.

**Yağmur EA, Soleglad ME, Fet V, Kovařík F (2013)**. Etudes on Iurids, VI. Further revision of *Calchas* Birula, 1899 (Scorpiones: Iuridae), with a description of a new genus and two new species. *Euscorpius*, 159: 1–37.

**Yağmur EA, Soleglad ME, Fet V, Kovařík F (2015a)**. Etudes on Iurids, VIII. A new *Protoiurus* species from the Hıdırellez Cave in Antalya, Turkey (Scorpiones: Iuridae). *Euscorpius*, 200: 1–25.

**Yağmur EA, Kovařík F, Fet V, Soleglad ME, Yeşilyurt F (2015b)**. Etudes on Iurids, IX. Further analysis of a rare species *Protoiurus kadleci* (Scorpiones: Iuridae) from Turkey, based on adult males. *Euscorpius*, 201: 1–18.

**Yamaguti HY, Pinto-da-Rocha R (2003)**. Taxonomic review of the genus *Thestylus* Simon, 1880 (Scorpiones: Bothriuridae). *Revista Ibérica de Aracnología*, 7: 157*–*171.
